# Formation and diversification of a paradigm biosynthetic gene cluster in plants

**DOI:** 10.1038/s41467-020-19153-6

**Published:** 2020-10-23

**Authors:** Zhenhua Liu, Jitender Cheema, Marielle Vigouroux, Lionel Hill, James Reed, Pirita Paajanen, Levi Yant, Anne Osbourn

**Affiliations:** 1 Department of Metabolic Biology, John Innes Centre, Norwich Research Park, Colney Lane, Norwich, NR4 7UH UK; 2 Computational and Systems Biology, John Innes Centre, Norwich Research Park, Norwich, NR4 7UH UK; 3 School of Life Sciences and Future Food Beacon, University of Nottingham, Nottingham, NG7 2RD UK

**Keywords:** Evolutionary genetics, Plant genetics, Secondary metabolism

## Abstract

Numerous examples of biosynthetic gene clusters (BGCs), including for compounds of agricultural and medicinal importance, have now been discovered in plant genomes. However, little is known about how these complex traits are assembled and diversified. Here, we examine a large number of variants within and between species for a paradigm BGC (the thalianol cluster), which has evolved recently in a common ancestor of the *Arabidopsis* genus. Comparisons at the species level reveal differences in BGC organization and involvement of auxiliary genes, resulting in production of species-specific triterpenes. Within species, the thalianol cluster is primarily fixed, showing a low frequency of deleterious haplotypes. We further identify chromosomal inversion as a molecular mechanism that may shuffle more distant genes into the cluster, so enabling cluster compaction. Antagonistic natural selection pressures are likely involved in shaping the occurrence and maintenance of this BGC. Our work sheds light on the birth, life and death of complex genetic and metabolic traits in plants.

## Abstract (web summary)

How plant biosynthetic gene clusters (BGCs) form and evolve remains unclear. Here, via examining available genomes within and between *Arabidopsis* species, the authors show that the thalianol BGC has evolved recently and is still dynamically evolving through involvement of auxiliary genes and chromosomal inversions.

## Introduction

Nonrandom gene organization in eukaryotes plays a significant role in genome evolution and function. Although most eukaryotic genomes lack operons, they do contain groups of physically clustered genes that are related in function despite being unrelated in sequence. Striking examples of this are biosynthetic gene clusters (BGCs)^1,2^. BGCs have been studied extensively in fungi^1,2^. Over the last few years numerous BGCs have also been reported from plants, including for compounds that determine agricultural traits such as disease resistance and drought tolerance^3,4^ or have important pharmaceutical applications^5,6^. There is compelling evidence that these plant clusters have not arisen by horizontal gene transfer from microbes, but instead are likely to be generated through gene duplication, neofunctionalization, and genomic relocation^1,2^. However, very little is known about how these gene clusters form. Several examples of syntenic BGCs that make variants of the same types of molecules have been reported when different species are compared, including for steroidal glycoalkaloids in the Solanaceae and triterpenes associated with bitterness in the Cucurbitaceae^7,8^. However, large-scale analysis of the evolutionary dynamics of individual BGCs within and across species within appropriate taxonomic windows has been hindered by the lack of available sequence resources.

Understanding the clustering of functionally related nonhomologous genes is crucial to our understanding of the relationship between genome organization and the evolution of complex adaptive traits. Over 10 years ago we characterized an “operon-like” gene cluster from the model plant species *Arabidopsis thaliana* (the thalianol BGC)^9^. More recently we have shown that this cluster plays an important role in shaping the root microbiota^10^.

Here, we take advantage of the rich resource of recently generated sequence data for *A. thaliana* and its relatives to investigate the occurrence, nature and evolution of this paradigm BGC at both the vertical (between species) and horizontal (within species) levels. Our results reveal that the thalianol BGC has evolved specifically in the *Arabidopsis* genus, and is still dynamically evolving between sister species through involvement of distant auxiliary genes. Within the *A. thaliana* species, the thalianol BGC is primarily fixed, as demonstrated by its conserved biochemical function and evidence of pervasive purifying selection imposed on individual BGC genes. In contrast, signatures for localized positive selection are identified when considering the cluster region as a whole. Analysis of de novo genome sequence assemblies of *A. thaliana* accessions from around the world further reveals compaction of the thalianol BGC through chromosomal inversions. The divergent and polymorphic cluster variants that we identify provide new insights into the birth, life and death of BGCs in plants.

## Results

### The biochemical function and evolutionary origin of the thalianol cluster

**Fig. 1:**
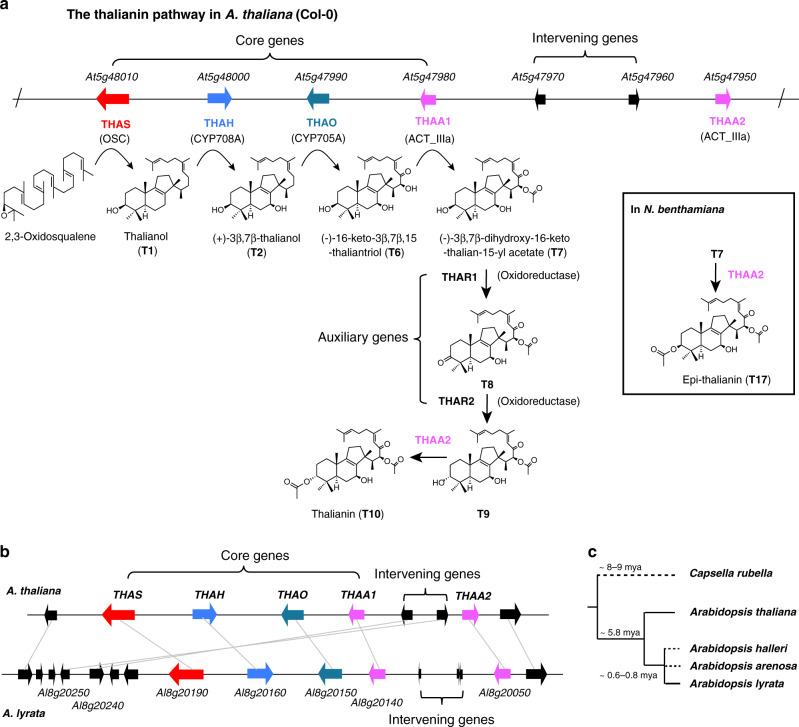
The emergence of the thalianol cluster in the Arabidopsis genus. **a** The characterized thalianin pathway in *A. thaliana* (modified from ref. ^14^). Genes are indicated by arrows: OSC, oxidosqualene cyclase; CYP, cytochrome P450; ACT_IIIa, acyltransferase subfamily IIIa. The compounds are numbered as in ref. ^14^. **b** Comparison of the thalianol cluster from *A. thaliana* and *A. lyrata*. Orthologous genes identified by reciprocal blast analysis between the two species are connected with gray lines. **c** Species tree indicating presence (solid line) or absence (dashed line) of the thalianol cluster. The times of divergence are from Hohmann et al.^12^.

In our original discovery of the thalianol BGC in *A. thaliana* accession Col-0 we reported four physically adjacent coexpressed genes (*THAS*, *THAH*, *THAO*, and *THAA1*) for the biosynthesis of thalianol-derived triterpenes^9^. Subsequent analysis revealed three additional genes (a linked gene *THAA2*, and two unlinked genes *THAR1* and *THAR2*) that are required for synthesis of the final pathway end product, thalianin (**T10**)^10^ (Fig. 1a). The original four core cluster genes are conserved between *A. thaliana* Col-0 and *A. lyrata*, both gene sets producing the acetylated thalianol derivative (-)-3β,7β-dihydroxy-16-keto-thalian-15-yl acetate (**T7**) when transiently expressed in *Nicotiana benthamiana*^10,11^ (Fig. 1a). While the order of the four core genes is the same in these sister species, the *THAA2* ortholog *AL8G20050* in *A. lyrata* has a different orientation (Fig. 1b). In addition, there are intervening genes between *THAA1* and *THAA2* in the two species, which are not implicated in plant metabolism and are not syntenic (Fig. 1b).

Thalianin belongs to the large and structurally diverse family of plant natural products known as triterpenes. In a previous study, we investigated the occurrence of triterpene BGCs in the sequenced genomes of 13 different Brassicaceae species^11^. We identified a superficially similar triterpene BGC in the genome of the related species *Capsella rubella*, which diverged from *A. thaliana* around 8–9 million years ago^12^ (Fig. 1c). This cluster transpired to be the result of an independent evolutionary event^11^. Other than the *C. rubella* example, thalianol-like clusters were not detected in any of the other species included in this analysis^11^. To further investigate the origins of the thalianol cluster, we next interrogated the newly generated genome sequence assemblies of two other *Arabidopsis* species, *Arabidopsis halleri*^13^ and *Arabidopsis arenosa* (this study; see Methods section). The first committed step in triterpene biosynthesis is the cyclization of 2,3-oxidosqualene to different triterpene scaffolds, a reaction carried out by enzymes known as oxidosqualene cyclases (OSCs). Systematic mining of the four *Arabidopsis* genomes identified a total of 55 predicted OSC genes (Supplementary Fig. 1). Phylogenetic analysis, in combination with investigation of the genomic locations of these OSC genes, revealed that the occurrence of bona fide syntenic thalianol synthase (*THAS*) genes was restricted to a monophyletic branch including only *A. thaliana* and *A. lyrata* OSC genes (marked in red in Supplementary Fig. 1). Consistent with this, analyses of the four *Arabidopsis* genomes using plantiSMASH analysis, a BGC mining algorithm designed for plants^14^, detected thalianol BGCs only in *A. thaliana* and *A. lyrata* (Supplementary Fig. 1). Thus, the thalianol cluster likely evolved in a common ancestor of the *Arabidopsis* genus around 5.8 MYA^12^ (Fig. 1c).

### Comparison of the thalianol cluster between species

**Fig. 2:**
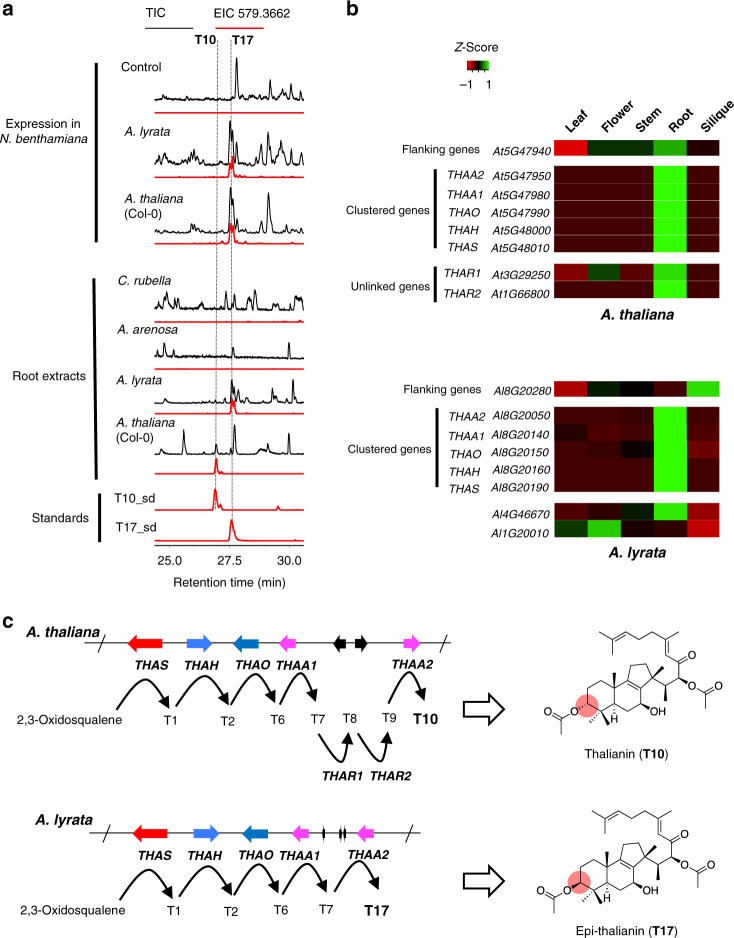
Involvement of auxiliary genes drives diversification of thalianol BGC between species. **a** LC-MS analysis of extracts from *Nicotiana benthamiana* leaves co-expressing thalianol pathway genes (top three traces) and of root extracts from *A. thaliana* accession Col-0 and *A. lyrata* accession VLH6. TIC, total ion chromatogram; EIC, extracted ion chromatogram. The mass fragment 579.3622 was used to detect **T10** and **T17**. The MS spectra underlying the EIC peaks are as shown in Supplementary Fig. 3b. Each experiment was independently repeated three times. **b** Comparison of the expression patterns of the thalianol cluster-related genes in different organs in *A. thaliana* and *A. lyrata*. Transcripts per kilobase million (TPM) values were used to generate the heatmap. The colors indicate the level of gene expression from low (red) to high (green). The *Z*-score represents the deviation from the mean by standard deviation units. **c** Divergence of the thalianol pathways in *A. thaliana* and *A. lyrata*. The black arrows show the directions of the biosynthetic pathways, starting from *THAS*. The numbering of the products is as in Fig. 1a. The structural differences between the final pathway end products **T10** and **T17** are highlighted. Source data underlying **b** are provided as a Source Data file.

Although the full thalianin pathway has been extensively studied in *A. thaliana* accession Col-0^9,10^, the full pathway in *A. lyrata* has not been characterized^11^. We therefore cloned the putative *THAA2* ortholog from *A. lyrata* (*AL8G20050;* 90.4% predicted protein identity to AtTHAA2). Coexpression of this gene with the four other *A. lyrata* thalianol cluster genes in *N. benthamiana* yielded epi-thalianin (**T17**) (Fig. 2a), indicating that these five clustered genes are functionally conserved between *A. thaliana* and *A. lyrata*. In line with this, all five genes are coexpressed in the roots in both *A. thaliana* and *A. lyrata* (Fig. 2b). In *A. thaliana*, two auxiliary oxidoreductase genes *THAR1* (*AT3G29250*) and *THAR2* (*AT1G66800*) are required for epimerization of the C3 hydroxy moiety of **T7** to give the final pathway end product, thalianin (**T10**)^10^ (Fig. 1a). Surprisingly, in *A. lyrata*, targeted metabolite analysis of root extracts did not identify **T10** but **T17** (Fig. 2a). The two isomers were not detectable in *C. rubella* and *A. arenosa*, both of which lack the thalianol BGC (Figs. 1c and 2a). Together our results indicate that the thalianol BGC has evolved specifically in the *Arabidopsis* genus, but is still dynamically evolving when *A. thaliana* and *A. lyrata* are compared. The presence of **T17** in *A. lyrata* root extracts further implies that the thalianol BGC in *A. lyrata* per se encodes a full biosynthetic pathway without the involvement of two auxiliary genes as in *A. thaliana*. In agreement with this, reciprocal BLASTP and synteny analysis were unable to identify putative orthologs of *THAR1* and *THAR2* in *A. lyrata*. Transcriptome analysis revealed that the most closely related gene to *A. thaliana THAR2* in *A. lyrata* (*AL1G20010*; 70.5% predicted protein identity) was preferentially expressed in the flowers rather than the roots, although the gene most closely related to *A. thaliana THAR1* (*AL4G46670*; 81.5% predicted protein identity) had a similar expression pattern to the thalianol cluster genes (Fig. 2b). In addition to being part of the thalianin pathway in *A. thaliana*, the *THAR2* gene is also required for the biosynthesis of arabidin, a triterpene derived from another *A. thaliana* Col-0 BGC^10,15^ (Supplementary Fig. 2a). However, the arabidin gene cluster is not present in *A. lyrata*^16^ (Supplementary Fig. 2b). Altogether, these results suggest that although the thalianol cluster genes share conserved expression patterns and enzymatic functions in *A. thaliana* and *A. lyrata*, in *A. lyrata* the five clustered genes encode a complete biosynthetic pathway for epi-thalianin (**T17**), while in *A. thaliana* the two additional unlinked genes *THAR1* and *THAR2* have been recruited to convert the **T7** pathway intermediate into thalianin (**T10**) (Fig. 2c).

### Variation in the thalianol cluster within species

**Fig. 3:**
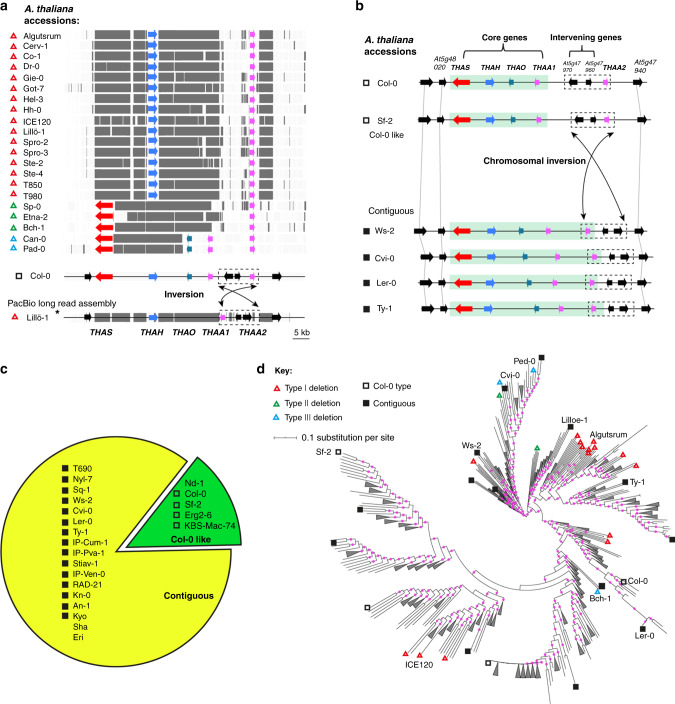
Variation in the thalianol cluster within *A. thaliana* species. **a** *A. thaliana* accessions identified from the *Arabidopsis* 1001 genome sequence database as lacking homologs to thalianol cluster genes (aligned with the Col-0 reference genome). Deletions are indicated by gray bars. The three different types of deletion patterns (lacking one or more cluster genes) are indicated by colored triangles. A fully assembled region from long-read re-sequencing of the accession Lillö-1 (asterisked) showing an inversion in this region is illustrated at the bottom. **b** Chromosomal inversions in the thalianol region from genomes of representative de novo assembled *A. thaliana* accessions. **c** Frequencies of Col-0-like and contiguous thalianol clusters in the genomes of 21 de novo assembled *A. thaliana* accessions. Accessions Sha and Eri are newly PacBio-sequenced accessions that were not included in the 1135 short read sequenced collections^17^. **d** Phylogenetic tree for 1135 *A. thaliana* accessions. Phylogenetic distances were calculated based on 28,775 filtered SNPs using RAxML^40^. Tree node support (>90%) is indicated by pink dots on branches (1000 bootstrap replicates). Accessions with thalianol pathway gene deletions and chromosomal inversion variations are indicated. Other monophyletic groups are collapsed.

We next systematically examined polymorphisms, including SNPs, small indels and large gene deletion variations, in the thalianol cluster genes based on genome sequences generated with short reads from 1135 *A. thaliana* accessions^17^ (https://tools.1001genomes.org/polymorph/). Overall, SNPs in the thalianol cluster genes were the most common types of polymorphisms (97%), followed by small indels (55% and 36% for insertions and deletions, respectively) (Supplementary Table 1). These polymorphisms were mostly benign, with only two accessions having SNPs that are predicted to have a large effect (i.e., premature stop codons) on the *THAS* gene and most accessions having small indels in the UTR regions of the cluster genes (Supplementary Table 1). Only ~2% (21/1135) of accessions were found to have large gene deletions involving one or more cluster genes. Of those, 16 accessions lacked the *THAS*, *THAO*, and *THAA1* genes and a large 3′-UTR region of the *THAA2* gene (~6 kb) (Fig. 3a). Other types of deletions were also found at much lower frequencies (Fig. 3a). The two unlinked genes (*THAR1* and *THAR2*) are present in nearly all of the *A. thaliana* accessions (Supplementary Fig. 3a). In accordance with this, analysis of transcriptome data for seven *A. thaliana* accessions revealed similar expression patterns for the thalianol cluster genes as in Col-0^9^ (Supplementary Fig. 3b). Metabolite analysis confirmed that **T10** could be detected in root extracts from accessions that had the thalianol cluster genes but not in those that lacked them (e.g., Cerv-1, Dr-0, and Lillö-1) (Supplementary Fig. 4). Taken together, we conclude that this cluster is likely to be functional in the vast majority of *A. thaliana* accessions.

To gain insights into polymorphisms in gene order for the thalianol BGC, we analyzed 22 *A. thaliana* genome sequence assemblies generated with PacBio long reads (see Methods section). These analyses confirmed the deletion profile observed for the Lillö-1 accession (Fig. 3a). They further showed that, while the order of the four core genes is well conserved, that of the intervening genes between *THAA1* and *THAA2* is not. Of the 21 assembled *A. thaliana* genomes (excluded Lillö-1, since it lacked most of the core genes), only three had the same gene order as the Col-0 thalianol cluster. Strikingly, in the remaining 17 accessions the *THAS, THAH, THAO, THAA1*, and *THAA2* genes were contiguous, with no predicted genes between *THAA1* and *THAA2*. The reversed orientation of the three rearranged genes and detailed alignment analysis suggests that these natural accessions have experienced chromosomal inversions to form compact thalianol clusters relatively recently (Fig. 3b, c and Supplementary Fig. 5). Although our sample size for de novo sequenced genomes is relatively small (21 out of 1135 accessions), the contiguous forms of the thalianol cluster constitute the majority (17 out of 22; 77%) of these accessions and are phylogenetically widely distributed (Fig. 3d). We hypothesize that chromosomal inversion may facilitate the compaction of the thalianol BGC in natural populations.

### Analysis of selection on the thalianol pathway

**Fig. 4:**
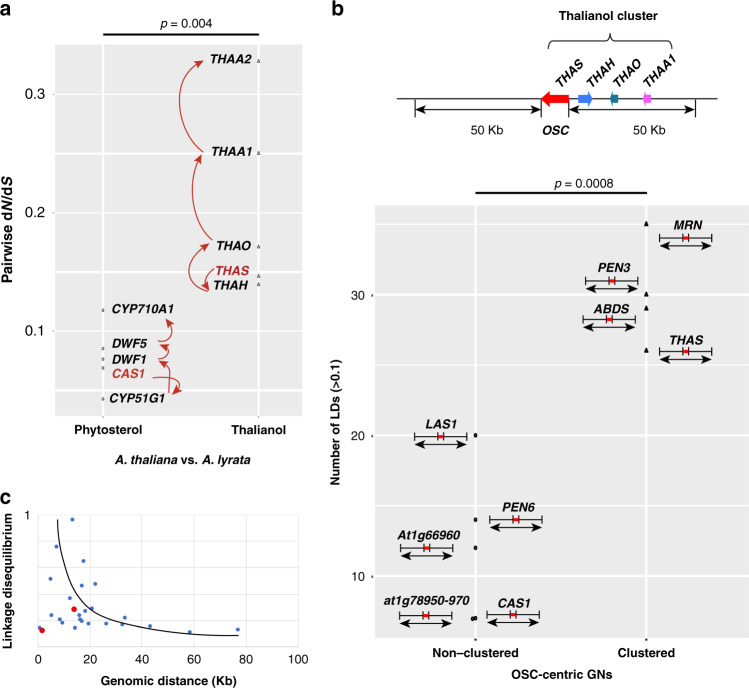
Molecular evolution analysis within and between species. **a** Pairwise d*N*/d*S* analysis between *A. thaliana* and *A. lyrata* for the thalianol pathway genes. The phytosterol pathway genes were used as controls for strong purifying selection. The arrows illustrate the directions of the biosynthetic pathways, starting with cycloartenol synthase (*CAS1*) for sterol biosynthesis and *THAS* for thalianol-derived triterpenes. Two-sided Student *t*-test, df = 8. **b** Comparison of LD (*r*^2^ > 0.1) numbers simulated from the *A. thaliana* 1001 genomes SNP data across genome neighborhoods for clustered and non-clustered OSC genes. Genome neighborhoods were defined as 50 Kb either side of each OSC gene. Although the *At1g78500* gene product PEN6 is phylogenetically close to the four OSCs that each form BGCs^9^ (Supplementary Fig. 1), analysis of the expression patterns of the genes neighboring *At1g78500* indicate that it is unlikely to form part of a functional BGC (Supplementary Fig. 7). Therefore, the *PEN6*-centric genomic neighborhood (GN) was considered as non-clustered. Two-sided Student *t* test, df = 7. **c** Plot of LD values (*r*^2^ > 0.1) in the *THAS*-centric GN along with the genomic distances of biallelic SNPs. The two LDs located in the region encompassing the four cluster genes shown in **b** are marked in red. The black line is a smoothed trend fitted by local regression (LOESS).

To investigate what types of selection may shape the within and between species variations of the thalianol BGC, we simulated variant data at within-species and/or between-species levels. Pairwise between-species nonsynonymous/synonymous substitution (d*N*/d*S*) analysis identified relaxed purifying selection for the thalianol cluster genes when compared with phytosterol pathway genes (which are highly conserved across land plants), reflecting the *Arabidopsis-*genus specific distribution of this gene cluster (Fig. 4a). Notably, the later thalianol pathway genes were far less constrained than the early pathway genes (Supplementary Fig. 6). This is consistent with different roles for THAA2 in *A. thaliana* and *A. lyrata* as shown in Fig. 2c. Branch-site model analysis (see Methods section) did not detect gene-wide positive selection for any of the thalianol cluster genes (Supplementary Table 2), although a site model analysis (see Methods section) did detect episodic positive selection on several sites across the clustered genes (Supplementary Table 3). These analyses are in agreement with the conserved enzymatic functions of the thalianol pathway genes between species. We next performed several versions of the McDonald–Kreitman (MK) test^18^ by using both polymorphism and divergence to test for positive selection in more recent evolutionary time (see Methods section). These analyses found no signature of current positive selection imposed on the thalianol cluster genes (Supplementary Table 4). Altogether, our results suggest that individual genes for thalianol BGC are mostly under purifying selection, presumably due to their conserved enzymatic functions and expression patterns within and between species.

Having carried out evolutionary tests on single genes, we then expanded our tests for positive selection to consider the thalianol cluster as a whole by performing genome-wide linkage disequilibrium (LD) analysis using SNP data from the 1135 *A. thaliana* genomes. The genome of *A. thaliana* accession Col-0 contains 13 OSC genes, each with different functions^9^. These genes are located in a total of nine genomic neighborhoods (GNs), tandem duplicated OSC genes being treated as one GN (see Methods section). We systematically examined the LD extensions of these nine OSC GNs. To do this, we counted the number of squared allele frequency correlation (*r*^2^) values (*r*^2^ > 0.1) for biallelic sites located within the OSC-centric GNs (spanning 50 Kb on each side of an OSC locus; see Fig. 4b). Our results demonstrate that GNs bearing OSC gene clusters have significantly more nonrandom associations of biallelic sites than GNs containing OSC genes that are not part of BGCs (Student *t*-test; Fig. 4b). In addition, nonrandom associations of biallelic sites in the *THAS*-centric GN are mostly located in the flanking regions of the thalianol cluster and decay with increasing genetic distance (Fig. 4c), only two being detected in the region encompassing the four cluster genes shown in Fig. 4c (indicated by the red circles). This pattern suggests that the thalianol cluster is currently undergoing or has recently undergone a selective sweep, which has reduced the diversity in the cluster region. The elevated LDs in the flanking regions are presumably due to the hitch-hiking effect^19^. This interpretation is further supported by the low levels of single feature polymorphism (SFPs) across the thalianol core cluster in comparison to the flanking regions (Supplementary Fig. 8), suggesting that the thalianol cluster as a whole is under directional positive selection.

## Discussion

**Fig. 5:**
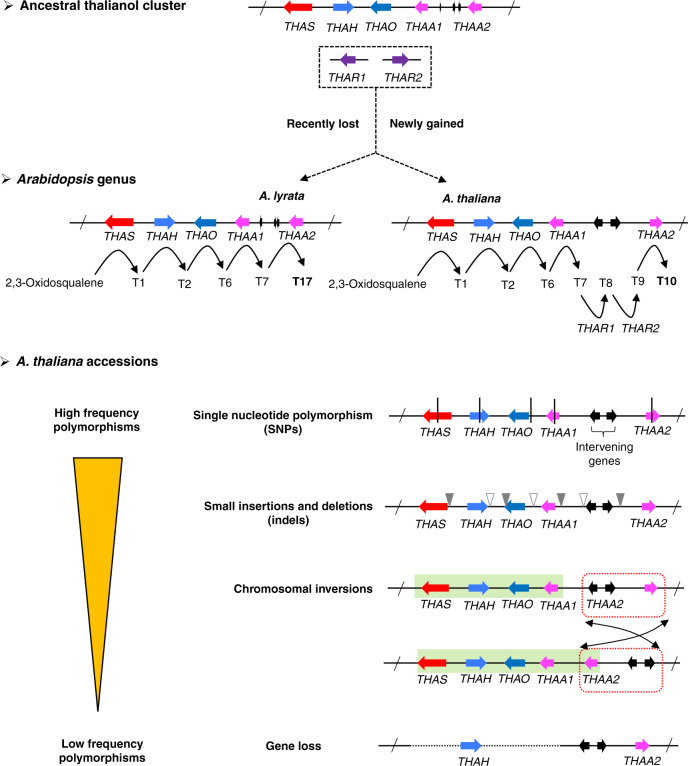
Schematic illustrating the cluster variants and polymorphisms identified for the *Arabidopsis thalianol* cluster in this study. The ancestral state for the two unlinked genes *THAR1* and *THAR2* is ambiguous based on the current data. The divergence (between species) and polymorphisms (within species) for the thalianol cluster are shown.

Here, we have carried out large-scale investigation of the evolutionary dynamics of a plant BGC, the thalianol cluster. Our study suggests that an ancestral thalianol cluster evolved relatively recently in evolutionary time, before the split of *A. thaliana* and *A. lyrata*. The cluster was not detected in the *A. halleri* and *A. arenosa* accessions included in our study. Analysis of additional accessions as more genome sequences become available will establish whether lack of the thalianol BGC is a consistent feature of these two species. Comparisons of the thalianol clusters in *A. thaliana* and *A. lyrata* reveal differences in cluster organization and function. In *A. lyrata*, five clustered genes are necessary and sufficient for biosynthesis of the pathway end-product epi-thalianin (**T17**), while in *A. thaliana* a further two unlinked genes (*THAR1* and *THAR2*) are required to give the corresponding pathway end-product, thalianin (**T10**) (Fig. 5). Thus the interplay between the core BGC and unlinked auxiliary genes may provide a mechanism for diversification of BGCs between plant species.

Future experiments will establish whether epi-thalianin contributes to the establishment of microbial communities in and around *A. lyrata* roots, and if so how distinct these communities are from those of *A. thaliana*^10^.

SNPs and genomic deletion/insertion events have been implicated in the maintenance and diversification of BGCs in fungi, along with horizontal gene transfer from taxonomically distant fungal species^20^. Our investigations of the genomes of over a thousand *A. thaliana* accessions from around the world reveal that SNPs and small indels are the most common sequence polymorphisms in the thalianol cluster genes, followed by chromosomal inversions and gene loss (Fig. 5). Overall, the frequency of predicted deleterious mutations was low, suggesting that the thalianol cluster is fixed and functional in the vast majority of accessions.

**Fig. 6:**
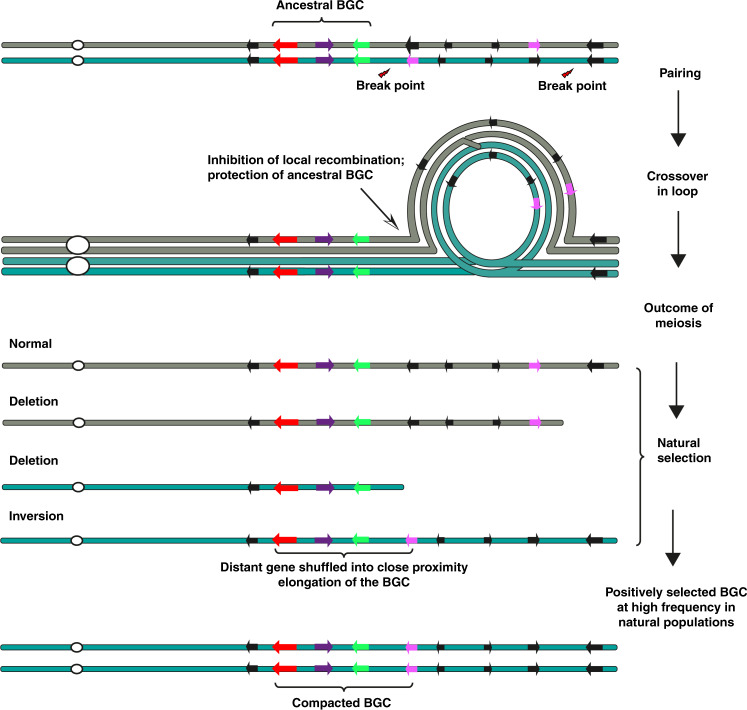
A proposed model for compaction/assembly of plant BGCs through chromosomal inversions. Schematic inspired by Wellenreuther and Bernatchez^23^. Genes encoding enzymes are indicated with colored arrows.

Our results provide evidence to suggest that chromosomal inversions may lead to cluster compaction (i.e., by bringing the *THAA2* gene into the core thalianol cluster) (Fig. 3b, c). Although the role of chromosomal inversions in evolution in animals has been extensively investigated^21,22^, examples of such studies in plants are few^23^. Chromosomal inversions suppress recombination within and around the inversion region, and so may insulate clustered coadapted alleles against dispersal^24^ (Fig. 6). Indeed, our systematic LD analysis identified significantly more coinherited biallelic SNPs in the genomic neighborhoods (GNs) of OSC genes that form parts of BGCs compared to those of nonclustered OSCs (Fig. 4b). In line with this, a recent population genomic study also identified evolutionarily recent inversions associated with adapted quantitative trait loci (QTLs) in Drummond’s rockcress (*Boechera stricta*)^25^. The high frequency of compact thalianol BGCs in natural *A. thaliana* populations (Fig. 3b–d) is thus in accordance with the signatures of positive selection detected on the thalianol BGC as a whole, suggesting that the chromosomal inversions that we have identified are more likely to have driven cluster assembly rather than cluster break-up. Closer physical colocalization of genes will reduce the likelihood of loss of single pathway genes during recombination^2,26^. Altogether, our results suggest that chromosomal inversion may be a genetic mechanism enabling growth and compaction of BGCs in plants.

The reasons for the existence of BGCs in eukaryotic genomes remain hotly debated. Several scenarios, i.e., coinheritance, coregulation, and avoidance of toxic effects from pathway intermediates have been proposed^2^. These various hypotheses are not mutually exclusive. The overriding point, however, is that the formation and maintenance of BGCs must inevitably be a consequence of natural selection. Analysis within and between species for the thalianol BGC suggest that both positive and purifying selections are likely involved in shaping the formation and maintenance of this cluster (Figs. 3c and 4). These antagonistic selection pressures may further reduce the local recombination between physically linked enzymatic genes^26^. As we learn more about the dynamics of BGCs within and between species, we will be able to gain further insights into the forces and mechanisms shaping genome architecture and adaptive evolution.

## Methods

### Plant materials and growth conditions

Seeds of *A. thaliana* accessions were obtained from Professor Jian Hua (Cornell University) and NASC (http://arabidopsis.info/). Seeds of *A. arenosa* were obtained from Prof. Kirsten Bomblies (ETH, Zurich). All seeds were surface sterilized in 5% sodium hypochlorite for 7 min, followed by incubation in 70% ethanol for 5 min, and then washed by ddH_2_O for five times. The seeds were placed on ¼ MS medium, subjected to cold treatment (4 °C, 3 days) before moving to long-day conditions (22 °C; 16 h light/8 h dark). For *A. lyrata*, 3 weeks of cold treatment were applied. *N. benthamiana* plants were grown in a greenhouse under long-day conditions (22 °C; 16 h light/8 h dark).

### Genome sequencing and assembly of *A. arenosa*

We used 0.4 g of *A. arenosa* leaf material from the accession SNO (Strecno, Slovakia, 49.17417N, 18.86167E). The frozen leaf material was ground in liquid nitrogen, and then 10 mL of CTAB DNA extraction buffer was added (Tris-HCl 100 mM (Fisher Scientific), CTAB 2% (w/v) (Sigma), NaCl 1.4 M (Sigma), EDTA 20 mM (Sigma-Aldrich)) and 20 μL of Proteinase K at 20 mg/mL (Qiagen). The mixture was incubated at 55 °C for 1 h then cooled on ice. Chloroform (0.5× volume; Fisher Scientific) was added and inverted to mix. Material was centrifuged at 1008×*g* for 30 min, and the upper phase recovered. Then 1× volume of phenol:chloroform:isoamyl alcohol (25:24:1; Sigma) was added and the mixture centrifuged for 30 min at 1008×*g*. The upper phase was recovered and 10% volume NaOAc at 3 M (Sigma) was added along with 2.5× volume of ice cold 100% ethanol (Sigma-Aldrich). The tubes were inverted to mix and incubated on ice for 30 min before centrifugation for 30 min at 1008×*g* (4 °C). The pellets were washed in 4 mL of ice cold 70% ethanol (Sigma-Aldrich). Tubes were spun for 10 min at 1008×*g* at 4 °C and the 70% ethanol wash repeated twice more. The pellets were air dried and resuspended in 300 μL nuclease-free water (Qiagen) with 3 μL RNase A at 4 mg/mL (Qiagen). DNA concentrations were determined on a Qubit Fluorometer 2.0 (Invitrogen) using the Qubit dsDNA HS Assay kit. Fragment sizes were assessed using a Q-card (OpGen Argus) and the Genomic DNA TapeStation assay (Agilent).

The DNA was diluted to 0.5 ng/μL with EB (Qiagen) and checked with a Qubit Fluorometer 2.0 (Invitrogen) using the Qubit dsDNA HS Assay kit. The Chromium User Guide was followed as per the manufacturer’s instructions (10× Genomics, CG00043, Rev A). The final library was quantified using qPCR (KAPA Library Quant kit, Illumina; ABI Prism qPCR Mix, Kapa Biosystems). The sizes of the library fragments were monitored using a Bioanalyzer (High Sensitivity DNA Reagents, Agilent). Samples were pooled based on the molarities calculated using the two QC measurements.

Sample SNO was sequenced on an Illumina HiSeq2500, using Rapid Run V2 mode150 PE, generating 82.10 Mb reads. These were assembled using Supernova 2.0.0^27^, giving a raw coverage of 57.91× and effective coverage of 45.30×. The molecule length was 26.58 Kb.

The assembly scaffold N50 was 2.19 Mb and overall size (counting only scaffolds longer than 10 Kb) was 127.02 Mb. Gene content was estimated using BUSCO v2^28^ by searching database embryophyte_odb9. This showed that the gene space of the *A. arenosa* assembly was nearly complete, with 97.5% of the plant-specific BUSCOs present and 1.4% missing. Of those present, 4.7% were duplicate copies (Supplementary Table 5).

### RNA and cDNA preparation

*A. lyrata* plants postflowering were used for total RNA isolation. Roots were recovered from soil, washed thoroughly with sterile water and dried with a clean tissue. Around 10 mg of ground roots were extracted with 600 μL of TRIzol reagent (ThermoFisher, Carlsbad, CA, USA). Chloroform (120 μL) was added to remove proteins and other impurities. After centrifugation (13,000×*g*; 10 min), an aliquot (400 µL) of supernatant was recovered and an equal volume of isopropanol added. Following mixing the pellet was recovered by centrifugation (10,000×*g*; 10 min) and washed with 70% ethanol. After centrifugation (10,000×*g*; 5 min) the pellet was dried at room temperature for 10 min. Sterilized water (30 μL) was then added to dissolve the total RNA. Reverse transcription reactions were performed using ≤2 μg of total RNA, random primers and a reverse transcription kit (Agilent, Santa Clara, CA, USA). The cDNA library was then used for amplification of the coding sequence of *AlTHAA2* (*AL8G20050*). A pair of gene-specific primers attached with GATEWAY *att* sites were used (Fwd: GGGGACAAGTTTGTACAAAAAAGCAGGCTTCATGGACACCATGAAGGTTGAAATC; Rev: GGGGACCACTTTGTACAAGAAAGCTGGGTTTTAGATCAATACACTTGGATTTG). cDNAs were cloned into the GATEWAY entry vector pDONR207 (Invitrogen).

### Transient plant expression

The pEAQ-Dest-1 expression vector was used for transient plant expressing experiments^29^. Constructs for transient expression of other *A. lyrata* cluster genes and *A. thaliana* thalianol cluster genes were adopted from previous work^10,11^. All constructs were verified by sequencing and introduced into *A. tumefaciens* strain LBA4404. Agro-infiltration of *N. benthamiana* was carried out as described previously^30^. *N. benthamiana* leaves were harvested 6 days after infiltration.

### LC-MS-IT-TOF analysis

Ten mg of freeze-dried *N. benthamiana* leaves were homogenized with a tungsten bead (3 mm, Qiagen) and extracted with 200 µL of pure methanol (sonication 30 min). The extracts were centrifuged at 16,000×*g* for 1 min. Aliquot of (170 µL) supernatant was recovered and filtered through 0.45 µm PTFE spin columns before injection onto LC. For root metabolites profiling, ten-days roots grown on vertical ¼ MS plates were harvested and flash frozen with liquid nitrogen. Samples were then freeze-dried. For comparison, 10 mg of sample were weighed out and extracted as described for tobacco samples. LC-MS analysis was carried out on a Prominence/Nexera UPLC system attached to an Ion-trap Tof mass spectrometer (Shimadzu). Separation was performed on a 100 × 2.1 mm Kinetex reverse column (1.7 µm C18 100 Å), using the following gradient of acetonitrile (solvent B) versus 0.1% formic acid in miliQ water (solvent A): 0.5 min, 2% B; 5 min, 10% B; 17 min, 30% B; 33 min, 90% B; 35.8 min, 100% B; 43 min, 100% B; 45 min 2% B. The flow rate was 0.5 mL/min and the run temperature was set at 40 °C. Injection volume was 1 µL. The MS data were acquired in both positive and negative modes. Spectra were collected from *m*/*z* 200–2000 with automatic sensitivity control set to a target of 70% optimal base peak intensity. The instrument was calibrated immediately before analysis using sodium trifluoroacetate cluster ions, according to the manufacturer’s instructions. The standards (**T10** and **T17**) were generated in previous work^10^.

### In silico analysis of publicly available transcriptome data

RNA-seq data (SRA files) for five tissues (root, leaf, stem, flower, and silique) from *A. thaliana* (Col-0) and *A. lyrata* were downloaded from the National Center for Biotechnology Information (NCBI) BioProject PRJNA336053^31^. RNA sequence data for roots and shoots for *A. thaliana* accessions Col-0, Bay-0, Cvi-0, Ita-0, Kas-1, LP2-6, and Ws-2 were downloaded from NCBI BioProject PRJEB14092^32^. The sequencing quality was checked by FastQC v0.10.1^33^. Trimmomatic v0.39^34^ was used to trim adapters and overrepresented sequences. Sequence data were then aligned to reference genomes (PHYTOZOME v.12.0; https://phytozome.jgi.doe.gov/pz/portal.html) by HISAT2 v2.1.0^35^. Abundance was called using StringTie v1.3.5^36^. The transcripts per million (TPM) values were then extracted as expression levels for genes of interest.

### Genome mining and plantiSMASH analysis

The Arabidopsis genome sequences (*A. thaliana* accession Col-0, *A. lyrata*, *A. halleri*) were retrieved from PHYTOZOME v.12.0. The genome of *A. arenosa* was generated de novo as part of this work. OSC genes were identified using HMMER3^37^ with HMMER profiles (pHMMs) PF13243 and PF13249 downloaded from the PFAM library^38^. The cut_tc (trusted cut-off) option was used. Pseudogenes for OSCs (predicted protein sequence < 600 amino acids) were discarded from the analysis. The HMMER output was aligned using MUSCLE^39^. The alignment was used to infer the maximum likelihood tree by RaxML^40^. The OSC tree topology was consistent with a previous study that included OSCs from the wider Brassicaceae^11^. For plantiSMASH analysis^14^, the genome files (FASTA format) and annotation files (GFF3 format) were converted to GenBank format as input files.

### Long read assemblies for the thalianol cluster genomic region

Long read genome sequences for the thalianol cluster region from 22 *A. thaliana* accessions were retrieved from the 1001 Genomes Plus project. Seven used in this study (Nd-1, An-1, Cvi-0, Eri-1, Kyo, Ler-0, and Sha) were previously published^41,42^.

### Pair-wise d*N*/d*S* analysis

The pairwise d*N*/d*S* (*ω*) analysis averages the d*N*/d*S* ratio across all sites. To determine the estimates of *ω*, pairwise CDS (coding sequence) alignments of *A. thaliana* accession Col-0 and *A. lyrata* for the thalianol cluster genes and phytosterol pathway genes^43^ were used as an input for the codeml program from PAML 4.9^44^. To determine if the d*N*/d*S* ratio was significantly different from 1, the program was executed twice with different control files. To obtain the maximum likelihood estimates of *ω*, the control file was set as: runmode = −2, model = 0, NSsites = 0, Fix_omega = 0. To obtain the likelihood with *ω* = 1, the control file was set as: runmode = −2, model = 0, NSsites = 0, Fix_omega = 1, Omega =1. The log likelihood values from the two executions were subtracted. The negative of twice of this value was used for likelihood ratio test (LR test). The *p* value was determined by comparison to *χ*^2^ with one degree of freedom. The estimated ω values with *p* value < 0.001 were plotted.

### Codon-based positive selection analysis

The protein-coding DNA sequences were aligned with TranslatorX^45^. In order to minimize negative impacts due to sequence size, gene sequences less than (for *THAS* min = 2200 bp; for *THAO* min = 1450 bp; for *THAH* min = 1400 bp; for *THAA* min = 1200 bp) were not included for the analysis. The sequences were aligned by MUSCLE^39^. Poorly aligned regions in the alignment were removed by Gblocks^46^. The Gblocks filtered nucleotide sequence were further translated to protein sequence and used to infer phylogenetic tree. The final Gblocks nucleotide sequence alignment and the protein phylogenetic tree were used for various evolutionary tests using HyPhy^47^. The input file for HyPhy analysis is shown in Supplementary Data 1. BUSTED analysis (similar to “branch-site” model in PAML) was used to infer whether a gene has experienced positive selection at at least one site on at least one branch^48^. MEME analysis (similar to “site” model in PAML) was used to detect individual sites evolving under positive selection in a proportion of branches (labeled as {foreground} in the input file)^49^. For both analyses, “universal genetic code” were selected and *p* < 0.05 was set for significance.

### Tests for neutral evolution

McDonald and Kreitman analysis (MK test) is less sensitive to demographic factors such as migration, making it useful for detecting selection^18^. It captures infrequent adaptive mutations which fix fast relatively to regular neutral mutations, therefore elevating the divergence ratio (between species) over the polymorphism ratio (within species), a signature of positive selection. To examine departures from the neutral theory of molecular evolution, MK test was performed on a publicly available web interface (iMKT) (https://imkt.uab.cat/)^50^. The input sequences for polymorphism were downloaded from the 1001 *Arabidopsis* genome browser (http://signal.salk.edu/atg1001/3.0/gebrowser.php). The corresponding outgroup gene sequences from *A. lyrata* were taken from either^11^ for the thalianol core cluster genes or retrieved via reciprocal blast of the Col-0 reference gene on the Phytozome website (https://phytozome.jgi.doe.gov/pz/portal.html) for *THAA2*. Several versions of the MK test were performed to compute the polymorphism data from 1001 accession genomes and the outputs were compared with divergence data between *A. thaliana* and *A. lyrata*. The standard MK test can detect positive selection if adaptive mutations rapidly reach fixation and thus contribute relative more to divergence than to polymorphism when compared to neutral mutations. In this case, a positive alpha (*α*) indicates the proportion of nonsynonymous substitutions that have been fixed by positive selection. To avoid bias from the segregation of slightly deleterious nonsynonymous substitutions, we also applied Fay, Wycoff, and Wu (FWW) correction^51^ on the standard MKT by considering only those polymorphic sites with a frequency above the cutoff of 0.05. Sometimes when relaxed purifying selection reaches neutral, it can cause an increased level of polymorphism relative to divergence, therefore adaptive estimation (*α*) will be underestimated. By taking account of bias from both segregation of weakly deleterious variants and relaxed purifying selection, we further applied Extended MKT^52^ to the thalianol pathway genes.

### Construction of a phylogenetic tree for *A. thaliana* accessions using SNP data

The 1001_SNP_MATRIX was downloaded from the 1001 genomes data center (https://1001genomes.org/data/GMI-MPI/releases/v3.1/SNP_matrix_imputed_hdf5/).

SNPs with a minor allele frequency (MAF) of 0.05 or greater (28,775 variants) were then computed by SNPhylo^53^ to generate a maximum likelihood tree.

### Linkage disequilibrium analysis

The 1001_SNP_MATRIX was used to simulate the genome-wide linkage disequilibrium (squared allele frequency correlation, *r*^2^) using TASSEL 5^54^. Only the top 50 *r*^2^ values are reported. The *p*-values for biallelic sites were calculated using Fisher’s exact test in Tassel. *r*^2^ = 0.1 was used as the threshold. *r*^2^ values from biallelic sites that are both located within the defined *OSC* gene-linked genomic regions (50 kb before and after the coding region of an *OSC* gene) were extracted. There are 13 *OSC* loci in the *A. thaliana* Col-0 genome^9^. Five *OSC* genes were located in clustered *OSC* gene-centric genomic regions: *THAS* (*At5g48010*)^9^, *MRN* (*At5g42600*)^55^, *PEN3* (*At5g36150*)^56^, and *ABDS* (*At4g15340*, *At4g15370*)^15^. The nonclustered *OSC* genes^11^ were: *LAS1* (*At3g45130*), *PEN6* (*At1g78500*), *At1g66960*, *At1g78950-970* (*At1g78950, At1g78955, At1g78960, At1g78970*), and *CAS1* (*At2g07050*). The tandem duplicates in the *At1g78950-970* GN were considered as a single locus, so the 50 kb on the left was taken before *At1g78950* and the 50 kb on the right was taken after *At1g78970* gene.

### Reporting summary

Further information on research design is available in the Nature Research Reporting Summary linked to this article.

## Supplementary information

Supplementary Information

Reporting Summary

Description of Additional Supplementary Files

Supplementary Data 1

## Data Availability

Data supporting the findings of this work are available within the paper and its Supplementary Information files. The datasets and plant materials generated and analyzed during the current study are available from the corresponding author upon request. The sequence data for *A. arenosa* have been deposited in the European Nucleotide Archive with the project ID PRJEB37828. The sequences for the 22 thalianol cluster de novo assemblies were deposited to NCBI. The accession numbers are listed in Supplementary Table 6. MK test was performed on a publicly available web interface (https://imkt.uab.cat/). The input sequences for polymorphism were downloaded from the 1001 *Arabidopsis* genome browser (http://signal.salk.edu/atg1001/3.0/gebrowser.php). Source data are provided with this paper.

## Source data

Source Data
