# Supplementary material for: Formation and diversification of a paradigm biosynthetic gene cluster in plants
Source: Nat Commun. 2020 Oct 23;11:5354. doi: 10.1038/s41467-020-19153-6 (PMC7584637; doi:10.1038/s41467-020-19153-6)
Supplement: Supplementary file 1 — Supplementary Information [file 41467_2020_19153_MOESM1_ESM.pdf]

Formation and diversification of a paradigm biosynthetic gene cluster in plants

Liu *et al.*

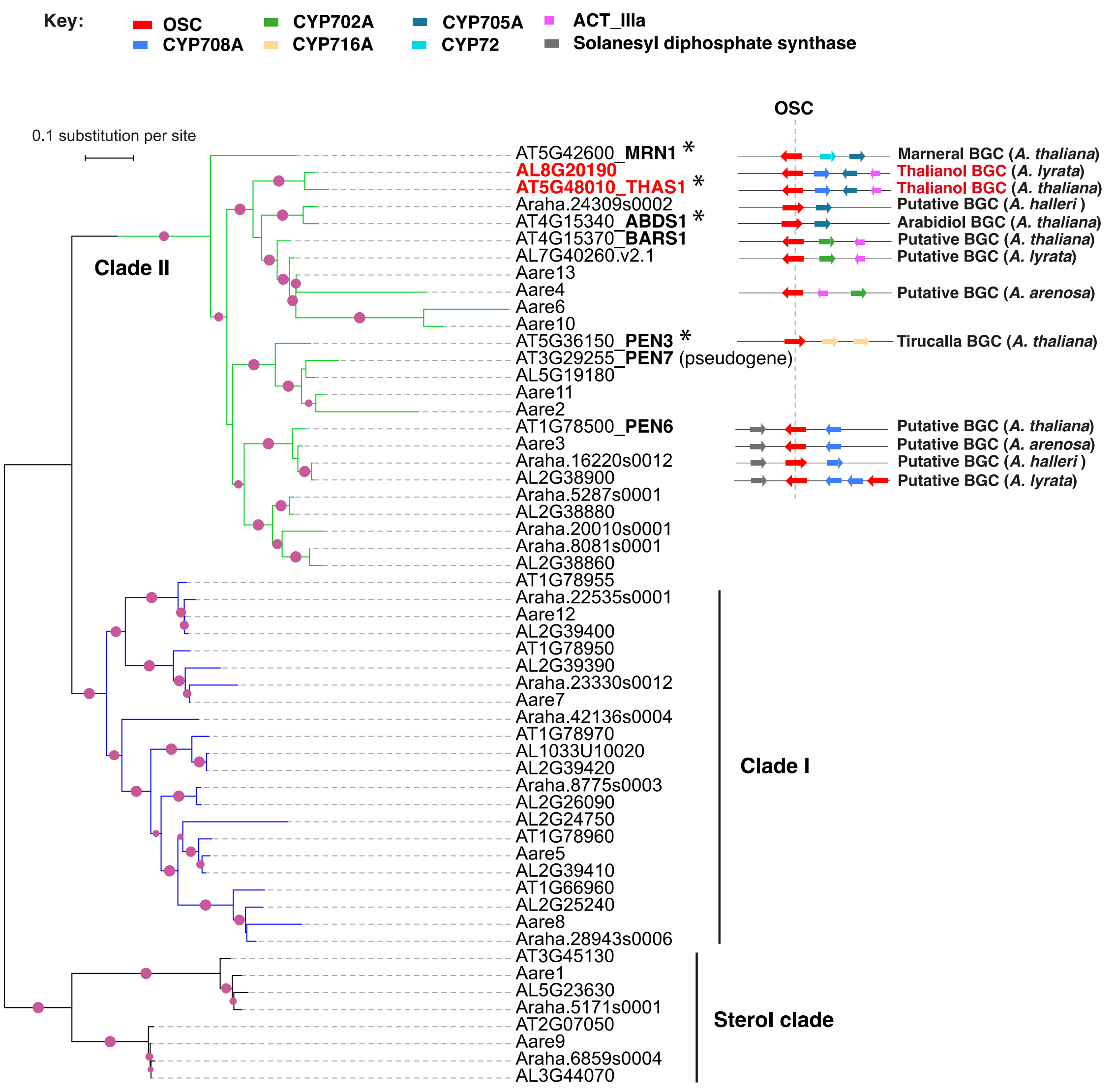

Supplementary Figure 1. Genome mining of OSCs in four Arabidopsis species and scanning for triterpene gene clusters with plantiSMASH. Maximum likelihood tree of OSC proteins from *A. thaliana*, *A. lyrata*, *A. halleri* and *A. arenosa*. The tree topology (sterol clade, clades I and II) is in accordance with previous studies [1,2]. Pink circles indicate tree node support (>70%; 1000 bootstrap iterations). Enzyme families are indicated by colors (see key). PlantiSMASH analysis [3] identified 5, 3, 2 and 2 triterpene BGCs in *A. thaliana*, *A. lyrata*, *A. halleri* and *A. arenosa* genomes, respectively. Notably, the *ABDS1* and *BARS1* genes are in the same genomic neighborhood and are identified as a single BGC by plantiSMASH. *, characterized BGCs in *A. thaliana* (Col-0) [4].

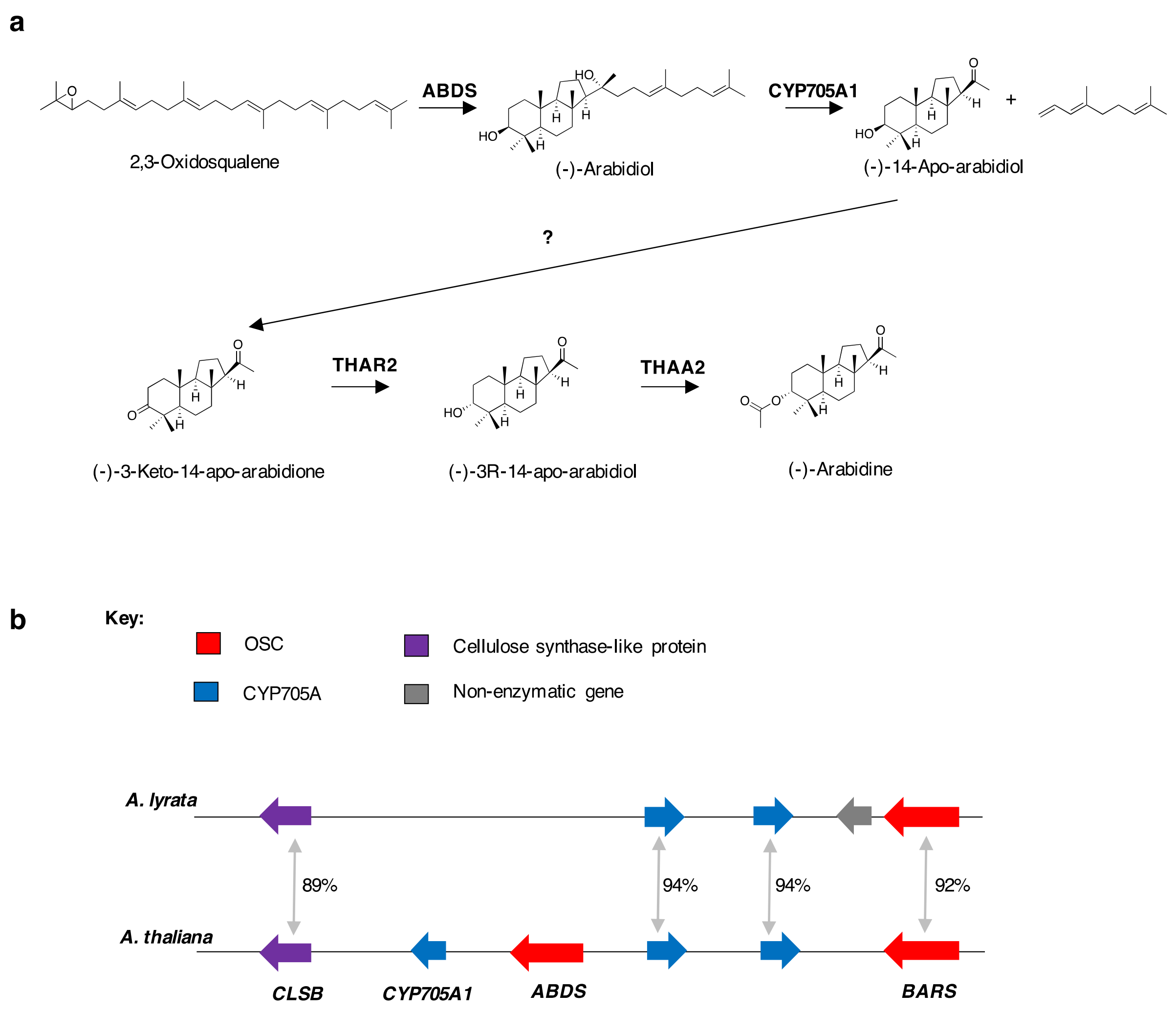

Supplementary Figure 2. ***A. thaliana*** **arabidin pathway and syntenic comparison of the** ***ABDS-CYP705A1*** **genomic neighborhood between** ***A. thaliana*** **and** ***A. lyrata*****. a**, The arabidin pathway [5,6]. **b**, Comparison of the *A. thaliana* Col-0 arabidin cluster region with the syntenic region of *A. lyrata*. Amino acid sequence identity between orthologous genes is shown. Enzyme families are indicated by colors (see key).

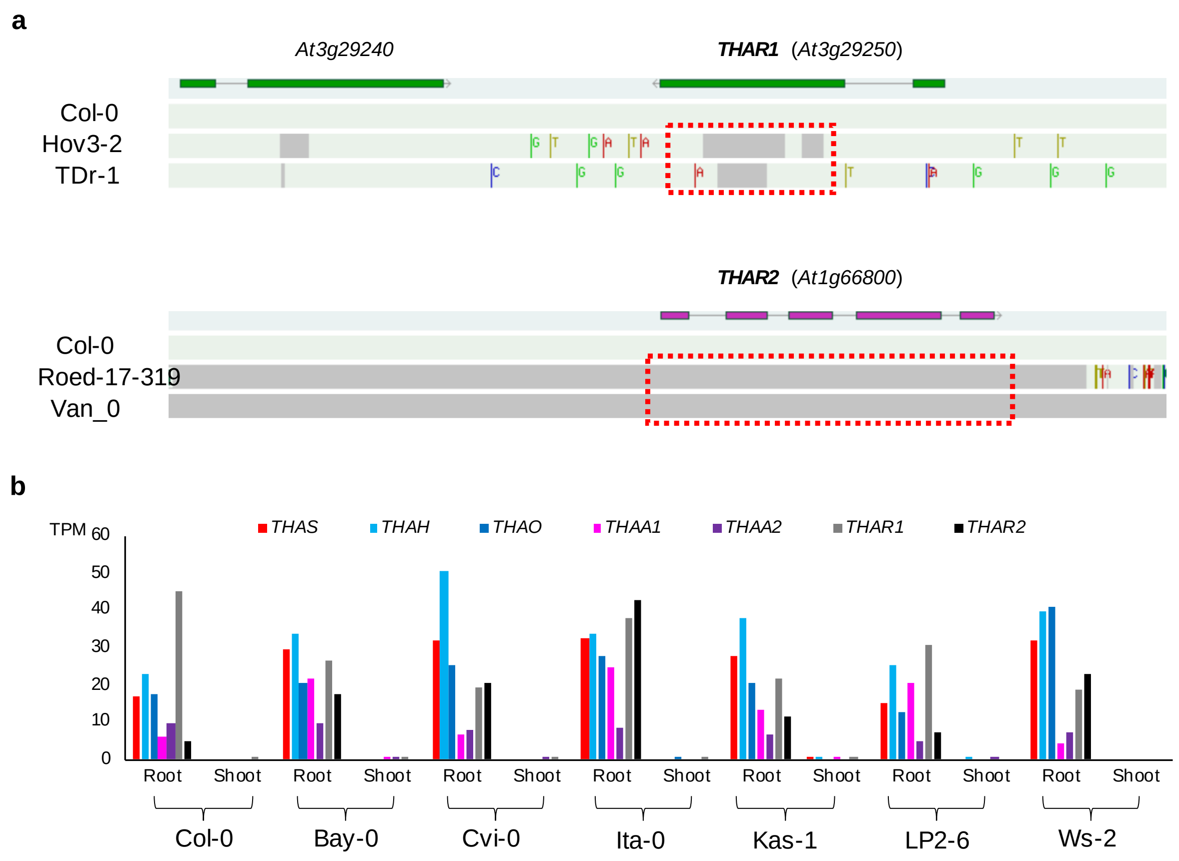

Supplementary Figure 3. Gene truncation or deletion of unlinked oxidoreductase genes in *A. thaliana* accessions and comparison of gene expression level for thalianin pathway genes in *A. thaliana* accessions. a, The two unlinked genes (*THAR1* and *THAR2*) are present in nearly all of the *A. thaliana* accessions. However, *THAR1* exhibits exon truncation in Hov3-2 and TDr-1, and *THAR2* is deleted in Röd-17-319 and Van_0. Gene truncations or deletions are indicated by the red dashed boxes. **b**, Gene expression levels are expressed as Transcripts Per Kilobase Million (TPM) values from transcriptome data (Materials and Methods). Source data underlying Supplementary Figure 3b are provided as a Source Data file.

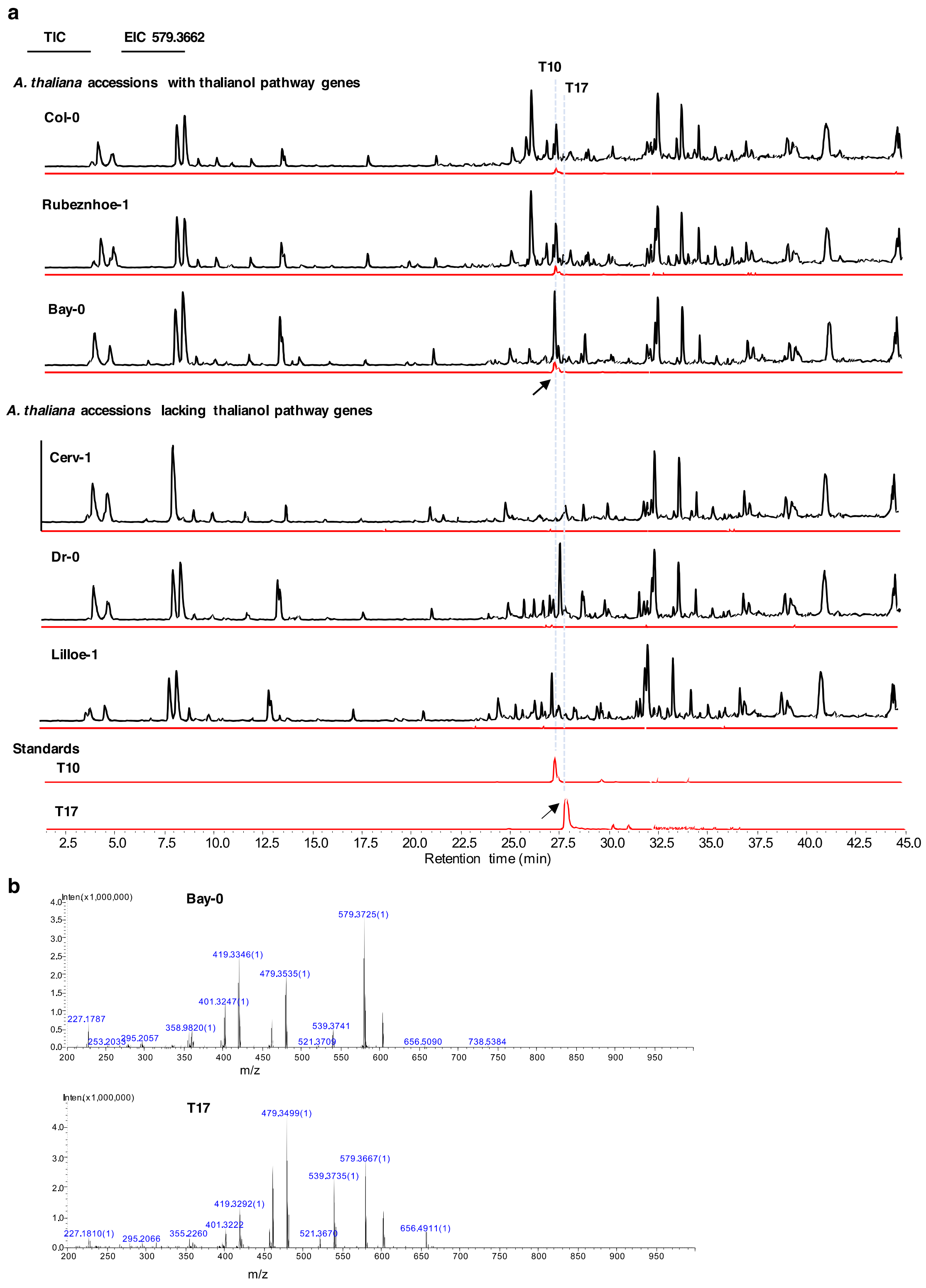

Supplementary figure 4. Metabolite analysis of root extracts from *A. thaliana* accessions with or without predicted thalianol biosynthetic genes. a, LC-MS-IT-TOF chromatograms. TIC: total ion chromatogram. EIC: extracted ion chromatogram. Mass fragment 579.36 was used for searching for the two epimers, **T10** and **T17**. **b**, Comparison of the MS spectra for the peak indicated by the arrow in Bay-0 (top) and the **T17** standard. Each experiments were independently repeated for three times.

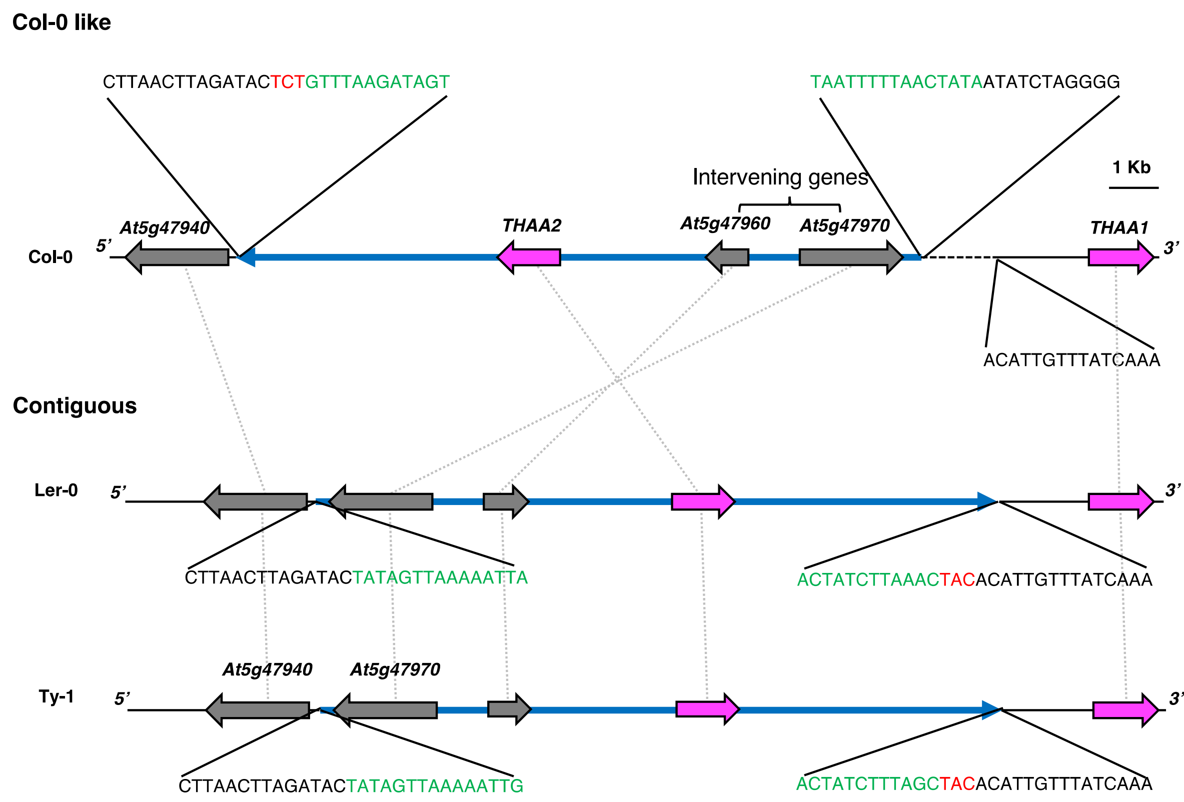

Supplementary Figure 5. Breakpoints for chromosomal inversions in representative re-sequenced *A. thaliana* accessions. Comparison of the thalianol cluster *THAA1* and *THAA2* region in Col-0 with the syntenic regions in other *A. thaliana* accessions (Ler-0 and Ty-1). The inverted region (*ca.* 15 Kb) is indicated by the blue arrows. Syntenic genes are connected by dashed gray lines. The sequences around the junctions are shown. Sequences unique to the 'Col-0-like' and 'contiguous' (Ler-0 and Ty-1) types are indicated in red.

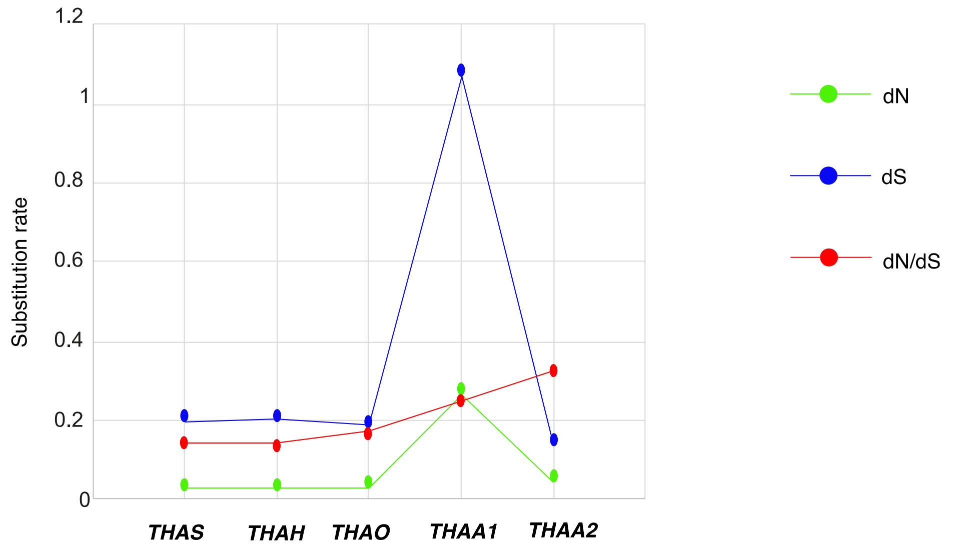

Supplementary Figure 6. The thalianol late pathway genes are under markedly reduced purifying selection relative to early pathway genes. Pairwise comparison of substitution rates among the protein coding sequences for the thalianol cluster genes from *A. lyrata* (VLH6) and *A. thaliana* (Col-0). *THAA1* showed five-fold elevated substitution rates (dN or dS) relative to other cluster genes, whereas *THAA2* showed the highest dN/dS ratio.

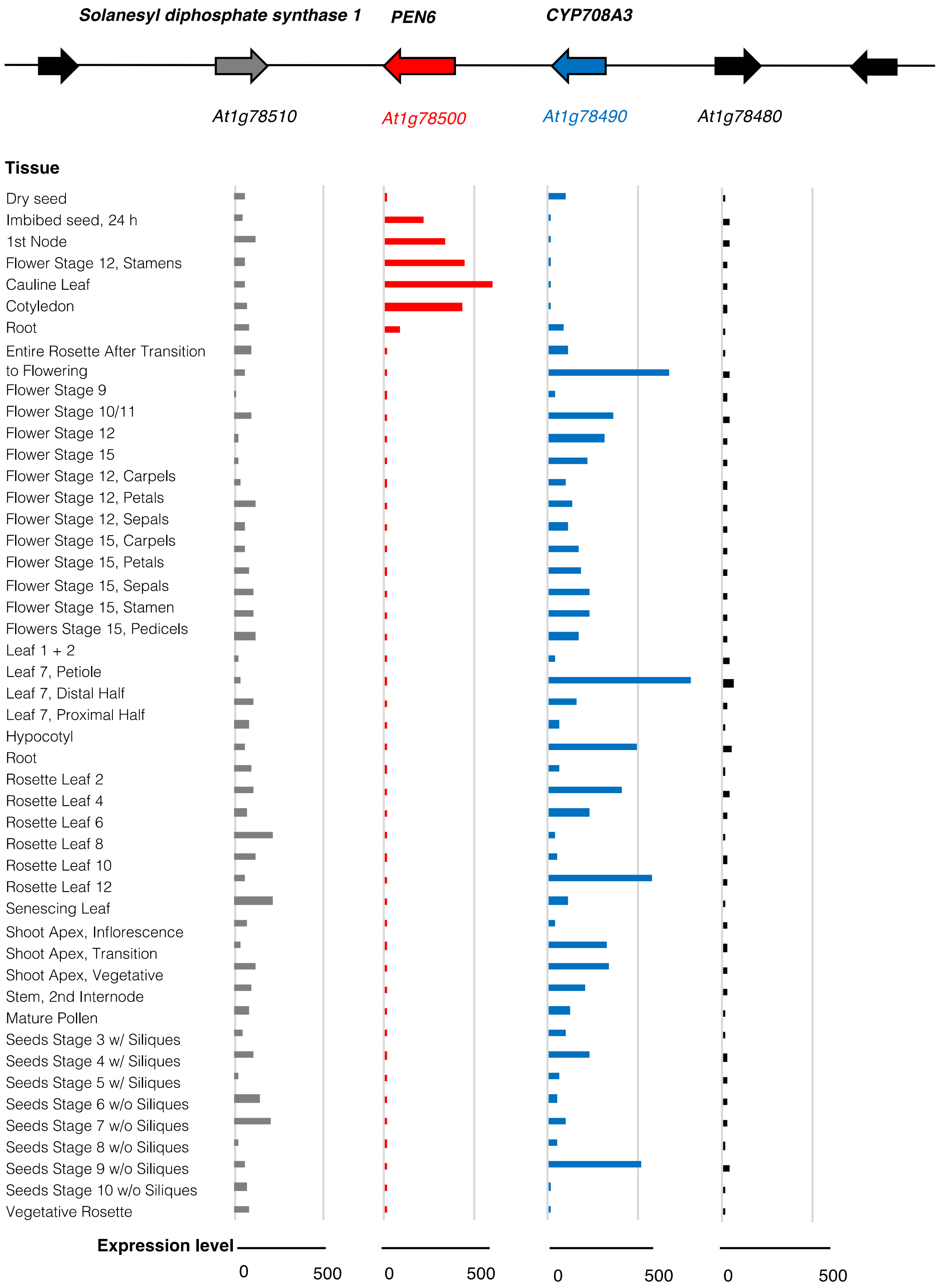

Supplementary Figure 7. Expression of *PEN6* and flanking genes in *A. thaliana*. Gene expression values were extracted from Arabidopsis eFP browser (https://bar.utoronto.ca/efp/cgi-bin/efpWeb.cgi). The expression levels were retrieved in the 'Absolute' mode.

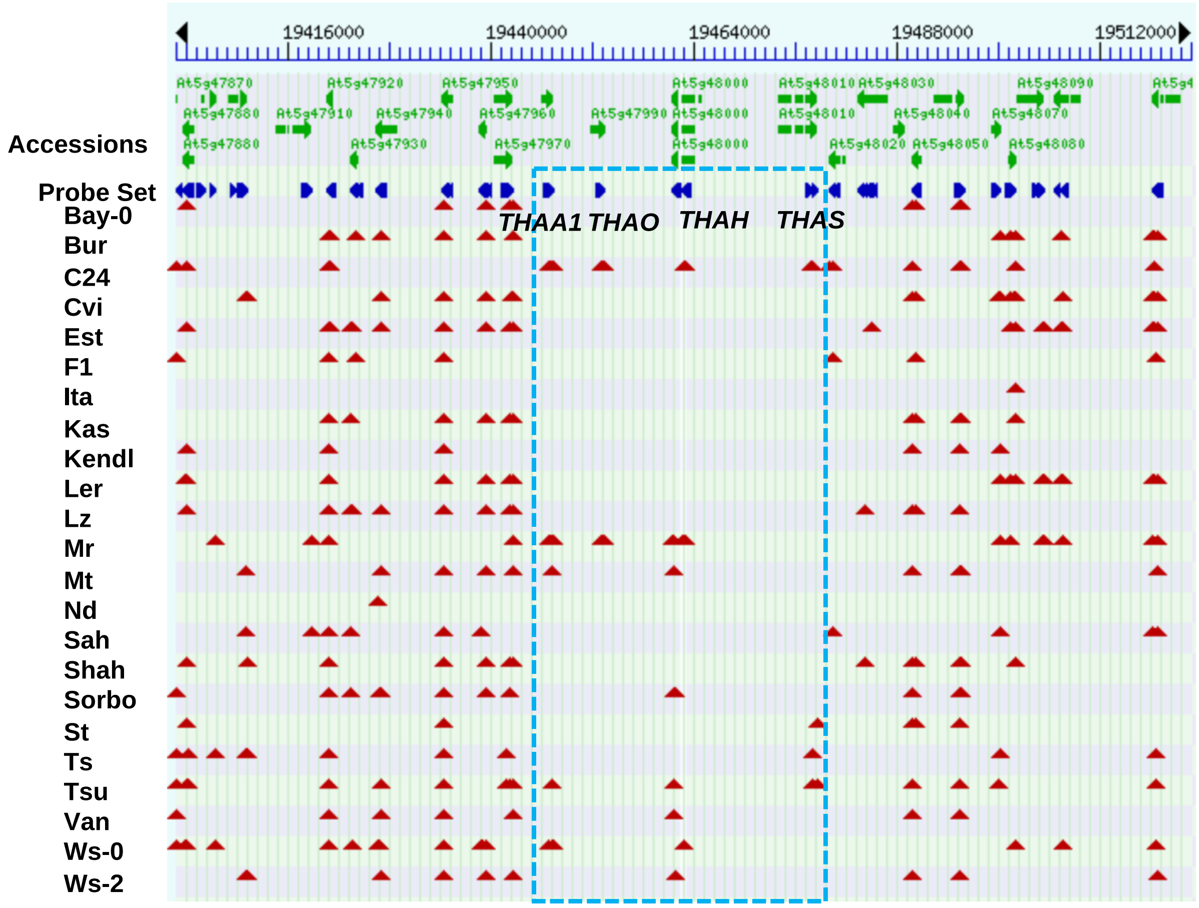

Supplementary Figure 8. Low levels of single feature polymorphisms in the thalianol cluster region. Single feature polymorphisms (SFPs) for the thalianol cluster region and surrounding genomic neighborhood were extracted from http://signal.salk.edu/cgi-bin/AtSFP. Those for the core thalianol cluster genes *THAS*, *THAH*, *THAO* and *THAA1* are framed by the blue dashed box. Of note, only a very limited number of accessions have been analysed by SFP read mapping.

Supplementary Table 1. Examination of polymorphisms in the thalianol cluster genes using POLYMORPH 1001.

			Impact				
			low	moderate	modifier	high	total
SNPs	*THAS*	*at5g48010*	90%	79%	90%	0.17% *	
	THAH	*at5g48000*	59%	52%	90%	4%	
	THAO	*at5g47990*	30%	40%	73%	0	
	THAA1	*at5g47980*	50%	80%	45%	3%	
	THAA2	*at5g47950*	85%	84%	72%	1%	
	Cluster level		96%	94%	94%	7%	**97%**
Small insertion	*THAS*	*at5g48010*	0	0	44%	0	
	THAH	*at5g48000*	0	0	16%	0	
	THAO	*at5g47990*	0	0	0	0	
	THAA1	*at5g47980*	0	1%	3%	6%	
	THAA2	*at5g47950*	0	0	0	0	
	Cluster level		0	1%	55%	6%	**55%**
Small deletion	*THAS*	*at5g48010*	0	0	0	0.08% *	
	THAH	*at5g48000*	1%	0	2%	2%	
	THAO	*at5g47990*	0	0	4%	0	
	THAA1	*at5g47980*	0	0	0	10%	
	THAA2	*at5g47950*	0	0	23%	2%	
	Cluster level		1%	0	28%	14%	**36%**

The POLYMORPH 1001 database possesses systematically examined polymorphisms for SNPs and Indels across the pangenomes [7]. Each polymorphism was assigned to different levels of impacts. 'High' impact is due to frame-shift variants. 'Modifier' impact is due to variants in the gene UTR region, which may affect gene expression. 'Moderate' impact is due to missense variants that cause non-synonymous substitution. 'Low' impact is due to synonymous substitution variants. Asterisks indicate 'High' impact mutations on the thalianol synthase (*THAS*) gene, which likely block the whole biosynthetic pathway. We examined all 1,135 accessions and checked how many accessions bearing the thalianol cluster genes had a specific variant, i.e. SNP. This value was used to divide the total number of examined accessions (1,135), giving rise to percent values for sequence variations.

Supplementary Table 2. BUSTED analysis.

Gene		ω			Statistics			
		ω_1	ω_2	ω_3	*log* L	#. params	AIC$_c$	P-value
THAS	Foreground	0.09 (77.79%)	0.65 (22.21%)	1.12 (0.00%)	-23839.8	65	47810	1
	Background	0.01 (71.38%)	0.67 (27.99%)	7.17 (0.63%)				
THAH	Foreground	0.16 (89.84%)	0.43 (10.16%)	1.00 (0.00%)	-23174.1	121	46592	1
	Background	0.06 (68.24%)	1.00 (31.42%)	746.40 (0.34%)				
THAO	Foreground	0.18 (70.95%)	0.48 (26.98%)	1.07 (2.08%)	-21372.6	79	42904	1
	Background	0.03 (62.59%)	0.95 (36.86%)	28.32 (0.55%)				
THAA1	Foreground	0.21 (69.97%)	0.22 (23.59%)	4.55 (6.44%)	-11604.5	59	23328	0.309
	Background	0.05 (67.41%)	0.45 (26.31%)	3.22 (6.28%)				
THAA2	Foreground	0.03 (73.87%)	0.44 (16.29%)	3.05 (9.85%)	-11607.1	59	23333	0.65
	Background	0.04 (63.54%)	0.44 (30.36%)	3.22 (6.10%)				

The output for gene-wide selection analysis using the Branch-Site Unrestricted Statistical Test for Episodic Diversification (BUSTED) method [8] are shown. This analysis fits a codon model with three constrained rate classes, $\omega_1 \leq \omega_2 \leq 1 \leq \omega_3$. The output for unconstrained model is shown. Statistical tests for positive selection on the foreground was compared to the constrained model where $\omega_3 \geq 1$ (i.e. disallowing positive selection). Foreground, branches tested for positive selection. Background, remaining branches. AIC$_c$ (small-sample Akaike Information criterion) was used to infer the optimal number of rate categories for each branch [9]. Bonferroni-Holm corrected $P < 0.05$ was considered as evidence for positive selection.

Supplementary Table 3. MEME analysis.

Gene	Codon	Partition	alpha	beta+	p+	LRT	p
THAS	34	1	0.445	10000.000	0.515	5.145	0.035
	324	1	0.000	9999.951	0.512	10.502	0.0023
	332	1	0.250	33.106	1.000	5.845	0.0244
	431	1	0.000	3.875	1.000	5.372	0.0311
THAH	157	1	0.000	183.399	0.513	14.261	0.0003
	249	1	1.710	1790.729	1.000	6.327	0.02
	291	1	0.000	3.107	1.000	4.765	0.0426
	361	1	0.174	12.436	1.000	4.870	0.0403
THAO	266	1	0.187	16.432	1.000	12.599	0.0008
	324	1	0.335	25.444	1.000	11.766	0.0012
	464	1	1.472	22.373	1.000	8.239	0.0072
THAA2	16	1	0.400	29.306	1.000	4.938	0.0389
	149	1	2.566	28.323	1.000	4.615	0.046
	232	1	1.119	20.884	1.000	4.744	0.043
	283	1	0.272	8.959	1.000	7.044	0.0132

The output for diversifying selection on individual sites using Mixed Effects Model of Evolution (MEME) method [10] are shown. Bonferroni-Holm corrected $P < 0.05$ was considered as evidence for positive selection. The sites with $P < 0.05$ for *THAS*, *THAH*, *THAO* and *THAAA2* are shown. The *THAA1* has been subjected to high mutation rate (Fig. S6), which caused long branch attractions between the two orthologs in *A. thaliana* and *A. lyrata* in the phylogenetic tree, so was not included in the analysis. The codon number corresponds to the codon site in the input alignment (Table S6) after trimming gaps and diversifying terminal sequences.

Supplementary Table 4. McDonald-Kreitman test.

	Gene	Fixed N	Fixed S	Polymorphic N	Polymorphic S	a	Fisher's exact (*P*)
**Standard MKT**	*THAS*	9	6	33	40	0.45	0.397
	THAH	9	9	25	20	-0.25	0.783
	THAO	9	11	22	21	-0.28	0.788
	THAA1	6	22	87	182	-0.753	0.288
	THAA2	9	29	13	23	-0.821	0.311
**FWW correction MKT**	*THAS*	9	6	33	40	0.529	0.337
	THAH	9	9	25	20	0.062	1
	THAO	9	11	22	21	-0.179	1
	THAA1	6	22	87	182	-0.147	1
	THAA2	9	29	13	23	-0.256	0.794
**Extended MKT**	*THAS*	9	6	33	40	0.542	0.247
	THAH	9	9	25	20	0.028	1
	THAO	9	11	22	21	-0.164	1
	THAA1	6	22	87	182	-0.275	0.813
	THAA2	9	29	13	23	-0.256	0.794

The output from different computations of McDonald and Kreitman analysis (MK test) are shown. The standard MKT compares the neutral (N) and selective (S) sites between fixed and polymorphic dataset. The Fay, Wycoff, and Wu (FWW) correction computed only those polymorphic sites with a frequency above the cutoff of 0.05. The extended MKT further considered the bias from relaxed purifying selection; thus, it could detect subtle signatures for positive selection. A positive alpha (α) with a $P < 0.05$ (two-sided Fisher's exact test) was used to detect the proportion of non-synonymous substitutions fixed by positive selection.

Supplementary Table 5. Assembly statistics for *A. arenosa*.

Number of reads	82.1 M
Number of contigs	10147
Number of scaffolds >= 10 Kb	959
Estimated genome size	214.09 Mb
Assembly size (only scaffolds >= 10 [illegible]	127.02 Mb
N50 contig size	89.23 Kb
N50 (only scaffolds >= 10 Kb)	2.19 Mb
N90 (only scaffolds >= 10 Kb)	43.3 Kb
Complete BUSCOs	97.50%
Complete and single-copy BUSCOs	92.80%
Complete and duplicated BUSCOs	4.70%
Fragmented BUSCOs	1.10%
Missing BUSCOs	1.40%

Supplementary Table 6. NCBI accession numbers for *de novo* assemblies of the thalianol cluster region.

A. thaliana **accessions**	**NCBI accession number**	**Sequence source**
Nyl-7	MT223943	1001 Genomes Plus Team
T690	MT223944	1001 Genomes Plus Team
Sq-1	MT223945	1001 Genomes Plus Team
Ws-2	MT223946	1001 Genomes Plus Team
Sf-2	MT223947	1001 Genomes Plus Team
IP-Cum-1	MT223948	1001 Genomes Plus Team
Stiav-1	MT223949	1001 Genomes Plus Team
Erg2-6	MT223950	1001 Genomes Plus Team
IP-Pva-1	MT223951	1001 Genomes Plus Team
IP-Ven-0	MT223952	1001 Genomes Plus Team
RAD-21	MT223953	1001 Genomes Plus Team
An-1	MT223954	Jiao and Schneeberger[11]
Col-0	MT223955	Tair 10
Cvi-0	MT223956	Jiao and Schneeberger[11]
Eri	MT223957	Jiao and Schneeberger[11]
KBS-Mac-74	MT223958	Michael *et al.*[12]
Kn-0	MT223959	1001 Genomes Plus Team
Kyo	MT223960	Jiao and Schneeberger[11]
Ler-0	MT223961	Zapata *et al.* [13]
Sha	MT223962	Jiao and Schneeberger, 2020[11]
Ty-1	MT223963	1001 Genomes Plus Team
Lillö-1	MT223964	1001 Genomes Plus Team
Nd-1	MW035341	Pucker *et al.* [14]

Supplementary References

1. Field, B. & Osbourn, A. E. Metabolic diversification-independent assembly of operon-like gene clusters in different plants. *Science* **320**, 543–547 (2008).
2. Liu, Z. *et al.* Drivers of metabolic diversification: how dynamic genomic neighbourhoods generate new biosynthetic pathways in the Brassicaceae. *New Phytol.* https://doi.org/10.1111/nph.16338 (2019).
3. Kautsar, S. A., Suarez Duran, H. G., Blin, K., Osbourn, A. & Medema, M. H. PlantiSMASH: automated identification, annotation and expression analysis of plant biosynthetic gene clusters. *Nucleic Acids Res.* **45**, W55–W63 (2017).
4. Thimmappa, R., Geisler, K., Louveau, T., O'Maille, P. & Osbourn, A. Triterpene biosynthesis in plants. *Annu. Rev. Plant Biol.* **65**, 225–257 (2014).
5. Huang, A. C. *et al.* A specialized metabolic network selectively modulates Arabidopsis root microbiota. *Science* **364**, eaau6389 (2019).
6. Sohrabi, R. *et al. In planta* variation of volatile biosynthesis: an alternative biosynthetic route to the formation of the pathogen-induced volatile homoterpene DMNT via triterpene degradation in Arabidopsis roots. *Plant Cell* **27**, 874–890 (2015).
7. 1001 Genomes Consortium. 1,135 genomes reveal the global pattern of polymorphism in *Arabidopsis thaliana*. *Cell* **166**, 481–491 (2016).
8. Murrell, B. *et al.* Gene-wide identification of episodic selection. *Mol. Biol. Evol.* **32**, 1365–1371 (2015).
9. Sugiura, N. Further analysts of the data by akaike' s information criterion and the finite corrections. *Commun. Stat. - Theory Methods* **7**, 13–26 (1978).
10. Murrell, B. *et al.* Detecting individual sites subject to episodic diversifying selection. *PLoS Genet.* **8**, e1002764 (2012).
11. Jiao, W.-B. & Schneeberger, K. Chromosome-level assemblies of multiple Arabidopsis genomes reveal hotspots of rearrangements with altered evolutionary dynamics. *Nat. Commun.* **11**, 989 (2020).
12. Michael, T. P. *et al.* High contiguity *Arabidopsis thaliana* genome assembly with a single nanopore flow cell. *Nat. Commun.* **9**, 541 (2018).
13. Zapata, L. *et al.* Chromosome-level assembly of *Arabidopsis thaliana* Ler reveals the extent of translocation and inversion polymorphisms. *Proc. Natl. Acad. Sci. USA* **113**, E4052 LP-E4060 (2016).
14. Pucker, B. *et al.* A chromosome-level sequence assembly reveals the structure of the *Arabidopsis thaliana* Nd-1 genome and its gene set. *PLoS ONE* **14**, e0216233 (2019).
