# Supplementary material for: Formation and diversification of a paradigm biosynthetic gene cluster in plants
Source: Nat Commun. 2020 Oct 23;11:5354. doi: 10.1038/s41467-020-19153-6 (PMC7584637; doi:10.1038/s41467-020-19153-6)
Supplement: Supplementary file 3 — Description of Additional Supplementary Files [file 41467_2020_19153_MOESM3_ESM.pdf]

Description of Additional Supplementary Files

File name: Supplementary Data 1

Description: Input files for Hyphy analysis
