# Supplementary material for: Formation and diversification of a paradigm biosynthetic gene cluster in plants
Source: Nat Commun. 2020 Oct 23;11:5354. doi: 10.1038/s41467-020-19153-6 (PMC7584637; doi:10.1038/s41467-020-19153-6)
Supplement: Supplementary file 4 — Supplementary Data 1 [file 41467_2020_19153_MOESM4_ESM.docx]

**Supplementary data 1**

**Input files for Hyphy analysis**

**A) OSC codon-based alignment and input for Newick tree**

>AT2G07050

ATGTGGAAACTGAAGATCGCGGAAGGACCATGGCTTAGAACCACCAATAATCACGTCGGAAGACAGTTTTGGGAGTTCGATCCGAATCTCGGTACTCCTGAGGATCTCGCCGCCGTCGAAGAAGCTAGGAAGTCTTTTTCGGATAATCGATTCGTGCAGAAAAGCGCCGATCTGCTTATGCGCCTTCAGTTTTCAAGAGAAAATTTGATTAGCCCAGTTTTACCTGTCAAAATCGAAGACACAGAGGAGATGGTGGAAACCACGTTAAAGAGGGGTCTAGATTTCTATTCAACTATACAGGCACACGACGGGCACTGGCCAGGTGATTATGGTGGTCCTATGTTTCTTCTCCCAGGACTGATAATTACACTCTCCATAACTGGAGCACTGAATACAGTATTGTCGGAACAACATAAACAAGAAATGCGCCGTTATCTCTATAATAATGAGGACGGAGGTTGGGGTTTACATATTGAGGGCCCTAGCACCATGTTTGGGTCTGTGTTGAACTATGTTACTCTAAGGTTGCTTGGAGAAGGACCTGATATGGAGAAAGGACGAGACTGGATACTAAATCATGGTGGTGCTACCAATATTACATCTTGGGGGAAAATGTGGCTATCGGTACTTGGAGCTTTTGAATGGTCCGGAAATAACCCACTGCCACCTGAGATATGGCTTCTCCCATATTTCCTGCCAATACATTGCCATTGTCGAATGGTGTACTTGCCGATGTCGTATTTGTATGGAAAAAGGTTTGTGGGTCCCATAACGTCCACTGTTTTATCACTGAGAAAGGAGCTTTTCACAGTACCATATCATGAAGTCAACTGGAATGAAGCACGCAACCTTTGCGCAAAGGAGGATTTATACTACCCACATCCACTTGTGCAAGATATTCTTTGGGCATCACTTCATAAGATTGTTGAGCCTGTTCTGATGCGATGGCCTGGTGCAAATTTG---AGAGAAAAGGCTATAAGAACCGCAATAGAACATATTCATTATGAAGATGAGAATACTAGGTACATCTGCATAGGTCCCGTGAACAAGGTATTAAATATGCTTTGCTGTTGGGTAGAAGACCCAAACTCAGAGGCTTTCAAGTTGCACCTACCAAGAATCCATGACTTTCTCTGGTTAGCTGAAGATGGAATGAAGATGCAGGGTTATGGAAGCCAGCTATGGGATACAGGTTTTGCTATTCAAGCGATTTTGGCAGTTTTGGAAAAAGCACATTCATTTGTCAAGAATTCCCAGGTGTTAGAAGACTGCCCTGGAGATCTGAATTACTGGTATCGCCACATTTCTAAAGGGGCTTGGCCTTTCTCAACTGCAGATCACGGTTGGCCCATCTCTGACTGCACCGCAGAAGGACTGAAAGCTGCTCTTTTGCTATCCAAAGTTCCCAAGGCGATTGTTGGTGAACCAATAGATGCAAAACGGTTATATGAAGCTGTTAATGTTATCATTTCTTTACAGAATGCAGATGGAGGCCTCGCAACATATGAGCTCACCAGGTCATACCCTTGGTTAGAGCTAATCAACCCAGCAGAAACCTTTGGCGATATTGTTATTGATTATCCTTACGTGGAATGTACATCAGCTGCTATCCAAGCTTTGATATCATTTCGAAAGCTGTATCCTGGTCATCGAAAGAAGGAAGTAGATGAGTGCATTGAGAAGGCGGTTAAGTTCATTGAATCCATTCAAGCAGCAGATGGCTCATGGTATGGATCATGGGCTGTTTGCTTCACGTATGGTACGTGGTTTGGAGTGAAAGGGCTGGTAGCTGTTGGAAAAACATTGAAAAACTCTCCACATGTTGCTAAAGCTTGTGAATTTCTATTGTCGAAACAACAACCTTCGGGCGGCTGGGGAGAAAGCTATCTTTCATGTCAAGACAAGGTCTATTCAAACCTTGATGGCAACAGATCTCACGTCGTGAATACAGCATGGGCTATGCTCGCACTCATTGGTGCTGGGCAAGCTGAGGTAGACCGGAAACCACTACACCGGGCTGCAAGATACTTGATTAATGCTCAAATGGAGAATGGTGATTTTCCACAACAGGAAATAATGGGAGTCTTCAATAGGAACTGCATGATAACATATGCCGCGTATCGAAACATTTTTCCGATATGGGCTTTGGGGGAGTACCGT

>AL3G44070

ATGTGGAAACTGAAGATCGCGGAAGGACCATGGCTTAGAACCACCAATAATCACGTCGGAAGACAGTTTTGGGAGTTCGATCCGAATCTCGGTACTCCTGAGGATCTCGCCGCCGTCGAGGAAGCTAGGAAGTCTTTTTCAGATAACCGATTCTTGCAGAAAAGCTCCGATCTGCTTATGCGCCTTCAGTTTTCAAGAGAAAATTTGATTAGCCCAGTTTTACCTGTCAAAATCGAAGATACAGAGGAGATGGTGGAAAACACGTTAAAGAGGGGTGTAGATTTCTATTCAACAATACAGGCGCACGACGGGCACTGGCCAGGTGATTATGGTGGTCCTATGTTTCTTCTCCCAGGACTGATAATTACACTCTCCATAACTGGAGCACTGAATACAGTATTGTCGGAACAACATAAACAAGAAATGCGCCGTTATCTCTTTAATAATGAGGACGGAGGTTGGGGTTTACATATTGAGGGCCCTAGCACCATGTTTGGGTCTGTGTTGAACTATGTTACTCTAAGGTTGCTTGGAGAAGGACCTGCTATGGAGAAAGGGCGAGACTGGATACTAAATCATGGTGGTGCTACCAATATCACATCTTGGGGGAAAATGTGGCTATCGGTACTTGGAGCTTTTGAATGGTCTGGAAATAACCCACTGCCACCTGAGATATGGCTTCTTCCATATTTCCTGCCAATTCATTGCCATTGTCGGATGGTGTACTTGCCGATGTCGTATTTGTATGGAAAAAGGTTTGTGGGTCCCATAACGTCCACTGTTTTATCACTGAGAAAGGAGCTTTTCACTGTACCATATCATGAAGTCAACTGGAATGAAGCACGCAACCTTTGCGCAAAGGAGGATTTATACTACCCACACCCACTTGTGCAAGATATTCTTTGGGCATCACTTCATAAGATTGTTGAGCCTGTTCTGATGCGATGGCCTGGTGCAAATTTG---AGAGAAAAGGCTATAAGAACCGCAATAGAACATATTCATTATGAAGATGAGAATACTAGGTACATCTGCATAGGTCCCGTGAACAAGGTATTAAATATGCTTTGCTGTTGGGTAGAGGACCCAAACTCAGAGGCTTTCAAGTTGCACCTACCAAGAATCCATGACTTTCTCTGGCTAGCTGAAGATGGAATGAAGATGCAGGGTTATGGAAGTCAGCTATGGGATACAGGTTTTGCTATTCAAGCGATTTTGGCAGTTTTGAAAAAAGCACATTCATTTGTCAAGAATTCTCAGGTGTTAGAAGACTGTCCTGGAGATCTGAATTACTGGTACCGCCACATTTCTAAAGGGGCTTGGCCTTTCTCAACTGCAGATCACGGTTGGCCCATCTCCGACTGCACCGCAGAAGGACTGAAAGCTGCTCTTTTGTTATCCAAAGTTCCCAAAGAGATTGTTGGTGAACCAATAGATGCAAAACGGTTATATGATGCTGTTAATGTTATCATTTCATTACAGAATGCAGATGGAGGCCTCGCAACATATGAGCTCACCAGGTCATACCCTTGGTTGGAGCTAATCAACCCAGCAGAAACCTTTGGCGATATTGTTATAGATTATCCTTACGTAGAATGTACATCAGCTGCTATCCAAGCTTTGATATCATTCCGAAAGCTGTATCCTGGTCATCGGAAGAAGGAAGTAGATGAGTGCATTGAGAAGGCGGTTAAGTTCATTGAATCTATTCAAGCAGCAGATGGCTCATGGTATGGATCATGGGCTGTTTGCTTCACATATGGTACGTGGTTTGGAGTGAAAGGGCTGGTAGCTGTTGGAAAGACATTGAAAAACTCTTCACATGTTGCCAAAGCTTGCGAATTTCTATTGTCTAAACAACAACCTTCGGGCGGATGGGGAGAAAGCTATCTTTCATGTCAAGACAAGGTATATTCAAACCTTGAAGGCAACAGGTCTCATGTCGTGAATACAGCATGGGCTATGCTCGCACTCATTGGTGCTGGGCAAGCTGAGGTAGATCAAAAACCACTAGACCGGGCTGCAAGATACTTGATTAATGCTCAGATGGAGAATGGTGATTTTCCACAACAGGAAATAATGGGAGTCTTCAATAGGAACTGCATGATAACATATGCTGCGTATCGAAACATTTTTCCGATATGGGCATTGGGGGAGTACCGT

>AT3G45130

ATGTGGAGGTTAAAGTTATCGGAAGGA---------GAGAGCGTGAATCAACATGTTGGAAGACAGTTTTGGGAATATGATAACCAATTTGGAACCTCTGAAGAGAGACATCACATTAACCATCTTCGTAGCAACTTTACTCTCAATCGGTTTTCTTCTAAGAGTTCTGATCTTCTCTACCGTTTTCAGTGTTGGAAAGAGAAAAAAGGAATGGAGAGACTTCCAGTGAAAGTAAAAGAGAATGAAGAAGTGGTGAATGTTACATTAAGAAGAAGTTTGAGATTCTACTCAATACTTCAATCACAAGATGGTTTTTGGCCTGGTGATTATGGTGGCCCTTTGTTTCTCTTGCCTGCTCTGGTGATCGGCTTATATGTGACGGAAGTTTTGGATGGAACTTTAACTGCGCAACATCAAATCGAGATTCGTCGTTATCTCTATAACAACAAGGATGGAGGATGGGGACTACACGTAGAAGGGAATAGCACCATGTTTTGTACAGTGCTCTCATACGTAGCACTGAGACTAATGGGGGAAGAATTAGCCATGGAATCAGCTAGAAGTTGGATTCACCACCACGGTGGTGCCACTTTTATTCCCTCCTGGGGCAAGTTCTGGCTCTCCGTTCTTGGAGCTTATGAGTGGAGTGGGAACAATCCTTTACCTCCAGAGCTATGGCTCCTTCCATATAGTCTTCCTTTTCATTGCCATTGTAGGATGGTTTATCTTCCAATGTCATATCTATATGGAAGAAGATTTGTTTGTCGTACTAATGGAACTATTTTATCGCTTCGACGAGAGCTCTACACTATTCCTTATCACCATATCGATTGGGACACCGCCCGTAATCAATGTGCCAAGGAGGACTTGTACTATCCACATCCAAAGATTCAAGACGTCCTTTGGAGTTGTCTGAATAAATTTGGAGAGCCTCTTCTTGAAAGATGGCCATTGAATAACCTC---AGAAACCATGCTCTTCAGACAGTAATGCAACACATTCACTATGAAGACCAAAACAGCCACTATATTTGTATCGGTCCTGTCAACAAAGTCTTGAATATGCTTTGTTGTTGGGTCGAGTCCTCGAATTCCGAGGCATTTAAATCTCACCTCTCGCGGATTAAAGACTATTTGTGGGTGGCTGAGGATGGAATGAAAATGCAGGGATACGGATCTCAGCTGTGGGACGTGACTTTAGCGGTCCAAGCAATCTTGGCGATGCTTAAGAAAGCCCATAACTACATCAAGAACACTCAAATAAGGAAAGACACAAGTGGAGATCCGGGGTTGTGGTACCGACACCCGTGCAAGGGAGGATGGGGTTTCTCCACTGGAGACAATCCATGGCCTGTCTCTGATTGTACTGCTGAAGCCTTGAAGGCGGCGTTGCTATTGTCACAAATGCCGGTTAATTTAGTTGGAGAACCCATGCCTGAAGAGCATTTAGTTGATGCTGTAAACTTTATCTTATCATTACAGAACAAGAATGGGGGATTTGCGTCATATGAGCTAACTAGATCATATCCCGAGCTAGAGGTTATCAACCCATCAGAGACTTTTGGGGATATCATCATAGATTATCAATACGTAGAATGCACGTCAGCTGCCATCCAAGGTCTCGTGTTATTCACAACGTTAAATTCGAGCTACAAGAGGAAGGAGATAGTAGGAAGCATCAACAAAGCAGTTGAGTTTATTGAAAAAACACAACTTCCTGATGGTTCATGGTATGGCTCGTGGGGAGTGTGTTTCACCTATGCAACATGGTTTGGTATTAAAGGCATGTTGGCTTCAGGCAAAACATATGAGAGCAGTCTTTGTATTAGAAAAGCTTGTGGTTTCTTGCTCTCCAAACAACTTTGTTGTGGTGGATGGGGAGAGAGCTACCTTTCTTGCCAAAACAAAGTATACACCAATCTTCCTGGGAATAAATCGCATATTGTGAACACATCATGGGCACTCTTGGCTCTCATTGAAGCGGGACAAGCTAGTAGAGATCCGATGCCATTGCATCGCGGGGCAAAATCGCTGATCAACTCGCAGATGGAAGATGGAGATTACCCACAACAAGAGATACTAGGAGTCTTTAATCGGAATTGTATGATCAGTTACTCAGCTTATAGAAACATATTCCCCATTTGGGCTCTTGGAGAATACCGC

>AL5G23630

ATGTGGAGGTTAAAGTTATCGGAAGGA---------GAGAGTGTGAATCAACATGTTGGAAGACAGTTTTGGGAGTATGATAACCAATTTGGAACCTCTGAAGAGAGACATCACATCGCCGATCTTCGGGGCAACTTTACTCTCAATCGGTTTTCTTCTAAGAGTTCTGATCTTCTCTACCGTTTTCAGTGTTGGAAAGAGGAAAAAGGAAAGGAGAGGCTTCCAGTGAAAGTAAAAGAGAATGAAGAAGTGGTGAATGTAACATTAAGAAGAAGTTTGAGATTCTACTCAACACTTCAATCACAAGATGGGTTTTGGCCTGGTGATTATGGTGGCCCTTTGTTTCTCTCGCCTGCTCTGGTGATCAGCTTATATGTGACAGAAGTTTTAGACGCAACTTTAACTGCTCAACATCAAATGGAGATTCGTCGTTATCTCTATAACAACAAGGATGGAGGATGGGGACTACACATAGAAGGGAGTAGCACCATGTTCTGTACGGCTCTCTCATACGTAGCACTAAGGCTCATGGGGGAAGAAATGGCCATGGAATCAGCTAGACTTTGGATTCACCACCGTGGTGGTGCCACCTTTGTTCCCTCTTGGGGCAAGTTCTGGCTCTCCGTTCTTGGAGCTTATGAATGGAGTGGCAACAATCCTTTACCTCCAGAGCTATGGCTCCTTCCTTATAGTCTTCCATTTCATTGCCATTGTAGGATGGTTTATCTTCCAATGTCATATCTATACGGAAGAAGATTTGTTTGTCGCACTAATGGAACTATTTTGTCCCTTCGACGAGAGCTTTACACTGTTCCTTATCATCATATCGATTGGGACACGGCTCGTAATCAATGTGCCAAGGAGGACTTGTACTATCCACATCCAAAGATTCAAGACGTTCTTTGGAGTTGTCTGAATAAATTTGGAGAGCCTCTTCTTGAAAGATGGCCATTGAATAAGCTA---AGAAGCCGGGCTCTTCAGACAGTGATGCAACATATTCAATATGAAGACCAAAACAGCCACTATATTTGTATCGGTCCTGTCAATAAAGTCTTGAATCTTCTTTGTTGTTGGGTTGATTCCTCGAATTCCGAGGCATTTAAATCTCACCTCTCGCGTATTAAAGACTATTTATGGGTGGCTGAGGATGGAATGAAAATGCAGGGATACGGATCTCAGCTGTGGGACGTGACTTTAGCGGTCCAAGCAATCTTGGCTATGCTTAAGAGAGCGCATAACTACATCAAGAACACTCAAATAAGGAAAGACACTTGTGGAGATCCGGGGTTGTGGTACCGACACCCGTGCAAGGGAGGATGGGGTTTCTCCACCGCAGACAATCCATGGCCTGTTTCTGACTGTACTGCTGAAGCCTTGAAGGCGTCGTTGTTATTGTCACAGATACCAGTTGATTTGGTTGGAGAAGCCATGCCTGAAGAGCATTTATTTGATGCTGTTGACTTTATCTTATCATTACAGAACAACAATGGGGGATTTGCGTCATACGAGCTAACTAGATCATATCCCGCGCTAGAGGTGATCAATCCATCAGAGACTTTTGGGGATATCATCATAGATTATCAATACGTAGAATGTACGTCAGCTGCAATCCAAGGTCTTGCGTTATTCACAACGATCAATCCAACATACAAGAGGAAAGAGATACTAATATGCATTAACAAAGCAGTTGAGTTTATTGAAAAAACACAACTTCCGGATGGTTCATGGTACGGCTCGTGGGGAGTGTGTTTCACCTATGCGACATGGTTTGGTATTAAAGGGATGTTAGCTGCTGGCAAAACATATGAGACCAGTCTTTGTATTAGAAAAGCTTGTGGTTTCTTGCTCTCCAAACAACTTTGTTGTGGTGGGTGGGGAGAGAGCTACCTTTCTTGCCAAAACAAAGTATACACCAATCTTCCAGGGAACAAATCGCATATCGTGAACACATCATGGGCAGTTTTGGCTCTCATTGAAGCAGGACAAGCTAATAGAGACCTGATGCCATTGCATCGCGGGGCAAAATCGCTGATCAACTCGCAGATGGAAGACGGAGATTACCCTCAACAGGAGATATTGGGAGTCTTTAATCGGAATTGTATGATCAGCTACTCAGCTTATAGAAACATATTCCCTATTTGGGCTCTAGGAGAGTACCAC

>AT5G42600

ATGTGGAGACTGCGAATTGGAGCTGAGCCTCACTTGTTCACCACGAACAACTTTGCCGGAAGACAGATCTGGGAGTTTGATGCCAACGGGGGCTCTCCTGAGGAACTCGCCGAGGTCGAGGAGGCTCGCCTCAATTTCGCAAACAACAAGTCACGTTTCAAGAGTCCCGATCTCTTCTGGCGTAGGCAGTTTCTGAGGGAGAAAAAGTTCGAGCAGAAGATCCCGGTGAGAATAGAGGATACATATGAAGACGCAAAGACGGCACTAAGAAGAGGAGTACTCTATTACGCGGCATGTCAGGCTAACGATGGCCATTGGCCTTCTGAAGTCTCTGGTTCCATGTTCTTGGACGCTCCCTTTGTGATATGCTTGTACATTACCGGACACCTGGAGAAAATCTTTACACTAGAGCATGTCAAAGAGTTACTCCGTTACATGTATAACAATGAAGATGGTGGGTGGGGGTTAGATGTGGAAAGCCACAGTGTCATGTTTTGCACGGTCCTCAACTACATCTGCTTACGTATTTTGGGAGTAGAACCAGCATGCGCAAGGGCTCGTAAATGGATCCTAGACCATGGGGGTGCCACGTATGCGCCTATGGTGGCTAAAGCTTGGCTTTCGGTTCTAGGAGTGTATGACTGGTCTGGTTGCAAACCGCTACCACCCGAGATTTGGATGCTCCCTTCTTTTTCTCCCATTAATATATATATCCGGGATCTTCTCATGGGCATGTCATATTTGTATGGTAAAAAATTTGTAGCTACACCAACCGCTCTCATTCTACAGCTCCGAGAAGAACTTTATCCCCAGCCTTACAGCAAAATCATTTGGAGCAAAGCCCGTAATCGATGCGCAAAGGAAGATCTATTATATCCAAAGTCATTTGGACAAGATTTGTTTTGGGAAGGTGTTCATATGCTTTCGGAGAATATCATAAACCGCTGGCCTCTCAACAAGTTTGTTAGACAAAGAGCACTACGAACCACAATGGAACTTGTTCACTACCATGATGAAACGACCCACTATATCACAGGTGCATGTGTTGCGAAGCCGTTTCATATGCTTGCTTGTTGGGTAGAAGATCCTGATGGTGATTATTTCAAGAAACATCTTGCTCGAGTCCCTGATTTCATATGGATTGCTGAAGATGGCCTTAAATTTCAGTTAATGGGGATGCAGTCGTGGAATGCAGCCCTCTCCCTCCAAGTTATGTTAGCCACCCTGATAAAAGGATACGACTTCTTGAAGCAATCTCAGATTAGCGAGAACCCTCAAGGTGACCATCTGAAAATGTTTAGAGACATTACAAAAGGGGGATGGACTTTTCAAGATCGAGAACAAGGGTTGCCGATTTCGGATGGTACAGCAGAAAGTATAGAGTGCTGCATACACTTCCACCGCATGCCCTCCGAGTTCATCGGCGAAAAAATGGATGTAGAGAAGCTCTATGATGCCGTCAATTTTCTAATCTATTTGCAGAGTGATAATGGAGGTATGCCAGTTTGGGAGCCAGCTCCTGGAAAAAAATGGCTAGAGTGGCTTAGTCCGGTGGAGCATGTGGAGAACACAGTTGTGGAGCAAGAGTACCTTGAGTGTACGGGCTCGGTGATTGCGGGATTGGTTTGCTTCAAGAAAGAGTTTCCCGATCACAGACCGAAAGAGATCGAAAAGTTAATAAAAAAAGGCCTAAAATACATAGAGGACTTGCAAATGCCGGACGGTTCATGGTACGGAAACTGGGGAGTTTGTTTCACGTATGGTACTCTCTTTGCAGTAAGAGGTCTAGCGGCTGCAGGGAAGACTTTTGGCAACTCTGAAGCTATACGTAGAGCTGTTCAGTTTATTCTCAACACGCAAAACGCCGAAGGCGGTTGGGGAGAAAGTGCTCTCTCTTGCCCTAACAAGAAATATATTCCTTCTAAAGGAAACGTGACGAATGTGGTTAATACCGGACAAGCTATGATGGTTTTACTTATTGGTGGTCAGATGGAGAGGGACCCTTCTCCTGTTCATCGCGCCGCCAAAGTGTTGATTAACTCACAGTTGGATATTGGCGATTTCCCACAACAGGAAAGAAGGGGGATCTACATG---AATATGCTGCTGCATTATCCAACCTATAGAAACATGTTCTCTCTTTGGGCTCTCGCATTGTACACA

>AL8G20190

ATGTGGAGGCTGAGACTTGGACCGAAGACTCACTTGTTCACCACCAACAACTATGCCGGGAGGCAGATTTGGGAGTTCGATGCCAACGCAGGCTCTCCAGAGGAACTTGCCGAGGTAGAGGATGCTCGTCAGAATTTTTCAAACAACCGGTCACGTTTCAAGAGTGCAGATCTCCTATGGCGGATGCAGTTTCTCAGGGAGAAGAAATTCGAGCAAAAGATTCCAGTGATAGTAGAGGATAAGTACGAAGATGCAAAGACCGCATTGAGAAGAGGGTTACTCTATTTCACAGCCTTGCAGGCTGATGATGGACACTGGCCAGCTGAAAACTCTGGTCCAAATTTCTACGCCCCACCTTTTTTGATATGCCTGTACATCACTGGAAATCTGGAGAAAATCTTCACTCCCGAGCATGTTAAAGAGTTACTACGTCACATCTACAACAACGAAGATGGTGGGTGGGGATTACACGTAGAAAGCCACAGTGTTATGTTCTGTACAGTCATCAACTACATTTGTTTAAGAATTGTGGGCGAAGAAGCCGGTTGTGCAAAGGCTCATAAGTGGATCATGGACCATGGTGGTGCTACCTACACGCCCTTGATAGGAAAAGCGTTACTATCGGTTCTTGGAGTGTATGATTGGTCTGGATGCAATCCTATACCTCCAGAGTTCTGGTTGCTTCCGTCTTCTTTTCCTGTTAATATTTATTTACGGGATACTTTTATGGGGTTGTCATACTTGTATGGTAAAAAATTTGTGGCTGCCCCAACACCTCTCATTCTCAAGCTCCGAGAAGAGCTTTATCCGGAGCCTTATGCAAAAATCAATTGGACGCAAACACGAAACCGATGTGCAAAGGAAGATCTCTACTATCCACGCTCATTTTTACAAGATTTGTTTTGGAAAAGTGTTCACATGTTCTCGGAGAGTATCCTAGATCGATGGCCTTTAAACAAACTAATAAGAGAGAGAGCTCTTCGATCCACTATGTCACTCATTCACTATCATGATGAATCCACCAGATATATTACAGGCGGATGCCTGCCAAAGGCCTTTCATATGCTTGCATGTTGGATAGAAGACCCTAAGAGTGATTATTTTAAAAAACATCTTGCTCGAGTTCGCGAATACATATGGATTGGCGAGGATGGCCTGAAAATTCAGTCTTTTGGTAGCCAATTATGGGATACATCCTTATCGCTACATGTCTTGTTAGACACGCTCGTTAAAGGATATGATTACTTGAAGAAATCTCAAATTACAGAGAACCCTCGCGGTGATCACTTCCAAATGTTTCGTCACATTACAAAAGGTGGATGGACGTTTTCAGATCAAGATCAAGGATGGCCTGTTTCAGATTGTACTGCTGAAAGTTTAGAGTGTTGTCTATTCTTCGAGAGCTTGCCATCAGAGCTAATCGGGAAAAAAATGGATGTGGGGAAACTGTATGATGCTGTTGATTATCTTCTCTATCTGCAGAGTGATAATGGAGGCATAGCAGCATGGCAACCGGTTGATGGGAAAGCCTGGTTAGAGTGGCTTAGTCCAGTGGAGTTTCTTGAAGACGCGGTGGTCGAGTATGAGTATGTAGAATGTACGGGGTCAGCGATTGTCGCGTTGGCTAAGTTTAATAAACAGTTTCCAGAGTATAAAAAAGCAGAGGTTAAACAGTTTATAACAAAGGGTGCAAAGTACATTGAAGACATGCAAACGGTGGATGGTTCATGGTACGGAAATTGGGGAGTGTGTTTTATATATGGGACCTTCTTTGCGGTAAGAGGTCTTGTGGCTGCTGGGAAGACTTACGGTAACTGTGAAGCAATTCGTAAAGCAGTTCGTTTTCTTCTCGACACACAAAATCTGGAGGGTGGATGGGGAGAGAGCTTTCTCTCTTGTCCAAACAAGATATACACTCCTTTGAAAGGAAACAGCACAAATGTGGTGCAAACAGGACAAGCACTTATAGTGCTAATTATGGCTGATCAAATGGAGAGAGATCCTTTACCGGTTCATCGCGCTGCCAAAGTGTTGATCAATTCACAGTTGGATAATGGCGATTTTCCACAACAGGAAATAATGGGAACGTTCATGAGAACTGTGATGCTCCATTTTCCAACCTATAGGAACACGTTCTCTCTTTGGGCTCTCACACATTACACA

>AT5G48010

ATGTGGAGGCTGAGAACTGGACCGAAGACTCACCTGTTCACCACCAACAACTATGCAGGGAGGCAGATTTGGGAATTTGATGCCAACGCAGGCTCTCCACAAGAAATTGCCGAGGTAGAGGATGCTCGGCACAAATTCTCAGACAACACGTCACGTTTCAAGACTGCCGATCTCTTATGGCGCATGCAGTTTCTTAGGGAGAAGAAATTCGAACAGAAGATTCCAGTGATAATCGAGGATAAGTACGAAGATGCAAAGACAGCATTGAAAAGAGGGTTACTCTATTTCACAGCCTTGCAGGCTGATGATGGACACTGGCCAGCTGAAAACTCTGGCCCAAATTTCTATACCCCTCCTTTTTTGATATGCTTGTACATCACTGGACATCTGGAGAAAATCTTCACTCCCGAGCATGTTAAAGAGTTACTACGTCACATCTACAACAACGAAGATGGTGGGTGGGGTTTACACGTAGAAAGCCACAGTGTTATGTTCTGTACAGTCATTAATTACGTCTGTCTACGAATTGTGGGAGAAGAAGTCGGTTGTGCAAAGGCTCATAAGTGGATCATGGACCATGGTGGTGCTACCTACACGCCCTTGATCGGAAAAGCGTTGCTTTCGGTTCTTGGAGTGTATGATTGGTCTGGCTGCAATCCTATACCTCCAGAGTTCTGGTTGCTTCCGTCTTCTTTTCCTGTTAATATTTATTTACGGGATACTTTCATGGGGTTGTCATACTTGTATGGTAAAAAATTTGTGGCTCCCCCAACACCTCTCATTCTCCAGCTCCGAGAAGAGCTTTATCCGGAGCCTTATGCAAAAATCAATTGGACGCAAACACGAAACCGATGTGGAAAGGAAGATCTCTACTATCCACGCTCATTTTTACAAGATTTGTTTTGGAAGAGTGTTCACATGTTCTCAGAGAGTATCCTAGATCGATGGCCTTTAAACAAGCTAATAAGACAAAGAGCTCTTCAATCCACTATGGCACTCATTCACTATCATGACGAATCCACCAGATATATTACAGGCGGATGCCTGCCAAAGGCCTTTCATATGCTTGCATGTTGGATAGAAGACCCTAAGAGTGATTATTTTAAAAAACATCTTGCTCGAGTTCGCGAATACATATGGATTGGCGAGGATGGCCTGAAAATTCAATCTTTTGGTAGCCAATTATGGGATACAGCCTTATCGCTACATGCATTACTAGACACGCTCGTTAAAGGATATGATTACTTGAAGAAATCACAAATTACAGAGAACCCTCGCGGTGATCACTTCAAAATGTTTCGTCACAAGACAAAAGGTGGATGGACATTTTCAGATCAAGATCAAGGATGGCCTGTTTCAGATTGTACTGCTGAAAGCTTAGAGTGTTGTCTATTCTTCGAGAGCATGCCGTCCGAGCTTATTGGAAAAAAAATGGATGTGGAGAAACTCTATGATGCCGTTGATTATCTTCTCTATCTGCAGAGTGATAATGGAGGCATAGCAGCATGGCAACCAGTTGAAGGAAAAGCCTGGTTAGAGTGGCTTAGTCCAGTGGAGTTCCTTGAAGACACAATCGTGGAGTATGAGTATGTAGAATGTACGGGGTCAGCGATTGCAGCATTGACTCAGTTTAACAAACAGTTTCCAGGGTATAAAAACGTAGAGGTTAAACGGTTTATAACAAAGGCTGCAAAGTACATTGAAGACATGCAAACGGTGGATGGTTCATGGTACGGAAATTGGGGAGTGTGTTTTATATACGGGACCTTCTTTGCGGTAAGAGGTCTTGTGGCCGCTGGGAAGACTTACAGTAACTGTGAAGCAATTCGTAAAGCAGTTCGTTTTCTTCTAGACACACAAAATCCGGAGGGTGGCTGGGGAGAGAGCTTTCTCTCTTGTCCAAGCAAGAAATATACTCCTTTGAAAGGAAACAGCACAAATGTGGTGCAAACAGCACAAGCACTTATGGTGCTAATTATGGGTGATCAGATGGAGAGAGATCCTTTACCGGTTCATCGTGCTGCTCAAGTGTTGATCAATTCACAGTTGGATAATGGCGATTTTCCACAGCAGGAAATAATGGGAACGTTCATGAGAACTGTGATGCTCCATTTTCCGACCTATAGGAACACGTTCTCTCTTTGGGCTCTCACACATTACACA

>AT4G15340

ATGTGGAGACTAAGAATTGGAGCTAAGACTCACTTGTTCACGACCAACAACTATGTTGGGAGGCAGATTTGGGAGTTTGATGCCAACGCAGGCTCCCCTCAAGAACTTGCCGAGGTCGAGGAGGCTCGTCGGAATTTCTCTAACAATAGGTCACATTACAAGAGTGCTGATCTCTTATGGAGGATGCAGTTTCTTAGGGAAAAAGGTTTTGAACAGAAGATTCCAGTGAGAGTAGAGGATAGGTATGAAGATGCAAAAACCGCATTGAAAAGAGGATTACATTATTTTACGGCCTTGCAGGCAGATGATGGACACTGGCCTGCAGATAACTCTGGTCCAAATTTCTTCATCGCTCCTTTAGTTATATGCTTGTACATTACTGGACATCTGGAGAAAATCTTTACAGTCGAGCATCGTATAGAGTTAATACGTTACATGTATAACAACGAAGATGGTGGGTGGGGATTACATGTAGAAAGCCCTAGTATTATGTTTTGTACAGTCATCAACTACATTTGTTTACGAATCGTGGGAGTAGAAGCCACTTGTACAAAGGCTCGTAAGTGGATACTCGACCACGGTGGTGCTACCTACACACCCTTGATCGGAAAAGCTTGCCTATCAGTTCTTGGAGTGTATGATTGGTCTGGCTGCAAACCTATGCCGCCAGAGTTCTGGTTCCTTCCTTCTTCTTTTCCTATTAATATTTATTTACGGGATATTTTCATGGGGTTGTCATACTTGTATGGTAAAAAATTTGTTGCAACTCCAACACCTCTCATCCTCCAGCTCCAAGAAGAGCTTTATCCAGAGCCTTACACCAAAATCAATTGGAGGCTTACCAGAAACCGATGTGCAAAGGAAGATCTCTGTTATCCATCCTCATTTTTACAAGATTTGTTTTGGAAAGGTGTTCATATCTTCTCAGAGAGTATTCTAAATCGATGGCCTTTCAACAAGCTCATAAGACAAGCAGCTCTTCGAACCACTATGAAACTCCTTCACTATCAAGATGAAGCCAATAGATACATTACAGGCGGATCAGTGCCAAAGGCCTTTCATATGCTTGCATGTTGGGTAGAAGACCCAGAGGGTGAATATTTTAAAAAGCATCTTGCTCGAGTCTCTGATTTCATATGGATTGGCGAGGATGGCCTCAAAATTCAGTCTTTTGGCAGCCAATTGTGGGATACAGTCATGTCGCTACATTTCCTGTTAGACACGCTCGTTAAAGGATACGATTACTTGAAGAAATCTCAAGTTACAGAGAATCCTCCTAGTGATCACATAAAAATGTTCCGCCATATTTCGAAAGGTGGATGGACGTTTTCGGACAAAGATCAAGGATGGCCTGTTTCAGACTGCACTGCTGAGAGTTTAAAATGCTGTCTATTATTTGAGAGGATGCCGTCGGAGTTTGTTGGCCAGAAAATGGATGTGGAGAAACTCTTTGATGCCGTTGATTTTCTTCTCTACTTGCAGAGTGATAACGGAGGTATAACAGCTTGGGAACCCGCGGATGGGAAAACCTGGTTAGAGTGGTTTAGTCCGGTGGAATTTGTTCAAGACACGGTCATCGAGCATGAGTATGTGGAATGTACAGGGTCAGCCATTGTAGCGTTGACTCAGTTTAGTAAACAATTCCCGGAGTTTAGAAAGAAAGAGGTTGAAAGGTTTATAACTAACGGAGTGAAATACATTGAGGATTTGCAAATGAAGGATGGTTCATGGTGCGGAAACTGGGGAGTGTGCTTTATCTATGGGACCTTATTTGCCGTAAGAGGTCTTGTGGCTGCAGGAAAGACTTTCCATAACTGTGAACCCATTCGTCGAGCAGTTCGTTTCCTTCTCGACACGCAAAACCAGGAGGGTGGTTGGGGAGAGAGCTATCTCTCTTGCCTAAGGAAGAAATATACTCCTTTAGCAGGAAACAAGACAAATATAGTGAGTACAGGACAAGCGCTTATGGTTCTAATTATGGGTGGTCAGATGGAGAGAGATCCTTTGCCGGTTCATCGTGCTGCCAAAGTAGTGATCAATTTACAGTTGGATAACGGTGATTTCCCGCAACAGGAAGTAATGGGAGTATTCAATATGAATGTGCTGCTCCATTATCCAACATATAGGAACATTTATTCTCTTTGGGCCCTCACACTATACACG

>AT4G15370

ATGTGGAGACTGAGAATTGGTGCTAAGACTCACTTGTTCACCACCAATAACTATGTTGGTAGGCAGATTTGGGAGTTTGATGCCAACGCTGGCTCTCCGGAGGAACTTGCCGAGGTCGAGGAGGCTCGTCGGAATTTCTCCAACAACAGGTCACGGTTCAAGAGTGCCGATCTCCTATGGAGGATGCAGTTTCTTAGGGAGAAGAAGTTTGAGCAGAAGATTCCGGTGATAGTGGAGGATACGTATGAAGATGCAAAGACTGCATTAAGAAGAGGATTACTATATTTCACGGCATTGCAAGCTGATGATGGACATTGGCCTGCTGAAAACGCTGGTTCCATATTCTTCAATGCCCCTTTTGTGATATGTCTGTACATCACTGGACATCTTGAGAAAATCTTCACTCACGAGCATCGTGTAGAGTTACTTCGGTACATGTACAACAACGAAGATGGTGGGTGGGGATTGCACGTAGAAAGCCCCAGTAATATGTTCTGCTCAGTCATAAACTACATATGTTTACGGATCTTGGGAGTAGAAGCTGCTTGTGCAAGGGCTCGTAAATGGATCCTCGACCATGGTGGTGCTACATACTCCCCCTTGATCGGAAAAGCTTGGCTTTCGGTTCTTGGGGTTTATGATTGGTCTGGCTGCAAACCTATACCCCCTGAGTTCTGGTTCCTTCCTTCATTTTTCCCTGTTAATATTTATTTAAGGGATATTTTCATGGGGCTGTCATACTTGTATGGTAAAAACTTTGTCGCTACCTCAACACCTCTCATTCTCCAGCTCCGAGAAGAAATTTATCCGGAGCCTTACACAAATATCAGTTGGAGGCAAGCACGAAACCGATGTGCAAAGGAAGATCTCTATTATCCACAGTCATTTTTACAAGATTTGTTTTGGAAAGGTGTTCATGTATTCTCAGAGAATATCCTAAATCGATGGCCTTTCAACAATCTCATAAGACAAAGAGCTCTTCGAACCACTATGGAACTTGTTCACTATCATGATGAAGCAACCAGATATATTACAGGCGGATCTGTACCAAAGGTATTTCATATGCTTGCATGTTGGGTAGAAGACCCAGAGAGTGATTATTTTAAGAAACATCTTGCTCGAGTCCCTGATTTCATATGGATTGGCGAGGATGGACTGAAAATTCAATCTTTTGGTAGCCAAGTGTGGGATACAGCCTTGTCTTTACATGTCTTTATAGACACGCTCCTTAAAGGATATGATTACTTGGAAAAATCTCAAGTTACAGAGAACCCTCCTGGCGATTACATGAAAATGTTTCGTCACATGGCGAAAGGTGGATGGACGTTTTCGGACCAAGACCAAGGATGGCCAGTTTCAGATTGTACTGCTGAGAGTTTAGAGTGCTGTCTGTTCTTTGAGAGCATGTCGTCAGAGTTTATTGGCAAGAAAATGGATGTGGAGAAACTCTATGATGCAGTTGATTTCCTTCTCTATCTGCAGAGTGATAATGGAGGTATAACAGCATGGCAACCAGCGGACGGAAAACTT---------------------GTGGAGTTTATTGAAGACGCGGTTGTTGAGCATGAGTATGTAGAATGTACTGGATCTGCAATTGTAGCGTTGGCTCAATTCAATAAACAATTTCCGGGGTATAAAAAGGAAGAGGTTGAACGGTTTATAACAAAAGGCGTAAAGTACATTGAGGACTTGCAAATGGTGGATGGTTCATGGTACGGAAATTGGGGAGTGTGCTTCATCTATGGTACCTTCTTTGCTGTAAGAGGTCTTGTGGCTGCTGGGAAGTGTTACAATAACTGTGAGGCAATTCGTAGAGCAGTTCGTTTCATTCTCGACACTCAAAACACGGAGGGTGGCTGGGGAGAGAGCTATCTATCTTGTCCAAGAAAGAAATATATTCCGTTGATAGGAAACAAGACAAATGTGGTGAATACAGGACAAGCACTTATGGTTCTAATTATGGGAAATCAAATGAAGAGAGATCCTTTGCCGGTTCATCGTGCTGCCAAAGTGTTGATCAATTCACAAATGGATAACGGCGATTTCCCACAACAGGAAATAATGGGAGTGTTCAAGATGAATGTGATGCTCCATTTTCCAACCTACAGAAACATGTTCACTCTTTGGGCTCTTACACATTACACG

>AT1G78500

ATGTGGAGGCTGAAGATCGGGGCTAAGACTCACTTATTCACCACCAACAACTACACCGGAAGGCAGACTTGGGAGTTTGATGCCGATGCATGCTCTCCAGAGGAACTCGCTGAGGTCGATGAAGCTCGTCAGAATTTCTCCATAAACCGATCACGTTTCAAGAGTGCTGATCTCCTTTGGCGAATGCAGTTCCTGAGGGAGAAGAAGTTCGAGCAGAAGATTCCGGTGGAGATAGGAGATACGTACAAAGACGCAAAGACGGCACTGAGAAGAGGGATACTCTATTTCAAGGCATTGCAAGCTGAGGATGGACATTGGCCTGCTGAAAACTCTGGTTGCTTGTTCTTCGAAGCTCCCTTTGTCATATGCTTGTACATCACTGGACATTTGGAGAAAATCTTGACTTTGGAACATCGCAAAGAACTATTGCGTTACATGTACAACAACGAAGATGGTGGGTGGGGAATACACGTGGAAGGCCAAAGCGCTATGTTCTGCACAGTCATCAACTACATTTGCCTACGGATTCTGGGAGTAGAAGCAGGTTGTGCAAGGGCTCGTAAGTGGATCCTCGATCACGGTGGTGCTACTTATACGCCCTTGATCGGAAAAGCTTGGTTATCGATTCTTGGAGTGTATGATTGGTCAGGTTGCAAACCAATACCGCCTGAGGTGTGGATGCTCCCTACTTTTTCGCCCTTCAACATTTATTTCCGGGACATTTTTATGGGAGTGTCATACTTGTATGGTAAGAAATTTGTAGCTACACCAACACCTCTCATTTTACAGCTTCGGGAAGAGCTTTATCCTCAACCTTATGACAAAATCTTATGGAGTCAAGCTCGCAATCAGTGCGCAAAGGAAGATCTCTACTATCCACAGTCATTTTTACAAGAAATGTTTTGGAAATGTGTTCATATATTGTCAGAGAATATCCTGAATCGATGGCCTTGCAACAAGCTTATCAGGCAAAAAGCTCTTCGAACCACAATGGAACTTCTTCATTATCAAGATGAAGCCAGCCGTTACTTTACCGGTGGATGTGTGCCTAAGCCGTTTCATATGCTTGCTTGTTGGGTAGAAGACCCCGACGGTGATTATTTTAAGAAACATCTTGCTCGAGTCCCGGATTACATATGGATTGGGGAGGATGGTCTGAAAATCCAATCTTTTGGTAGCCAACTGTGGGATACAGCATTCTCACTTCAAGTCATGTTAGCTACGTTAATCAAAGGATACAGTTTCTTGAATAAATCTCAGCTTACACAAAATCCTCCTGGTGACCATAGAAAAATGTTGAAAGACATTGCGAAAGGAGGATGGACATTTTCGGACCAAGACCAAGGATGGCCTGTTTCGGATTGTACTGCTGAGAGTTTAGAGTGTTGCCTTGTCTTTGGAAGCATGCCATCGGAGTTGATTGGCGAGAAAATGGATGTAGAGAGGCTCTATGATGCCGTTAATTTACTTCTCTATTTTCAGAGCAAAAATGGAGGTATAACAGTGTGGGAGGCAGCTCGTGGAAGAACATGGTTAGAGTGGCTTAGTCCAGTGGAGTTTATGGAAGACACAATCGTCGAGCATGAGTATGTAGAATGTACGGGGTCAGCGATAGTGGCATTGGCTCGGTTCTTGAAGGAGTTTCCGGAGCACAGAAGGGAAGAGGTTGAAAAGTTTATCAAGAATGCTGTCAAATACATAGAAAGCTTTCAAATGCCCGATGGTTCGTGGTACGGAAACTGGGGTGTGTGTTTCATGTATGGAACCTTTTTCGCGGTAAGGGGTCTAGTGGCTGCAGGCAAGACGTACCAGAACTGTGAGCCGATTCGTAAAGCGGTTCAGTTCATTCTGGAGACACAAAACGTTGAAGGCGGTTGGGGTGAGAGTTATCTCTCTTGCCCCAACAAGAAATATACACTTCTAGAGGGAAACAGAACCAATGTGGTGAATACTGGACAAGCATTGATGGTTCTTATTATGGGTGGTCAGATGGAGAGAGACCCTTTGCCTGTTCATCGCGCAGCTAAAGTGTTGATCAATTCTCAGTTGGATAATGGCGATTTTCCGCAAGAGGAAATAATGGGAGTGTTCAAGATGAACGTGATGGTCCATTATGCAACCTATAGAAACATTTTCACTCTTTGGGCACTCACATACTACACA

>AL2G38900

ATGTGGAGGCTGAAGATCGGAGCTAAGACTCACTTGTTCACCACCAACAACTACACCGGAAGACAAACTTGGGAGTTTGATGCCGATGCCTGCTCCCCAGAGGAACTCGCTGAGGTCAATGAAGCTCGGCAGAATTTCTTCATAAACCGGTCACGTTTCAAGAGTGCTGATCTCCTTTGGCGAATGCAGTTTCTAAGGGAGAAGAAGTTCGAGCAGAAGATTCCGGTGGAAATAGGAGATACGTACAAAGACGCAAAGTCGGCACTGAGAAGAGGGATACTATATTTCAAGGCATTGCAAGCTGAGGATGGACATTGGCCTGCTGAAAACTCTGGTTCCTTGTTCTTCGAAGCTCCCTTTGTCATATGCTTGTACATTACTGGACATCTGGAGAAAATCTTCAGTTTGGAACATCGCAAGGAACTATTGCGCTACATGTACAACAACGAAGATGGTGGGTGGGGACTACACGTGGAAGGCCAAAGTGCTATGTTCTGCACGGTCATCAACTACATTTGCCTACGGATTTTTGGAGTAGAAGCAGGTTGTGCAAGGGCTCGTAAGTGGATCCTTGATCACGGTGGTGCTACATATACGCCCTTGATCGGAAAAGTTTGGCTATCGGTCCTTGGAGTGTATGATTGGTCCGGTTGCAAACCCATACCGCCTGAGGTGTGGATGCTCCCTTCTTTTTCGCCCTTCAACATTTATTTCCGGGAGATTTTCATGGGCGTGTCATACTTGTATGGTAAGAAATTTGTAGCTACACCAACACCTCTCATTTTACAGCTTCGGGAAGAGCTTTATCCTCAACCTTATGACAAAATCCTATGGAGTCAAGCTCGGAATATGTGCGCAAAGGAAGATCTCTACTATCCACAGTCATTTTTACAAGAAATGTTTTGGAAAGGTGTTCATACATTGTCAGAGACTATCCTGAGTCGATGGCCTTTCAACAAGCTCATAAGGCAAAAAGCTCTTCGAACCACAATGGAGCTCCTACATTATCACGATGAAGCGAGCCGATACTTTACCGGTGGATGTGTGCCAAAGCCGTTTCATATGCTTGCTTGTTGGGTAGAAGACCCCGACGGTGATTATTTTAAGAAACATCTTGCTCGAGTCCCCGATTATATATGGATTGGAGAGGATGGTCTGAAAATCCAATCTTTTGGTAGCCAATTGTGGGATACAGCATTCTCACTTCAAGTCATGTTAGCTACGTTAATGAAAGGATACAATTTCTTGAATAAATCTCAGCTTACACAAAATCCTCCTGGTGACCATAGGAAAATGTTTAAAGACATTGTGAAAGGAGGATGGACATTTTCGGACCAAGACCAAGGATGGCCTGTTTCCGATTGTACTGCTGAGAGTTTAGAGTGTTGCCTTGTCTTTGGAAGCATGCCATCGGAGTTGATTGGCGAGAAAATGGATGTGGAGAGGCTCTATGATGCCGTTAACTTACTTCTCTATTTTCAGAGCAAAAATGGAGGCATAGCAGTGTGGGAGGCAGCTCGTGGAAGAACCTGGCTAGAGTGGCTTAGTCCGGTGGAGTTTATGGAAGACACAATCGTCGAGCATGAGTATTTAGAATGCACGGGGTCTGCGATAGTGGCATTGGCACGGTTCTTGAAAGAGTATCCGGAGCACAGAAAGGAAGAGGTTGAAAGGTTTATCAAGAGTGCTGTCAAATACATAGAAAGCTTTCAAATGCCCGATGGTTCGTGGTATGGAAACTGGGGTGTCTGTTTCATGTATGGAACCTTTTTCGCGGTAAGAGGTCTAGTGGCTGCAGGCAAGACGTACCAGAACTGTGAGCCGATTCGTAGAGCGGTTCAGTTCCTTCTGGAGACACAAAACGTTCAAGGCGGTTGGGGTGAGAGTTATCTATCTTGCCCCAGGAAGAGATATACACCTCTAGAGGGAAACAGAACCAATGTGGTGAATACGGGACAAGCAATGATGGTTTTGATTATGTGTGGTCAGATGGAGAGAGACCCTTTGCCTGTTCATCGCGCAGCTAAAGTGTTGATCAATTCCCAGATGGATAATGGCGATTTTCCGCAAGAGGAAATAATGGGAGTGTTCAAGATGAATGTGATGATCAATTATCCAAACTATAGAAACATTTTCACTCTTTGGGCGCTCGCATACTACATG

>AL2G38860

ATGTGGAGACTAAGAATTGCAGCTGAAAATCACTTGTTCACAACCAACAGTTATGCCGGTAGACAGATTTGGGAATTTGATGCTAAGGCAGGCTCGCCTGAGGAATTAGCTGAAGTCAATAAGGTTCGACAGAAGTTCTCAAGCAATCCGCCTCGTTTCAAGAGTGCTGATCTACTTTGGCGCATGCAGTTTCTAAGGGAGAAGAGTTTTGAGCAGAAGATTCCGGTGATAATAGATGATACATATGAAGACGCCAAGATGGCACTTAAAAGAGGGATACTCTATTTTGCGGCCTTGCAAGCCGATGATGGACACTGGCCTGCTGAAAATTCTGGTCCCCTGTTCTTCGAAGCTCCCTTTGTCATAAGCTTGTATATCACTGGGCATCTGGATAAAATCTTCTCTCAAGAGCATCGCAAAGAGCTGATGCGTTACATGTACAACAACGATGATGGTGGATGGGGATTACATGTGGAAGGCCACAGTGTTTGGTTCTGTACGGTTATCAACTACATATGTCTAAGAATTTTTGGGATAGAGCCTGTTTGTGCAAGGGCTCGAAAGTGGATCCTCGACCACGGTGGTGCTACTTATACACCTTTGATCGGAAAGATTTGGCTTTCGGTTCTTGGAGTGTATGATTGGTCTGGTTGCAAACCCATACCACCGGAGTTTTGGATGCTCCCTTCTTCTTCTCCTGTCAATATTTATTTCCGGGACATTTTTATGGCTGTGTCATACTTGTATGGCAAAAAATTTGTCACTAAAACAACACCTCTAATTGCACAGCTCCGGGAAGAGCTTTATCCTCAGCGTTATAGCAAAATTGATTGGAGCCAAGCTCGCCAACAATGCGCAAAGGAAGATCTATACTATCCACAATCATTTGTACAAAACTTGTTTTGGAGAGGTGTTCATATGTTGTCAGAGAATGTCTTGAATCAGTGGCCTTTCAACAAACTCATAAGACAAAGAGCGCTCCGGAAAACAATGGAACTCATTCACTACCATAACGAAGTCACCAGATACATTACTGGTGGATGTGTGCAAAATGTATTTATTTCTTTGCATTTTACAAAAGAAGACCCAGAGAGTGATTACTTTAAGAAGCATCTCACTAGAGTCGCAGATTACATATGGATTGGAGAGGATGGTCTAAAAATCCAGTCGTTTGGTAGCCAGTTGTGGGATACGGCCCTATCACTCCAAGTCATGTTAGGTACGTTGGTCAAGGGATACAAATTCTTGGAGAATTCTCAGATAACACAGAATCCTCCCGGTGACCATATGAAAATGTTCCGACACATTACAAAAGGTGGATGGACTTTTTCAGACCAAGATCAAGGATGGCCTGTTTCTGATTGCACTGCTGAGAGTTTAGAGTGTTGCCTAATCTTTGAAAGCCAGCCGTTTGAGATCATTGGTGAGAAAATGGATGTCAAGAGACTTTATGACGCTGTCAATTTTCTTCTAAATCTGCAGAGCAAGAATGGAGGTATATCGGCGTGGGAGCCAGCCCTTGGGAAAACCTGGCTAGAGTGGCTCAGTCCAGTGGAGTTCATGGAAAACACAATCGTGGAGCACGAGTATGTAGAATGCACAGGATCAGTGATAATAGCATTGGCCAGGTTCAAGCAACAGTTTCCAGCTCACAGAACAGAAGAAGTTGAAAGGTTTATAGTAAAGGGAGTGAAATACATAGAGAACTTACAAATGGTGGATGGTTCATGGTATGGCAACTGGGGAGTGTGTTTCATGTATGGATCCTTTTTTGCTATAAGAGGTCTAGTGGCTGCAGGAAAGACTTATAGTAACTGCAAGGCGATTCGTAGAGCAGTTCAGTTCTTTCTCAACACACAAAACGTTGAAGGCGGTTGGGGAGAGAGTTTTCTCTCTTGCCCCAACAAGAAATATATTCCTTTAGAAGGAAACAAGAGTAATGTAGTGAATACAGGACAAGCACTAATGGGTCTAATTATGGGTGGTCAGATGGAGCGAAACCCGTTACCAGTTCACCGTGCTGCCAAAGTGTTGATCAATTTGCAGATGGATAACGGTGATTTTCCCCAAGAGGAAATGAGAGGAGTGTTCAAGATGAATGTGGTGCAACATTATCCAACATATAGGAACATATTCACTCTCTGGGCACTCACATATTATACT

>AL2G38880

ATGTGGAGGCTGAGAATTGCAGCTGAAACTCACTTGTTCACAACCAACAATTATGCCGGTAGGCAGATTTGGGAGTTTGATGCAAATACATGCTCTCCTGAGGAACTCGCTGAGGTCAATAAGGTTCGACAGAATTTCTCTAGCAACCCGTCTCGTTTCAAGAGTGCTGATCTACTTTGGCGCATGCAGTTTTTAAGGGAGAAGAAATTCGAGCAGAAGATTCCGGTGATAATAGAGGATACGTATGAAGACGCCAAGACAGCACTTAGAAGAGGGATACTTTATTTTGCGGCCTTGCAGGCTGATGATGGACATTGGCCTGCTGAAAATTCTGGCTCCTTGTTCTTCGAAGCTCCCTTTGTCATAAGCTTGTACATCACTGGGCACTTGGATAAAATCTTCTCATTGGAGCATCGTAAAGAGCTACTGCGTTACTTGTACAACAACGATGACGGTGGATGGGGAATACATGTGGAAAGCCAAAGTGTCATGTTCTGTACGGTCATCAACTACATCTGTCTAAGAATTTTTGGGATAGAACCTGTTTGTGCAAGAGCTCGAAAGTGGATCCTCGACCACGGCGGTGCCACCTATACGCCTTTGATCGGAAAGATTTGGCTTTCGGTTCTTGGAGTGTATGATTGGTCTGGTTGCAAACCGATACCGCCTGAGTTTTGGATGCTCCCTGGTTCTTCTCCCATCAATATTTATTTCCGGGACATCTTCATGGCCTTGTCATACTTGTATGGCAAAAAATTTGTTGCTAGCCCGACACCTCTAATTGCGCAGCTCCGTGAAGAGCTTTATCCTCAGCCTTACAACAAAATCAATTGGAGCCAAGCTCGTCAACTATGCGCAAAGGAAGATCTATACTATCCACAATCATTTGTGCAAGACTTGTTTTGGAAAAGTGTTTACATGTTCTCAGAAAATGTTTTAAACCAATGGCCTTTCAACAAACTCATAAGACAAAGAGCTCTCCGAAAAGCAATGGAACTCATTCACTACCATGACGAAGCCACCCGTTACATTACTGGTGGATGTGTGCAAAAGCCGTTTCATATGCTCGCTTGTTGGGTAGAAGACCCCGACGGTGATTATTTTAAGAAACATCTTGCTCGAGTACCCGATTACATATGGGTTGGGGAGGATGGTTTGAAAATCCAGTCTTTTGGTAGCCAGTTATGGGATACATCCCTATCGCTCCAAGTCATGTTAGCTACTTTGGTGAAGGGATACAAATTCTTGGAGAAATCTCAGCTTACACAAAATCCTCCTGGTGACCATATGAAAATGTTCCGACACATTACAAAAGGTGGATGGACTTTTTCAGACCAAGATCAAGGATGGCCTGTTTCTGATTGCACTGCTGAGAGTTTAGAGTGCTGCTTAATCTTTGAAAGCATGTCGTCTGAGATCATTGGTGAAAAAATGGATATAGAGAGACTCTATGATGCTGTCAATTTTCTTCTCTATTTGCAGAGCAAAAATGGAGGTATTTCAGCTTGGGAGCCAGCCCTTGGGAAAACGTGGCTAGAGTGGCTCAGTCCAGTAGAGTTTATGGAAAACACAACCATCGAGCATGAGTATGTAGAATGCACGGGTTCAGCGATAATAGCATTAGCTAGGTTCAAGCAACAATTTCCAAGGCATAGAACAGAAGAAGTTGAAAGATTTATAACAAAGGGAGTGAAATACATAGAAAGCTTTCAAATGCCGGATGGTTCATGGTATGGCAACTATGGAGTGTGTTTCATGTATGGAACTTTTTTTGCGGTAAGAGGTCTAGTGGCAGCAGGCAAGACTTATTGTAATAGTGAGCCGATTCATAGAGCGGTTCAGTTCCTTCTCGAGACACAAAACATTGAAGGCGGTTGGGGTGAGAGTTATCTCTCTTGCCCCAACAAAAAATATACTCCTTTAGAAGGAAACAAAACCAATGTGGTGAATACATCACAAGCACTGATGGGTCTAATTATGGGTGGTCAGATGAAGAGAGACCCTTTGCCGGTTCATCGTGCTGCGAAAGTGTTGATAAATTCGCAGTTGGATAATGGCGATTTTCCACAAGAGGAAATAAGAGGAGTGTTTAAGATGAATGTGCTGCTACATTATCCAACATATAGGAACATGTTCACTCTCTGGGCACTCACATATTATACT

>AT5G36150

ATGTGGAGGCTGAGGATCGGAGCTAAGCCTCACTTGTGCACCACCAACAACTTCTTGGGAAGGCAGATATGGGAGTTTGATGCCAACGCAGGCTCTCCAGCGGAACTCTCTGAGGTTGATCAGGCTCGACAAAATTTCTCAAACAATAGGTCACAATACAAGTGTGCCGATCTCCTTTGGCGTATGCAGTTTCTAAGGGAGAAGAATTTCGAGCAAAAGATTCCAGTGAGAATAGAGGATACATTTGAAGACGCAAAAAATACACTGAGAAGAGGAATACATTATATGGCAGCGTTGCAATCTGATGATGGACATTGGCCTTCCGAAAACGCTGGTTGCATTTTCTTCAATGCCCCCTTTGTTATATGTTTGTATATCACTGGCCATCTGGATAAAGTTTTCTCTGAAGAGCATCGGAAAGAGATGTTGCGTTACATGTACAACAACGACGATGGTGGATGGGGAATAGACGTAGAAAGCCATAGTTTTATGTTTTGCACGGTCATCAACTACATCTGCCTACGAATCTTCGGAGTAGATCCCGCTTGTGCAAGGGCTCGTAAATGGATCATTGACCACGGTGGCGCTACCTATACGCCATTATTTGGAAAAGCCTGGCTTTCGGTTCTTGGAGTATATGAATGGTCTGGTTGCAAACCCATACCCCCAGAGTTCTGGTTTTTTCCTTCCTATTTTCCTATTAATATATATTTACGGGATACTTTCATGGCAATGTCCTATTTGTATGGTAAAAAATTTGTTGCTAAACCAACACCTCTCATTCTACAACTTCGTGAAGAACTTTATCCTCAACCTTATGCTGAAATTGTTTGGAGCCAAGCTCGCAGTCGATGTGCGAAGGAAGATCTATATTATCCACAATCATTGGTACAAGACTTGTTTTGGAAACTTGTTCACATGTTTTCGGAGAATATCTTAAATCGATGGCCTTTCAACAAGCTCATTAGAGAAAAAGCTATTCGAACGGCAATGGAACTCATTCACTACCATGACGAAGCCACCCGGTACATTACAGGTGGAGCAGTGCCAAAGGTGTTTCATATGCTTGCTTGTTGGGTTGAAGATCCAGAGAGTGATTATTTTAAAAAACATCTTGCGCGAGTCTCTCATTTCATATGGATTGCGGAGGACGGCTTGAAAATCCAGACTTTTGGTAGCCAAATATGGGATACAGCCTTCGTTCTCCAAGTCATGTTAGCGACGCTCATAAAGGGATACTCTTACTTGAGGAAATCCCAATTTACAGAGAATCCTCCCGGTGACTATATCAATATGTTTAGAGACATATCCAAAGGAGGGTGGGGCTATTCAGACAAAGATCAAGGATGGCCTGTTTCAGATTGTATTTCTGAGAGTTTAGAGTGCTGCCTGATCTTTGAGAGTATGTCATCCGAATTCATTGGTGAGAAAATGGAAGTGGAGAGGCTTTATGATGCCGTCAATATGCTTCTCTATATGCAGAGCAGAAATGGAGGGATATCTATATGGGAAGCAGCGAGTGGGAAAAAATGGCTAGAGTGGCTTAGTCCCATAGAGTTTATTGAAGACACTATCCTCGAGCATGAGTATCTAGAATGCACGGGGTCAGCGATAGTGGTGTTGGCACGCTTCATGAAACAGTTTCCAGGGCATAGAACAGAAGAAGTCAAAAAATTTATAACAAAGGGAGTGAAATACATAGAAAGCTTACAAATTGCGGATGGTTCGTGGTACGGAAACTGGGGAATATGTTTTATATATGGGACTTTCTTTGCTGTCCGAGGTTTAGTGGCCGCGGGAAACACTTACGATAACTGTGAGGCAATCCGTAGAGCAGTTCGATTCCTTCTTGATATACAAAACGGTGAAGGCGGTTGGGGAGAGAGTTTTCTTTCTTGCCCCAACAAAAATTATATTCCTTTGGAGGGGAACAAGACCGATGTGGTGAATACAGGACAAGCATTGATGGTTCTAATAATGGGTGGTCAGATGGATAGAGATCCTTTACCGGTTCACCGCGCTGCAAAAGTATTAATCAATTCACAAATGGATAACGGTGATTTTCCACAGCAGGAAATAAGGGGTGTTTACAAAATGAATGTGATGCTAAATTTTCCAACCTTTAGAAACTCTTTCACTCTTTGGGCACTAACACACTACACC

>AL2G39400

ATGTGGAAGTTGAAGATAGCAAATGGACCCTACTTGTTCAGCACCAACAACTTCCTCGGAAGACAGACATGGGAGTTTGATCCGGACGCCGGCATAGCAGAGGAACTAGCTGCCGTCGAAGAAGCTCGTCGGAAATATTTTGATGATCGTTTTCAGGTTAAAAGCAGCGATCTCATATGGCGTATGCAGTTTTTAAAAGAGAAGAAATTCGAGCAAGTTATACCTGTAAAAGTTGAAGATACGAGCGAAATAGCTACAAATGCGTTAAGGAGAGGGGTCAATTTCTTATCGGCATTGCAGGCCACTGATGGACACTGGCCTGCAGAAAATGCTGGTCCTTTATTCTTCCTTCCTCCATTGGTTTTCTGTCTATTTGTCACTGGACATCTCCATGAGATATTCACTCAAGAGCATCGTCGAGAGATCCTCAGATACATCTACTGTAATGAAGATGGTGGCTGGGGATTACACATAGAAGGAGACAGCACCATGTTCTGCACCACACTAAACTATATTTGCATGCGCATACTTGGAGAATCTCCTGCGTGCAGACGGGCCAGGGATTGGATTCTTGACCATGGGGGTGCAACATACATACCCTCTTGGGGCAAAACTTGGCTTTCTATACTTGGTGTCTTTGATTGGTCAGGAAGCAACCCCATGCCTCCAGAGTTTTGGATCCTACCTTCGTTTCTTCCAATACATTGTTACTGCCGGTTGGTTTACATGCCAATGTCTTATCTTTACGGGAAGAGATTTGTTGGTCCAATAAGTCCTCTTATTCTGCAACTACGCGAAGAAATTTACTTGCAGCCTTATGCAAAAATAAACTGGAACAGAGCACGCCATCTATGTGCAAAGGAAGACGCGTACTGTCCTCATCCACAAATTCAAGATGTTATATGGGACTGTCTTTACATCTTCACTGAGCCGTTTCTTACATGTTGGCCATTTAATAAGCTGCTTAGGGAAAAAGCTCTTGGGGTGGCAATGAAACACATACATTATGAAGACGAAAATAGCCGTTATATTACCATTGGATGTGTTGAAAAGGCATTATGCATGCTTGCCTGTTGGGTTGAGGATCCTAACGGAAGTCATTTCAAGAAGCATCTTTTGAGGATTTCTGATTACTTGTGGATTGCAGAAGATGGGATGAAAATGCAGAGCTTTGGAAGTCAATTATGGGATTCAGGATTTGCCCTCCAAGCTTTAGTTGCAGTACTCAGGAGAGGATATGACTTTTTAAAAAATTCTCAGGTTAGGGAGAACCCTTCGGGTGACTTTACGAACATGTTCCGTCACATCTCTAAAGGGTCGTGGACTTTCTCTGATCGAGACCATGGATGGCAAGCTTCCGACTGCACAGCCGAAGGGTTTAAGTGTTGCCTTTTGCTTTCGATGATGCCACCTGACATTGTTGGCCCGAAAATGGATCCCGAACAGTTATATGAGGCTGTTACTATCTTACTGTCTCTACAGAGTAAAAATGGAGGTGTAACTGCTTGGGAGCCTGCCCGTGGACAAGAATGGTTGGAATTGCTAAATCCTACTGAAGTTTTTGCTGACATTGTGGTTGAGCACGAGTACAATGAGTGTACTTCATCAGCAATCCAAGCTTTGATTCTGTTCAAGCAACTATATCCGAATCACAGGACAGCAGAGATCAACACTTCCATCAAGAAAGCCGTGCAATATATAGAGAGCATACAAATGCATGATGGTTCATGGTACGGAAGCTGGGGAGTTTGCTTCACATACAGTACATGGTTTGGTCTGGGAGGCCTCGCAGCTGCTGGAAAGACGTACAACAACTGTTTGGCTATGCGTAAAGGCGTTCATTTCCTTCTCACAACTCAAAAAGATAATGGAGGTTGGGGTGAAAGCTACTTGTCATGTCCTAAAAAGAGATACATTCCAAGTGAAGGGGATAGATCAAACTTGGTGCAAACCTCTTGGGCAATGATGGGTCTACTTCATGCTGGACAGGCAGAGCGAGATCCGGCTCCTCTTCACCGTGCTGCGAAGCTCTTAATCAACTCTCAACTGGAGAATGGCGATTTTCCTCAGCAGGAGATAACTGGAGCTTTCATGAAGAACTGCTTGTTACACTATGCAGCATACAGAAACATCTTCCCCGTGTGGGCACTCGCAGAGTATAGG

>AT1G78955

ATGTGGAAGTTGAAGATAGCAAATGGACCCTACTTGTTCAGTACCAACAACTTCCTCGGAAGACAGACATGGGAGTTTGATCCGGACGCCGGCACAGTGGAGGAACTAGCTGCCGTCGAAGAAGCTCGTCGGAAATTTTATGATGATCGTTTTCGTGTTAAAAGCAGCGATCTCATATGGCGTATGCAGTTTTTAAAGGAGAAGAAATTCGAGCAAGTTATACCTGCGAAGGTTGAAGATACGAGCGAAATAGCTACAAATGCGTTAAGGAAAGGGGTTAATTTCTTATCAGCATTGCAGGCCAGTGATGGCCATTGGCCTGCTGAAAATGCTGGTCCTTTATTCTTCCTTCCTCCATTGGTTTTCTGTCTATACGTCACTGGACATCTCCATGAGATTTTCACTCAAGACCATCGTCGAGAGGTCCTTAGATACATCTACTGTAATGAAGATGGTGGGTGGGGATTACACATAGAAGGAAACAGCACCATGTTCTGCACCACACTAAACTACATTTGCATGCGCATACTTGGAGAAGGTCCAGCGTGCAAACGGGCCAGGGATTGGATTCTTGACCATGGTGGTGCAACATACATACCTTCTTGGGGCAAAACTTGGCTTTCGATACTTGGTGTCTTTGATTGGTCAGGAAGCAACCCCATGCCTCCAGAGTTTTGGATCCTACCTTCCTTTCTTCCAATACATTGTTACTGCCGGTTGGTTTACATGCCAATGTCTTATCTTTACGGGAAGAGATTCGTTGGTCCAATAAGTCCTCTTATTCTGCAACTACGCGAAGAAATTTACTTGCAGCCTTATGCGAAAATAAACTGGAATAGAGCTCGCCATCTATGTGCAAAGGAAGATGCGTACTGTCCTCATCCACAAATTCAAGATGTTATATGGAACTGCCTTTACATCTTCACCGAACCGTTTCTTGCATGTTGGCCATTTAACAAGCTGCTAAGGGAAAAAGCTCTTGGGGTGGCAATGAAACACATACATTATGAAGACGAAAATAGCCGTTATATTACCATTGGATGTGTTGAAAAGGCATTATGCATGCTTGCGTGTTGGGTTGAAGACCCTAACGGAATTCATTTCAAAAAGCATCTTTTGAGGATTTCTGATTACTTGTGGATTGCAGAAGATGGGATGAAAATGCAGAGCTTTGGAAGTCAATTATGGGATTCGGGGTTTGCCCTCCAAGCGTTAGTTGCAGTACTCAGGAGAGGATATGACTTTTTGAAAAATTCTCAGGTTAGAGAGAACCCTTCGGGTGACTTTACAAACATGTACCGTCACATATCCAAAGGGTCGTGGACTTTCTCTGATCGAGATCATGGATGGCAAGCTTCCGACTGCACAGCCGAAAGTTTCAAGTGTTGCCTGTTGCTTTCAATGATACCACCTGACATTGTTGGCCCGAAAATGGATCCCGAACAGTTATATGAGGCTGTTACTATCTTACTGTCTCTACAGAGTAAAAATGGAGGTGTAACTGCTTGGGAGCCTGCCCGTGGACAAGAATGGTTGGAATTGCTAAACCCTACTGAAGTTTTTGCTGACATTGTGGTTGAGCACGAGTACAATGAGTGTACTTCATCAGCGATCCAAGCTTTGATTCTTTTCAAGCAACTATATCCGAATCACAGGACAGAAGAGATCAACACTTCCATCAAGAAAGCCGTGCAATACATAGAGAGCATACAAATGCTTGATGGTTCATGGTACGGAAGCTGGGGAGTTTGCTTCACATACAGTACATGGTTTGGTCTGGGAGGCCTTGCAGCTGCTGGAAAGACATACAACAACTGCTTGGCTATGCGTAAAGGCGTTCATTTCCTTCTCACAACTCAAAAAGATAATGGAGGTTGGGGTGAAAGCTACTTGTCATGTCCTAAAAAGAGATACATTCCAAGTGAAGGAGAGCGGTCAAACTTGGTGCAAACCTCTTGGGCAATGATGGGTTTACTTCATGCTGGACAGGCCGAGCGAGATCCGTCTCCTCTTCACCGTGCTGCAAAGCTCTTAATCAACTCTCAACTGGAGAATGGCGATTTTCCTCAGCAGGAGATAACTGGAGCTTTCATGAAGAACTGCTTGTTACACTACGCAGCATACAGAAACATCTTCCCCGTGTGGGCACTCGCAGAGTATAGG

>AT1G78950

ATGTGGAGGTTGAAGATAGGAGAAGGGCCCTACTTATTCACTACTAACAACTTCGCCGGACGACAAACATGGGAGTTTGATCCTGACGGAGGCTCGCCGGAGGAAAGACACTCCGTCGTTGAGGCTCGCCGGATTTTCTACGACAATCGTTTTCATGTTAAAAGCAGCGATCTCTTGTGGCGCATGCAGTTTCTGAGGGAGAAAAAGTTTGAGCAACGGATAGCTGTGAAAGTTGAAGACACCTTTGAAACGGCGACGAGTGCATTACGGAGAGGTATTCATTTCTTCTCGGCGTTGCAGGCCAGCGACGGTCACTGGCCGGCGGAAAATGCCGGCCCTTTGTTTTTCCTTCCGCCACTGGTGTTTTGTCTTTACATAACGGGACATCTTGATGAAGTGTTCACTTCAGAACATCGAAAAGAGATTCTTCGATACATCTACTGTAAGGAAGATGGTGGGTGGGGATTACACATTGAAGGTCATAGCACAATGTTCTGTACCACGTTGAATTACATATGTATGCGTATACTTGGAGAAAGTCCTGCGTGTGGACGAGCCCGGGAATGGATCCTTAGTCATGGTGGTGTCACCTACATACCTTCTTGGGGTAAAACTTGGCTTTCGATACTTGGTGTATTTGACTGGTCTGGAAGCAACCCAATGCCTCCTGAGTTTTGGATCCTACCTTCTTTCTTTCCCGTGCATAGCTACTGCCGGATGGTTTACTTGCCAATGTCGTATCTCTACGGGAAGCGGTTTGTTGGCCCAATAACGTCTCTGATTTTGCAACTACGCAAAGAACTGTACTTACAGCCTTATGAAGAAATCAATTGGATGAAAGTCCGCCATCTTTGTGCAAAGGAAGATACATATTATCCCCGTCCACTGGTTCAAGAGTTGGTATGGGACAGTCTTTACATCTTTGCGGAGCCTTTCCTTGCACGTTGGCCATTTAACAAGCTTCTTAGGGAAAAGGCTCTTCAATTGGCAATGAAACACATACACTATGAAGATGAAAATAGTCGTTATATCACCATTGGGTGTGTTGAAAAGGTACTATGCATGCTAGCTTGTTGGGTTGAAGACCCGAATGGGGATTATTTCAAGAAGCACCTCTCTAGAATCTCCGATTACTTGTGGATGGCTGAAGATGGGATGAAAATGCAGAGCTTTGGAAGTCAACTATGGGATACAGGGTTTGCGATGCAAGCTTTACTTGCAGTACTCAGGAGAGGACATGAGTTCATAAAGAATTCACAGGTTGGAGAGAACCCTTCAGGTGATTACAAAAGCATGTACCGTCATATTTCAAAAGGAGCATGGACTTTTTCTGACCGAGATCACGGATGGCAAGTTTCAGACTGCACAGCTCATGGCTTAAAGTGCTGCCTGCTGTTCTCGATGTTGGCGCCGGATATTGTTGGCCCAAAACAAGACCCCGAGAGACTACATGATTCTGTTAATATCTTGCTCTCTTTACAGAGCAAAAACGGAGGTATGACTGCCTGGGAGCCTGCTGGTGCTCCCAAGTGGTTGGAATTGCTCAATCCCACAGAAATGTTTTCCGACATTGTGATTGAGCATGAGTACAGTGAATGTACATCGTCTGCAATCCAAGCATTGAGTCTTTTCAAGCAACTCTACCCTGATCACAGGACAACAGAGATCACCGCTTTCATCAAGAAAGCTGCGGAATACCTAGAAAACATGCAAACACGTGATGGTTCATGGTATGGGAACTGGGGCATTTGCTTCACGTACGGTACATGGTTTGCTCTTGCAGGCTTAGCAGCTGCGGGTAAGACTTTTAACGACTGTGAGGCAATACGCAAAGGCGTTCAGTTTCTTCTTGCAGCTCAGAAAGACAACGGAGGCTGGGGAGAAAGCTACCTCTCTTGCTCCAAAAAGATATACATAGCACAAGTAGGGGAGATATCAAACGTGGTGCAAACTGCTTGGGCTTTAATGGGTCTCATTCACTCTGGACAGGCAGAGAGAGATCCGATTCCTCTTCACCGTGCTGCGAAACTTATCATCAATTCACAACTGGAGAGTGGAGATTTTCCTCAACAGCAAGCAACCGGAGTGTTTTTGAAGAATTGCACATTACACTACGCTGCATATAGAAACATTCATCCGTTGTGGGCACTCGCAGAATATCGC

>AL2G39420

ATGTGGAAGTTGAAGATAGGAAAGGGACCGCATTTATTCAGTAGCAATAATTTCGTCGGACGTCAAACATGGGAGTTTGATCACCAAGCCGGCTCACCAGAGGAAAGAGCTTCCGTCGAAGAAGCTCGCCGAAGTTTCTTGATCAACCGTTCTCGTGTTAAATGCAGTGATCTCTTATGGCGAATGCAATTTCTAAGAGAGAAGAAATTCGAACAAGGCATACCGACTAAAATAAAG---ACGTATGAAACAACGACAAATGCATTACGAAGAGGCGTTCGTTACTTCTCAGCTTTGCAAGCCTCCGACGGCCATTGGCCTGGAGAAATCACTGGTCCGCTTTTCTTCCTTCCTCCTCTCATATTTTGTTTGTACATTACCGGACATCTGGAGGAAGTATTCGATGCTGAACATCGCAAAGAGATGCTACGGCATATCTATTGTAACGAAGATGGTGGATGGGGATCACATATCGAAAGCAAGAGTGTTATGTTCTGCACCGTGTTGAATTACATATGTTTTCGTATGCTTGGAGAAAATCCTGCGTGCAAACGAGCTAGACAATGGATTCTTGACCGTGGTGGTGTGATTTTTATTCCTTCTTGGGGGAAATTTTGGCTCTCGATACTCGGAGTTTATGAATGGTCTGGAACTAATCCGACGCCACCAGAAATCTTGATGCTGCCTTCTTTTCTTCCAATACATTGTTATAGCCGGATGGTTAGTATACCTATGTCTTACCTATATGGGAAGAGGTTTGTTGGTCCAATAACACCTCTTATTTTGCTCTTGCGCGGGGAACTTTACTTGGAATCTTATGAAGAAATCAGTTGGAATAAAACTCGACGTCTATATGCAAAAGAAGACATGTATTATCCTCATCCTTTGGTTCAAGATTTGATATCTGACACTCTTCACAACTTTGTGGAGCCTTTTCTTACACGTTGGCCATTGAACAAGCTTGTGAGGGAAAAAGCTCTTCAGCTGACTATGAAACACATACATTATGAAGACGAAAATAGCCATTACATAACCATTGGATGTGTTGAGAAGGTACTGTGCATGCTAGCTTGTTGGGTCGAAAATCCTAATGGAGACTACTTCAAGAAGCATCTGGCTAGAATTCCAGATTACATGTGGGTCGCTGAAGATGGAATGAAAATGCAGAGCTTTGGATGTCAACTGTGGGATACCGGATTTGCTATTCAAGCTTTACTTGCAGCATTAAGGAGAGGACATAATTACATCAAGACATCTCAGGTTAGAGAAAACCCTTCAGGTGATTTTAAGAGCATGTACCGCCACATTTCGAAAGGAGCATGGACATTTTCTGATCGAGATCATGGATGGCAAGTTTCAGATTGTACAGCTGAAGCTTTAAAGTGTTGCCTACTTCTTTCCATGATGCCAGCTGATATCGTTGGCCAGATAATAGATGATGAACAATTATATGATTCTGTTAATCTCTTGCTATCTTTACAGAGCGGAAATGGAGGTGTCAATGCGTGGGAGCCTACCCGTGCATATGAATGGATGGAACTGCTCAATCCTACAGAATTCATGGCTAATACCATGGTCGAGCGGAAGTTTGTGGAATGCACCTCATCTGTTATACAAGCACTTGATCTATTTAGAAAGTTGTATCCAGATCACAGGACAAAAGAGATCAACAAGTCCATCAAAAAAGCTGTGCAATTTATACAAGGCAAACAAACAGCAGACGGTTCATGGTACGGAAATTGGGGTGTTTGCTTCATTTACGCTACTTGGTTTGCTCTTGGAGGTCTAGCAGCAGCTGGTGAAACTTACAACGATTGTTTAGCTATGCGCAAAGGTGTCCACTTTTTGCTCACTACACAAAGAGATGATGGAGGTTGGGGTGAAAGCTATTTATCATGCTCCGAACAGAGATACATACCATTAGAAGGAGAAAGATCAAACATTGTGCAAACATCATGGGCTATGATGGCTCTAATTCATACGGGACAGGCTGAGAGAGATTTGATTCCTCTTCATCGTGCTGCCAAACTAATCATCAATTCACAACTTGAAAACGGGGATTTTCCTCAACAGGAAATAGTAGGAGCGTTCATGAATACATGCATGCTACACTATGCTACATACCGAAACACCTTCCCCTTATGGGCACTCGCGGAATACCGA

>AT1G78970

ATGTGGAAGTTGAAGATAGGAAAGGGACCGCATTTATTCAGCAGCAATAACTTCGTCGGACGTCAAACATGGAAGTTTGATCACAAAGCCGGCTCACCGGAGGAACGAGCTGCCGTCGAAGAAGCTCGCCGGGGTTTCTTGGATAACCGTTTTCGTGTTAAATGCAGTGATCTATTGTGGCGAATGCAATTTCTAAGAGAGAAGAAATTCGAACAAGGCATACCACTAAAAGCTACTAACACGTATGAAACAACGACAAATGCATTACGAAGAGGCGTTCGTTACTTCACGGCTTTGCAAGCCTCCGACGGCCATTGGCCGGGAGAAATCACCGGTCCGCTTTTCTTCCTTCCTCCTCTCATATTTTGTTTGTACATTACCGGACATCTGGAGGAAGTATTCGATGCTGAACATCGCAAAGAGATGCTAAGACATATCTATTGTAACGAAGATGGTGGATGGGGATTACACATCGAAAGCAAGAGTGTTATGTTCTGCACCGTGTTGAATTACATATGTTTACGTATGCTTGGAGAAAATCCTGCATGCAAACGAGCTAGACAATGGATTCTTGACCGCGGTGGAGTGATCTTTATTCCTTCTTGGGGGAAATTTTGGCTCTCGATACTTGGAGTCTATGATTGGTCTGGAACTAATCCGACGCCACCAGAACTCTTGATGCTGCCTTCTTTTCTTCCAATACATTGTTATAGCCGGATGGTTAGTATACCTATGTCGTATCTATATGGGAAGAGGTTTGTTGGTCCAATTACACCTCTTATTTTACTCTTGCGCGAAGAACTTTACTTGGAACCTTATGAAGAAATCAATTGGAAAAAAAGTCGACGTCTATATGCAAAAGAAGACATGTATTATGCTCATCCTTTGGTTCAAGATTTGTTATCTGACACTCTTCAAAACTTTGTGGAGCCTTTACTTACACGTTGGCCATTGAACAAGCTTGTGAGGGAAAAAGCTCTTCAGCTTACTATGAAACACATACACTATGAAGACGAAAATAGCCATTACATAACCATTGGATGTGTTGAAAAGGTACTGTGCATGCTAGCTTGTTGGGTTGAAAATCCGAATGGAGATTATTTCAAGAAGCATCTGGCTAGAATTCCAGATTATATGTGGGTCGCTGAAGATGGAATGAAAATGCAGAGCTTTGGATGTCAACTGTGGGATACTGGATTTGCTATTCAAGCTTTGCTTGCAGCACTAAAGAGAGGACATAATTACATAAAGGCATCTCAGGTTAGAGAAAACCCTTCAGGTGATTTTAGGAGCATGTACCGCCACATTTCGAAAGGAGCATGGACATTTTCTGATCGAGATCATGGATGGCAAGTTTCAGATTGTACAGCTGAAGCTTTAAAGTGTTGCCTGCTGCTTTCCATGATGTCAGCTGATATCGTCGGCCAGAAAATAGATGATGAACAATTATATGACTCTGTTAACCTCTTGCTGTCTTTACAGAGCGGAAATGGAGGTGTCAATGCGTGGGAGCCATCCCGTGCATATAAATGGTTGGAACTGCTCAATCCTACAGAATTCATGGCTAATACCATGGTCGAGCGGGAGTTTGTGGAATGCACCTCATCTGTTATACAAGCACTTGATCTATTTAGAAAATTGTATCCAGATCACAGGAAGAAAGAGATCAACAGGTCCATCGAAAAAGCTGTGCAATTTATACAAGACAATCAAACACCAGACGGTTCATGGTACGGAAATTGGGGTGTTTGCTTCATTTACGCTACTTGGTTTGCTCTTGGAGGCCTAGCAGCAGCTGGTGAAACTTACAACGATTGTTTAGCTATGCGCAATGGTGTCCACTTTTTGCTCACGACACAAAGAGATGATGGAGGTTGGGGTGAAAGCTATTTATCATGCTCCGAACAGAGATATATACCATCAGAAGGAGAAAGATCAAACCTTGTGCAAACATCATGGGCTATGATGGCTCTAATTCATACGGGACAGGCTGAGAGAGATTTGATTCCTCTTCATCGTGCTGCCAAACTTATCATCAATTCACAACTTGAAAACGGCGATTTTCCTCAACAGGAAATAGTAGGAGCGTTCATGAATACATGCATGCTACACTATGCTACATACAGAAACACCTTCCCATTATGGGCACTCGCAGAATACCGA

>AT1G66960

ATGTGGAGGTTAAAGGTAGGAGAAGGACCTTATTTATTCAGCAGCAACAACTTCGTGGGACGTCAAACATGGGAGTTTGACCCCAAAGCCGGCACACGGGAGGAACGAACCGCAGTCGAAGAAGCTCGCCGGAGTTTCTTCGACAACCGTTCTCGTGTTAAATCCAGTGATCTATTGTGGAAAATGCAATTTCTAAAAGAGGCAAAATTTGAGCAAGTGATTCCGGTAAAAATTGACGGTACTTATGAAAAAGCGACAAATGCATTACGGCGAGGAGTTGCTTTCTTATCAGCTTTGCAAGCCTCCGACGGCCACTGGCCGGGAGAGTTCACCGGACCGCTCTGCATGCTTCCGCCATTGGTATTTTGTTTGTACATTACTGGACACTTGGAAGAGGTATTCGATGCAGAGCATCGCAAAGAGATGCTTCGATATATCTATTGTAACGAAGATGGTGGATGGGGATTCCACATTGAGAGCAAAAGCATTATGTTCACTACCACGCTGAATTACATATGCTTGCGTATACTTGGAGTAGGTCCCGCATGCAAACGGGCCAGGCAATGGATTCTTAGCCATGGCGGTGTGATTTATATTCCTTGTTGGGGAAAAGTTTGGCTCTCGGTACTTGGAATCTATGATTGGTCTGGAGTCAACCCGATGCCTCCCGAGATTTGGTTGCTACCTTATTTCCTACCAATTCACAGCTATACCCGGATAACATATATGCCCATCTCTTATCTATATGGCAAAAAATTCGTGGGTCAAATTACACCTCTTATTATGCAACTACGTGAAGAACTACACTTACAACCTTATGAAGAAATCAACTGGAACAAAGCGCGACATCTATGCGCAAAGGAAGACAAGTACTATCCCCATCCTCTAGTTCAAGATTTGATATGGGATGCTCTCCACACCTTCGTGGAGCCTTTGCTTGCAAGTTGGCCGATAAACAAACTTGTAAGGAAAAAGGCTCTTCAGGTGGCAATGAAACACATACATTACGAGGACGAAAACAGTCACTATATCACCATTGGATGTATTGAAAAGAATTTGTGCATGCTTGCTTGCTGGATTGACAACCCGGACGGGAATCACTTTAAAAAGCATCTCTCTAGAATTCCGGACATGATGTGGGTAGCTGAAGATGGAATGAAAATGCAGTGCTTTGGAAGTCAACTTTGGATGACGGGATTTGCAGTTCAGGCTTTACTAGCAGTGCTCAGGAGAGCACACGATTACATAAAGAAATCACAGGTTAGAGACAACCCATCAGGTGACTTCAAGAGCATGTACCGCCACATCTCCAAAGGAGGATGGACTCTTTCTGATCGAGATCATGGATGGCAAGTTTCAGATTGTACAGCTGAAGCTGCTAAGTGTTGCATGTTGCTTTCCACAATGCCAACTGATATCACTGGAGAGAAAATCAATCTTGAACAACTATACGATTCTGTTAATCTCATGTTATCTCTACAAAGTGAAAATGGAGGTTTTACTGCATGGGAACCTGTTCGCGCCTATAAATGGATGGAATTGATGAATCCCACAGATTTGTTTGCTAATGCTATGACCGAGCGTGAATATACAGAATGTACCTCAGCTGTGTTACAAGCTTTGGTTATATTCAATCAACTATATCCGGATCATAGGACAAAAGAGATCACTAAGTCGATTGAGAAAGCAGTGCAATTCATAGAAAGCAAACAATTGCGAGATGGTTCATGGTACGGAAGCTGGGGTATTTGTTTCACTTATGGGACATGGTTTGCTCTTTGCGGCCTAGCAGCGATTGGTAAGACATACAACAATTGTCTATCTATGCGCGACGGTGTACATTTCCTTCTTAATATACAAAATGAAGATGGGGGTTGGGGTGAAAGCTATATGTCATGCCCTGAACAGAGATACATACCATTAGAGGGGAATAGATCAAACGTAGTGCAAACCGCGTGGGCTATGATGGCTCTGATTCACGCTGGACAGGCTAAGAGAGATCTTATACCTCTACATAGTGCTGCAAAATTTATTATCACGTCGCAACTGGAAAACGGAGATTTTCCTCAACAGGAACTATTAGGAGCGTCTATGAGTACATGCATGCTACACTATTCTACATACAAAGACATCTTCCCACCATGGGCACTTGCAGAGTACCGG

>AL2G26090

ATGTGGAGGTTAAAGATAGGAGAAGGACCTTACTTGTTCAGCAGCAACAACTTTGTCGGACGTCAAACATGGGAGTTTGATCCCAAAGCTGGCACAATCGAGGAACGAGCCACTGTAGAAGTAGCTCGCCGGAGTTTCTTGGTCAACCATTCTCGTGTTAAATGCAGTGATCTCTTGTGGAGAATGCAATTTTTGAAAGAGGCTAAATTCGAGCAAGTGATCCCGGTGAAGATTGATGACACGCATGAAAATGCAACGGATGCATTACGGAGAGGAGTTTCTTTCTTCTCGGCTTTGCAGGCCTCCGATGGCCACTGGCCGGGAGAAATCACAGGACCTCTCTTCTTCCTTCCTCCATTGGTATTTTGTTTGTATATCACAGGACACCTTGAGAAGATATTCGATGAAGAACATCGCAAAGAGATGCTTCGACATGTCTATTGTAACGAAGATGGTGGATGGGGATTACACATTGAGAGCAAGAGCATTATGTTCTGCACCGTGTTGAATTACATTTGCTTGCGTATGCTCGGAGAAGGTCCTGCGTGTAAACGGGCTAGACAATGGATTCTTGATCGTGGTGGTGTGACCTATATTCCGTCCTGGGGGAAAATTTGGCTATCGATACTAGGAATCTATGATTGGTCCGGAACCAATCCAATGCCTCCTGAGATCTGGTTGCTTCCTTCTTTCCTTCCAATACACTGTTATTGCCGGATGGTTTACATGCCCATGTCGTATCTATATGGTAAGAGGTTTGTTGGTCCAATAACACCTCTTATTCTGCAACTGCGTGAAGAACTCCATTTAGAACCTTACGAAGCAATCAACTGGAATAAAACACGGCGTCTTTACGCAAAGGAAGACATGTATTTTCCCCATCCTTTGGTTCAAGATTTGATATGGGACGCTCTTTACCTCTTTGTGGAGCCGTTCCTTACCCGTTGGCCGTTAAACAAGCTTGTAAGGGAAAAGGCTCTTCGGCTGGCAATGAAACACATACATTACGAAGACGAGAATAGCCATTACATCACTATCGGATGTGTTGAGAAGGTGTTGTGCATGCTTGCTTGTTGGATTGAAAACCCGAATGGGGACTACTTCAAAAAGCATCTTGCTAGAATACCGGATTACATGTGGGTCGCGGAAGACGGAATGAAAATGCAGAGCTTTGGAAGTCAACTATGGGATACAGGGTTTGCGGTTCAAGCTATACTTGCTGTACTTAAGAGAGGACATGATTACATTAAGAAATCTCAGGTTAGAGAGAATCCTTCTGGTGACTTTAAGAGCATGTACCGCCACATATCGAAAGGAGCATGGACTTTATCTGATCGAGATCATGGATGGCAAGTTTCAGACTGTACAGCAGAAGCTTTGAAGTGTTGCCTGCTGCTTTCTATGATGCCAGCTGAGGTGGTTGGCCACAAAATAGATCCCGAACAAATATACGATTCGGTTAATCTCTTGCTATCTTTTCAGAGTGATAATGGAGGTGTGACTGCATGGGAGCCTGTCCGTGCATATAAATGGATTGAATTACTCAATCCCACAGAATTTCTGGCTAATCTTGTGGTTGAGCGTGAATATGTGGAATGTACATCAGCTGTTATACAAGCTTTGGTTCTGTTCAAGAAACTCTATCCGGATCACAAGACAAAAGAGATCACCAGGTCCATCGAGAAAGCGGTGCATTTCTTGGAAAACGAACAAAGGTCGGATGGTTCATGGTATGGAAATTGGGGTGTTTGTTTCATTTATGGCACATGGTTTGCTCTTGGCGGCCTGGCAGCCGCGGGTGAAACATATAAAACTAGCCAAGCAATGCGTAAAGGTGTTGAATTCTTACTCAGGACACAGAAAGATGATGGAGGTTGGGGTGAAAGCTATCTGTCATGCCCTGAACAGAGATATATACCATTAGAAGGGAAAAGATCCAACCTGGTGCAAACAGCTTGGGCACTAATGGGGTTGATTCATGCTGGACAGGCCGAGAGAGATCCAATAACTCTACACCGTGCTGCGAAACTTATCATCAATTCGCAAATGGAAAATGGAGATTTCCCTCAGCAGGAAATAGTAGGAGTGTTCATGAGGAATTGCTTGATACACTATGCTACCTATAGAAACACTTTCCCATTATGGGCACTTGCTGAATACCGA

>AT1G78960

ATGTGGAAGTTGAAGATAGGAGAGGGACCTTACTTGTTCAGCAGCAACAACTTCGTCGGACGTCAAACATGGGAGTTTGATCCCAAAGCCGGCACACCTGAGGAACGAGCCGCCGTCGAAGATGCTCGCCGGAACTATTTAGACAACCGTCCCCGTGTTAAATGCAGTGATCTCTTGTGGCGAATGCAATTTTTGAAAGAGGCGAAATTCGAGCAAGTGATCCCGGTGAAGATCGACGACACTTACAAAAACGCGACGGATGCGTTACGGAGAGCAGTTTCTTTCTACTCGGCTTTGCAGTCCTCCGATGGCCACTGGCCGGCGGAAATCACCGGAACTCTCTTCTTCCTTCCTCCATTGGTATTTTGTTTCTATATCACAGGACACCTCGAGAAGATATTTGATGCAGAACATCGCAAAGAGATGCTTCGGCATATCTATTGCAACGAAGACGGTGGATGGGGTTTACATATTGAGGGAAAGAGCGTTATGTTCTGCACCGTACTGAATTACATATGCTTGCGTATGCTCGGAGAAGGTCCCGCCTGCAAACGGGCCAGGCAATGGATTCTTGACCATGGTGGTGTGACTTATATTCCTTCTTGGGGAAAAATTTGGCTCTCGATACTCGGAATCTATGATTGGTCTGGAACCAACCCAATGCCTCCCGAGATTTGGTTGCTGCCTTCTTTCTTTCCAATACACTGTTATACCCGGATGGTTTATATGCCCATGTCTTATCTATATGGGAAACGATTTGTTGGTCCTCTTACACCTCTTATTATGCTATTGCGCAAAGAACTGCACTTGCAACCTTATGAGGAAATCAATTGGAACAAAGCGCGCCGTCTATGTGCAAAAGAAGACATGATTTATCCTCATCCTCTGGTTCAAGATTTGTTATGGGACACTCTTCACAATTTTGTGGAGCCTATCCTTACAAATTGGCCGTTAAAAAAACTTGTACGGGAAAAGGCTCTTCGAGTGGCAATGGAACACATACATTATGAGGACGAAAATAGCCATTATATTACCATCGGATGTGTTGAGAAGGTTCTGTGCATGCTTGCTTGCTGGATCGAGAATCCTAATGGAGATCACTTTAAGAAACATCTCGCTAGAATTCCGGACTTCATGTGGGTTGCTGAAGACGGACTGAAAATGCAGAGCTTTGGAAGTCAACTCTGGGACACAGTTTTTGCGATTCAAGCTTTACTTGCTGTACTCAGGAAAGGACACAGTTTCATCAAAAAATCCCAGGTTAGAGAAAACCCTTCAGGTGACTTTAAGAGCATGTATCGCCATATTTCCAAAGGAGCCTGGACTCTGTCCGATCGAGATCATGGATGGCAAGTCTCAGATTGTACAGCTGAAGCTTTGAAGTGTTGCATGCTGCTCTCCATGATGCCAGCTGAGGTCGTTGGCCAGAAAATAGATCCTGAACAATTATATGATTCTGTTAATCTCTTGCTATCGTTGCAGGGTGAAAAAGGAGGTTTGACTGCATGGGAGCCTGTCCGTGCACAAGAATGGCTGGAATTGCTCAATCCCACAGATTTCTTTACTTGTGTTATGGCTGAACGCGAGTATGTAGAATGTACCTCAGCTGTTATACAAGCTTTGGTACTGTTCAAACAACTTTATCCGGATCACAGGACAAAAGAGATCATCAAGTCGATTGAGAAAGGGGTGCAATTCATTGAAAGCAAACAAACGCCTGATGGTTCATGGCATGGAAACTGGGGTATCTGTTTCATCTACGCTACTTGGTTTGCTCTGAGCGGCCTAGCAGCCGCTGGTAAAACTTACAAAAGTTGCCTGGCTGTGCGCAAAGGTGTAGATTTCCTGCTTGCGATACAGGAAGAAGATGGAGGTTGGGGCGAAAGCCATCTGTCATGCCCTGAGCAGAGATACATACCATTAGAAGGGAACAGATCAAACCTAGTGCAAACCGCATGGGCTATGATGGGTTTGATTCATGCCGGACAGGCCGAGAGAGATCCTACACCTCTTCACCGTGCTGCGAAACTTATCATCACTTCACAACTTGAAAATGGGGACTTTCCGCAACAGGAAATATTAGGAGTGTTCATGAATACATGCATGCTACACTATGCTACGTACAGAAACATCTTCCCACTATGGGCACTCGCGGAATATCGG;

(((AT1G78500:0.02452,AL2G38900:0.03375):0.07679,(AL2G38860:0.08752,AL2G38880:0.0464):0.05683):0.02193,(AT5G42600:0.13692,(((AT3G45130:0.04108,AL5G23630:0.03065):0.28216,(AT2G07050:0.01814,AL3G44070:0.0098):0.21003):0.17951,(((AL2G39420:0.02721,AT1G78970:0.02371):0.09407,(AL2G26090:0.08932,(AT1G66960:0.12742,AT1G78960:0.08019):0.02914):0.02309):0.05448,(AT1G78950:0.13959,(AL2G39400:0.02131,AT1G78955:0.02326):0.11735):0.0419):0.08621):0.2175):0.04636,(AT5G36150:0.14029,(AT4G15340:0.09168,((AL8G20190{Foreground}:0.02676,AT5G48010{Foreground}:0.03318):0.06683,AT4G15370:0.0708):0.01311):0.06517):0.02551)

**B) CYP708A codon-based alignment and input for Newick tree**

>AL1G64190_v2_1

ATG---------TGGGGTGTTGCAGTTATAGCTTTAGTGGTCGTCAAGATCAGTCTCTGGCTCTATAGATGGGCTAACCCTAACTGCTCCGGCAAGCTACCTCCGGGTTCAATGGGGTTTCCGGTGATCGGAGAAACCATTGAGTTCTTCAAACCTTACAGTTTCGACGAAATCCATCCATTTGTCAAGAAAAGAATGTTCAAGCACGGTTCGTTGTTCCGGACGAGTATCCTTGGCTCTAAAACGATTGTTTCGACAGACCCGGAAGTGAACTTTGAGATTCTCAAACAAGACAACAGGGGTTTCATCATGAGCTATCCAGAAGCTCTTGTTAGAATCTTTGGGAAAGATAACTTGTTCTTCAAGCAAGGATTCCACAGGTACATGAGACAGATTGCTCTGCAGCTTCTTGGCCCTGAACGTTTGAAGCAGAGATTTATACAACAAATCGACATAGCTACACGCGAGCATCTCAAATCTGTCTCCTTCCAAGGCGTTTTAGACGTCAAAGACACTAGTGGAAGATTGATATTGGAACAAATGATCCAGATGATCATAAGTAACATCAAACCTGAGACTAAGATCAAACTGATAGAAAGCTTTAGAGATTTCAGCTTCGACTTGGTTATGTCCCCTTTTGATCCTTCTTTTTGGAACGCTCTTTACAATGGACTTGCACGCAGAAATGTGATGAAGATGATAAAAAGAATGTTCAAAGAGAGGAGAGAAGATGAATTAAAATATGGAGACTTCATGGAGACGATGATATATGAGGTGGAGAAAGAAGGCGATACGGTGAACGAGGAAAGATCTGTAGAGCTAATACTAAGTTTACTGATTGCGTCTTATGAAACTACTTCTACAATGACTGCACTAACTGTGAAATTCATAGCTGAAAACCCCAAAGTGCTTATGGAGCTGAAGAGAGAGCATGAGACCATCCTCCAAAATAGAGGTGATAAAGAGTCTGGAGTTACTTGGAAAGAATACAGATCCATGATGACTTTCACCCATATGGTTATCAATGAGTCTCTTCGTTTAGGAAGTTTGTCACCTGCTATGTTTAGAAAGGTGGTGAGG------------------TATACAATTCCAGCAGGATGGATTGTATTGGTTGTTCCATCTCTTCTTCATTATGACCCTCAAATCTATGAACAACCATGTGAGTTTAATCCATGGAGATGGGAGGGGAAGGAATTGCTTTGTGGCTCTAAAACGTTCATGGCGTTTGGTGGTGGTGCAAGACTATGTGCAGGAGCAGAGTTTGCTAGGCTTGGGATGGCTATATTTCTTCATCATCTAGTCACAACGTATGACTTGTCTTTGATCGATAAATCTTATATCATTCGAGCACCCTTACTTCGATTCTCCAAGCCGATACGCATAACGATCTCC

>AT1G55940_TAIR10

ATGAAAGAAATGTGGGGTGTTGCAGTTATAGCTTTAGTGGTCGTCAAGATCAGTCTCTGGCTCTATAGATGGGCTAACCCTAACTGCTCCGGCAAACTACCTCCAGGTTCAATGGGGTTTCCGGTGATCGGAGAAACCGTTGAGTTCTTCAAACCTTACAGTTTCAACGAAATCCATCCATTTGTCAAGAAAAGAATGTTCAAGCACGGTTCATTATTCCGGACGAATATCCTCGGCTCTAAAACGATTGTTTCGACAGATCCGGAAGTGAACTTTGAGATTCTCAAACAAGAGAACAGGTGTTTCATCATGAGCTATCCAGAAGCTCTTGTTAGAATCTTTGGGAAAGATAACTTGTTCTTCAAGCAAGGCTTCCACAGGTACATGAGACATATTGCTCTGCAGCTTCTTGGCCCTGAGTGTTTGAAGCAGAGATTTATACAACAAATCGACATAGCAACAAGCGAGCATCTCAAATCTGTCTCCTTCCAAGGCGTTGTAGACGTCAAAGATACTAGTGGAAGATTGATATTGGAACAAATGATCCTGATGATCATAAGTAACATCAAACCTGAGACTAAGAGCAAACTGATAGAGAGTTTTAGAGATTTCAGCTTTGATTTGGTTAGGTCCCCTTTTGATCCTTCTTTTTGGAACGCTCTTTACAATGGTCTTGCACGCAGCAATGTGATGAAGATGCTAAAAAGAATGTTCAAAGAGAGGAGAGAAGATGACTCAAAATATGGAGACTTCATGGAGACAATGATATATGAGGTGGAGAAAGAAGGCGATACGATCAACGAGGAAAGATCAGTAGAGCTAATACTTAGTTTACTGATTGCGTCTTATGAAACTACTTCTACTATGACTGCATTAACTGTGAAATTCATAGCTGAAAACCCCAAAGTGCTTATGGAGCTGAAGAGAGAACATGAGACCATCCTCCAAAACAGAGCTGATAAAGAATCTGGAGTTACTTGGAAAGAATATAGATCCATGATGAATTTCACCCATATGGTTATCAATGAGTCTCTTCGTTTAGGAAGTTTGTCACCTGCTATGTTTAGAAAGGCGGTGAATGATGTGGAGATCAAAGGGTATACAATTCCAGCAGGATGGATTGTATTGGTGGTTCCATCTCTTCTTCATTATGACCCTCAAATCTATGAACAACCATGTGAGTTTAATCCATGGAGATGGGAGGGGAAAGAATTGCTTTCTGGCTCTAAAACGTTCATGGCGTTTGGTGGTGGTGCAAGACTATGTGCAGGAGCAGAGTTTGCTAGGCTTCAGATGGCTATATTTCTTCATCATCTAGTCACAACGTATGACTTCTCTTTGATCGATAAATCTTATATCATCCGAGCACCCTTACTTCGATTCTCCAAGCCGATACGCATAACGATCTCC

>AT3G44970_TAIR10

ATGAACTTGTTTTGGAACACAGGAGTTATAGTTTTAGTGGTTGCGAGGGTTGGTCATTGGTGGTACCAATGGTCAAACCCTAAGTCTAACGGCAAGTTACCTCCGGGATCAATGGGTTTCCCGATCATCGGAGAGACATTAGATTTCTTTAAGCCTTATGGATTCTACGAGATCTCCCCATATCTCAAGAAAAAGATGTTAAGGTATGGGCCATTATTTCGGACAAACATTCTTGGAGTAAAAACCGTGGTATCGACGGATAAGGATGTTAACATGGAGATTTTAAGACAAGAGAACAAGTCTTTCATTTTAAGTTATCCGGATGGTTTAATGAAGCCATTGGGAAAAGATAGCTTGTTCTTGAAGATTGGAATCCACAAGCACATCAAACAAATAACTCTTCATCTTTTGTCCTCCGAGGGTTTAAAACGGAAAATATTAAAAGATATGGACCGTGTGACACGCGAGCATCTTAGTTCGAAAGCTAAAACAGGAAGATTGGATGTTAAGGACGCAGTTTCAAAATTGATAATAGCCCACTTGACACCTAAGATGATGAGTAATCTCAAACCACAGACTCAAGCAAAGCTTATGGGAATTTTTAAGGCCTTCACTTTTGATTGGTTTCGGACATCCTATCTTATCTCTGCCGGGAAGGGTCTCTACAACACCCTTGCATGCCGAGAGGGGATGCGAGAGATAAAGGATATATATACAATGAGGAAAACGTCTGAGGAGAAGTACGATGACTTCCTCAACACGGCAATAGAAGAGTCAGAGAAAGCAGGAGAGTTATTGAACGAAAATGCAATTATAACCCTCATCTTTACCCTTTCATGTGTCACTCAAGATACCACCTCAAAGGCGATTTGCTTGGCTGTGAAATTCCTATTGGAAAACCCAAAAGTTCTCGCAGAGTTAAAGAAAGAACATGAGGTGATCCTTGAAAGCAGAGAAGACAAAGAAGGTGGAGTTACTTGGGAAGAATACAGACACAAGATGACTTTCACCAACATGGTTATCAATGAGTCTCTTCGAATAACAAACTTGGCTCCTATGTTATTTAGAAAAGCGGTAAAGGATGTGGAAATCAAAGGATACACCATTCCAGCAGGTTGGATAGTGATGATTATACCTTCAGTGGTTCATTTTGATCCTGAAATCTATGAAAACCCTTTTGAGTTTAATCCATGGAGATGGGAGGGGAAAGAGTTGCGGGCTGGATCTAAAACGTTCATGGTGTTTGGAACCGGACTTAGACAATGCGCTGGTGCAGAGTTTGCTAGGCTTCAGATTTCAGTATTTCTTCATCATCTTGTTACCACTTATAATTTCTCGTTGCACCAAGACTGCGAGGTGCTTCGTGTACCAGCTGCTCACCTCCCTAATGGCATATCTATAAACATCTCC

>AL8G20160_v2_1

ATGAGCTTCGTCTGGTCCGCTGCGGTCATAACTGTAGCAGTTGTTGTGATTAGCAAATGGTTATACCGGTGGTCGAACCCGAAGTGCAATGGCAAGTTACCTCCGGGATCAATGGGTTTACCGATCATCGGAGAGACATGCAACTTCTTTGAGCCCCATGGATTATACGAGATCTCACCCTTTGTCAAGAAGAGGATGTTAAAGTACGGGCCATTGTTTCGGACCAACATTTTCGGATCCAACACCGTGGTTTTGACAGAACCTGATATCATCTTTGAGGTTTTCCGACAAGAGAACAAGTCTTTTGTGTTTAGCTATCCAGAAGCCTTTGTAAAGCCATTTGGAAAAGAAAACGTGTTCCTCAAACATGGGATCCACAAGCACGTCAAACAAGTCAGTCTTCAACATATTGGCTCTGAGGCCTTAAAAAAAAAGCTGATTGGAGAAATAGACAGAGTGACCTATGAGCATCTTAAATCAAAGGCTAGCCAGGGTAGCTTCAATGCTAAGGAGACAGTTGAAAGCTTGATAATGGCACACCTGACCCCGAAGATAATAAGTAACCTCAAACCAGAAACACAAACAAATCTTGTGGACAATATAATGTCCCTAGGATCTGAATGGTTTCAGTCACCGTTAAAGCTTACGACATGGATTTCTATCTTCAAAGTCTTTGCCCGAAGAGACGCACTGCAGGTGATAAAGGACGTTTTTATGAGGAGGAAAGCGTCCAGAGAAATGTGCGGAGACTTCCTCGACACAATGGTAGAAGAGGGGGAGAAAGAAGAGGTCATTTTTAATGAAGAAGGTGCTATAAATCTCATCTTCGCTATTCTGGTAGTCGCTAAAGAATCAACCTCTTCCGTTACTAGCTTGGCCATCAAATTTCTTGCCGAAAACCATAAAGCTCTCGCAGAGTTGAAGAGAGAGCATGAGGCCATCCTTCAAAATAGAAATGATAAAGAAGCTGGAGTTGGCTGGGAAGAATACAGACACCACATGACTTTCACTAACATGGTGATCAATGAGACTCTTCGAATGGCAAACATGGCTCCTATAATGTATAGGAAGGCTGTGAACGATGTCGAAATCAAAGGATACACAATTCCAGCGGGCTGGATTGTGGCGGTTATACCTCCAGCCGTCCATTTCAATCATGAGATTTATGAGAATCCTTTGGAGTTCAATCCATGGAGATGGGAGGGGAAAGAGTTGCGGTCTGGATCTAAGACGTTCATGGTATTTGGAGGAGGAGTGAGGCAATGTGTCGGCACGGAGTTTGCGAGACTCCAAATTTCTATCTTCATTCATCATCTTGTAACCAAATATGATTTCTCACTGGCCCAAGAATTCGATTTCATCCGTGCACCACTACCTCACTTCCCCAAAGGACTGCCTATCAAGATTTCC

>AT5G48000_TAIR10

ATGAGCTTCGTGTGGTCCGCTGCGGTCATAGCTGTAGCCGCTGTTGTGATTAGCAAATGGTTATACCGATGGTCGAACCCGAAGTGCAATGGCAAGTTACCACCGGGATCAATGGGTTTACCGATCATCGGAGAGACATGCGACTTCTTTGAGCCCCATGGATTATACGAGATCTCACCCTTTGTCAAGAAGAGGATGTTAAAGTACGGGCCATTGTTTCGGACAAATATTTTCGGATCGAACACCGTGGTTTTGACAGAACCTGATATCATCTTCGAAGTTTTTCGGCAAGAGAACAAGTCTTTTGTGTTTAGCTATCCAGAAGCTTTTGTCAAGCCATTTGGAAAAGAAAACGTGTTCCTCAAACATGGAATCCACAAGCACGTCAAGCAAATCAGTCTTCAACATCTTGGCTCTGAGGCTTTAAAAAAAAAGATGATAGGAGAAATAGACAGAGTAACCTATGAGCATCTTAGATCGAAGGCTAACCAGGGTAGCTTCGATGCTAAGGAGGCAGTTGAAAGTGTTATAATGGCGCACTTGACCCCAAAGATAATAAGTAACCTCAAACCAGAAACACAAGCAACTCTTGTGGACAATATAATGGCCCTAGGATCTGAATGGTTTCAGTCACCCTTGAAGCTTACGACTTTGATTTCTATCTACAAAGTCTTTGCACGTAGATACGCCCTCCAGGTGATAAAGGACGTTTTCACGAGGAGGAAAGCGTCCAGAGAAATGTGCGGAGACTTCCTCGACACAATGGTAGAAGAGGGGGAGAAAGAAGACGTCATTTTTAATGAAGAAAGTGCTATAAATCTCATATTCGCTATTTTGGTCGTCGCTAAAGAATCTACCTCTTCCGTTACTAGCTTGGCCATCAAATTTCTTGCCGAAAACCATAAAGCTCTCGCAGAGTTGAAGAGGGAGCATGCGGCCATCCTTCAAAATAGAAATGGTAAAGGAGCTGGAGTTAGCTGGGAAGAATACAGACACCAAATGACTTTCACTAACATGGTGATAAATGAGACTCTTCGAATGGCAAACATGGCTCCTATAATGTATAGAAAGGCTGTGAACGATGTCGAAATCAAAGGTTACACAATTCCAGCGGGCTGGATTGTGGCGGTTATACCACCAGCTGTCCATTTCAATGATGCTATTTATGAGAATCCTTTGGAGTTCAATCCATGGAGATGGGAGGGGAAAGAGTTGCGGTCTGGATCTAAGACGTTCATGGTGTTTGGAGGTGGAGTGAGACAGTGTGTCGGCGCGGAGTTTGCGAGACTACAAATTTCTATCTTCATTCATCATCTTGTAACAACCTACGATTTCTCATTGGCCCAAGAATCGGAGTTCATCCGTGCACCACTCCCATACTTCCCCAAAGGACTGCCTATCAAGATTTCC

>AT1G78490_TAIR10

ATGAGCTCCATATGGAACGTTGCAATGGTAGCTTTGGTGGTTGTGAGGATCAGCCACTGGCTTTACCGATGGTCAAACCCTAAGTGCCCTGGCAAGTTACCACCTGGATCGATGGGTTTTCCAATCATTGGAGAGACTCTCGATTTCTTTAAGCCTTGTGGAGTCGAAGGTATCCCAACCTTTGTTAAGAAGAGGATGATAAGGTATGGGCCGTTGTTTCGAACAAACATTTTTGGTTCCAAAACCGTGGTTTCGACAGATCCGGATGTGATCCACCAGATTTTCCGGCAAGAGAACACGTCTTTTGAGCTAGGCTATCCAGACATATTTGTGAAAGTATTTGGAAAAGATAATTTGTTCTTGAAGGAAGTGATCCACAAGTACCTCCAAAAAATCACTATGCAAATTCTTGGCTCCGAGGGTTTGAAGCAAACAATGTTAGGAAACATGGACAAAGCAACCCGCGACCATATTAGGTCCATTGCTAGCCAGGGGAGCTTCAATGTTCGTAAAGAAGTTGAAAACTTGGTAGTAGCGTACATGACACCAAAGCTGATAAGTAACCTTAAACCAGAGACGCAATCAAAGCTTATAGATAATCTCAACGCCTTCAATCTTGATTGGTTTAAGTCCTTCTTAAGACTCTCTACTTGGAAAGCTGTCACCAAAGCCCTCTCACGCGAAGAAGCTATCCAGGTGATGAAAGACGTTCTCATGATGAGAAAAGAGACGCGAGAGAAGCAAGAAGACTTCCTTAACACGCTTCTAGAAGAACTGGAGAAAGACGGTAGCTTTTTCGACCAAGGATCGGCTATAAATCTCATCTTCCTTCTGGCGTTTGCCTTGAGAGAAGGTACCTCTAGTTGTACTGCTTTGGCCGTGAAGTTCATATCCAAAGACCCGAAAGTGCTTGCAGAGCTCAAGCGTGAGCATAAGGCAATTGTAGACAACAGAAAAGATAAGGAAGCTGGAGTTAGCTGGGAAGAATATAGACACAACATGACTTTCACCAACATGGTTAGCAATGAGGTGCTTCGCCTAGCAAACACGACCCCTTTGTTGTTTCGAAAGGCGGTGCAAGATGTTGAGATCAAAGGATATACAATTCCGGCTGGTTGGATTGTGGCGGTTGCACCTTCAGCGGTTCATTTTGATCCTGCAATCTATGAGAACCCATTTGAGTTTAATCCATGGAGATGGGAGGGGAAAGAAATGATTTGGGGATCTAAAACGTTCATGGCGTTTGGATATGGAGTTAGACTTTGTGTAGGTGCAGAGTTTTCACGGCTTCAAATGGCAATCTTCCTCCATCATCTTGTGGCATATTACGATTTCTCAATGGTCCAAGACTCCGAGATCATTCGTTCACCATTCCATCAATACACCAAGGATCTGCTTATAAACATCTCT

>AL2G38880_v2_1

ATGAGTTTCATGTGGACAGTAGCGGTCATAGCTTTAGTGGTTGTGAGCATCAGCCACTGGTTGTACCGATGGTCGAACCCTAAGTGCATCGGCAAGTTACCTCCAGGATCAATGGGTTTCCCGATCATCGGAGAAACAATCCATTTCTTTAAGCCTTATGGATTCTACGAGATTCCACCCTTTCTCAAGAAGAGATTATTAAGGTATGGGCCGTTGTTTCGTACGAACATTTTTTGTTCCAAAACTGTGTTTTCGACGGAACCAGATGTTATCTTCGAGATTTTCCGGCAAGAGAACAAGTCTTTCGCGCTTGGCTATCCAGACATGTTTGTCAAGGTACTTGGAAAAGATAATTTGTTCTTCAAGCCCGGGATACATAAGCACCTCAAACAAATTACTCAACATCTTCTTGGCGCTGAGAGTTTGAAGCAAAAGATGATAGGAAACATGGACCAAGAGATCCGCAACCATCTTAGATTGAAGGCTAGTGAGAGCAGATTTGACGTTAAAGACACAGTT---------------------------------------------------------------------------------------------------------------------------------------------------------TTAGCACGCAGAGAAGCAATCGATGTGATAAAGGATGTTCTAACGAGGAGGAAAGAGTCGAAAGAGAAGCATGGAGACTTTGTAGACACGATGCTGGAAGATTTGGAGAAAGAAAACACTATTTTTGACCAAGGATCGGCTATAAGCCTCATTTTCAGTATACTAGTCGTGGCTAAAGAAGGTGTTCCTAATATCACTTCCATAGCTGTGAAATTCTTATCTCAAAACCCAAAAGCCCTTGCAGAGCTGAAGAGAGAGCACAAGGCGATCCTACGGAACAGAAAAGATAAA---GGTGGAGTTAGTTGGGAGGAATACAGACACAGA---------------------------------------------------------------------------------------------------TACACAATTCCAGCGGGCTGGATAGTGGCGGTTGTGCCTGCAATGGTTCATTTTGATGAAGCAACATATGAGAACCCTTTGGAGTTTAATCCATGGAGATGGGAGGGAAAAGAAATGATTTGGGGATCTAAAACATTTATGGTGTTTGGAGGTGGAGTGAGATTGTGTGTAGGCGCAGAGTTTGCAAGACTTCACATCGCATTATTCCTGCATCATCTTGTCACCACTTACGATTTCTCGTTGGTCCAAGATTGCGAGTTGATACGAACACCATTCCTTCACTTTACCAAAGGCCTCCTTTTAAACATCTCA;

(AT3G44970_TAIR10:0.32337,(AL1G64190_v2_1:0.03163,AT1G55940_TAIR10:0.02355):0.41914,((AL8G20160_v2_1{Foreground}:0.04138,AT5G48000_TAIR10{Foreground}:0.02843):0.20732,(AT1G78490_TAIR10:0.2548,AL2G38880_v2_1:0.14946):0.08484):0.06079)

**C) CYP705A codon-based alignment and input for Newick tree**

>AL1G42060_v2_1

GTTAGCTTTGATCTTCTAAATTGTTTCATCTTTACTCTCATATTCCTCATCTCAACTCTTTTTTTTGTTTTCTTCTATAAAAAAACAAATGAT------GGATTTGATTTGCCTCCAAGCCCTCCTTCTCTTCCCATCATTGGTCATCTTCACCTTCTCCTCTTTGCTTCAATCCACAAGTGTTTTCAAAAAATCTCATCCAAATACGGACCTTTTCTCCATCTTCGCATTTTCCATGTCCCCATTATTCTTGTGTCCTCTGCCTCAGCGGCTTACGATATCTTCAAGGCACACGATATTAAAGTCTCTTATCGAGGTGTTGCAATCGATGAATGCATTGTGTTTGGTTCTTCTGGATATTTCAGAGCTCCATCTGAAGATTACTGGAAATTCATGAGGAAGCTCATCATGGCTAGGGCGCTTGGACCCCAAGCGCTAGAGCGGACACGTGGTGTTCGTGTAGCTGAGTTAGAAAGGTTTCATAGAAATATACTCGATAAGGCGATTAAAAAACAAAGCCTTAAGATCGGTGAAGAAGCAAGGATACTCGTTAACAACACCCTCGGAAAAATGAGCCTTGGAAGTAGTTTTTCAGTAGAGAACAATGATGGAACAAAAGTCTCAGAATTTTCTATCAAGTTAGCTGATTTGTCTCAAATGTTTTGTGTGGCACAAATATTTCATAAGCCGCTAGAGAAGGTGGGGATCTCTCTTTTAAAGAAGCAGATTATGGATGTTTCACATAAATTTGAGGAGTTGCTAGAAAATATTCTTGTGAAGTACGAGGGAAAACAAAGTACTGAATTCATGGATGCATTATTGGCAGCTTATCGAGACGAAAACGCAGAGTATAAGATCACTAGAAACCATATCAAGGCATTATTAGCGGAGCTATTCTTTGGAGCCGGTGAGTCCTCTTCTTCAACAACACGATGGGCAATGGGAGAAATCTTCAACAACCCTAAGATCTTTGAGAGATTGAGAGAAGAAATAGATTTAGTGGTGGGAAATACAAGGTTGATTCAAGAAAGTGATCTACCAAAACTTCCATACTTGCAAGCAGTCGTCAAAGAATCTCTAAGATTGCACCCGGTGGGAGCGGTCTTGCCAAGAGAATTTACACAAGATTGTAACATTGGAGGGTTCTATATACATGAGGGAACATCACTTGTCATCAATGCTTATTCTATAATGAGAGATCCTGATATTTGGGAAGATCCTAATGAGTTTAAACCAGAGAGGTTTCTAACTACTTCAAGATCAGGACAAGAGGAAGAGAAAAAAGAGCAAACACTAAAGTTCCTCCCTTTTGGCGCGGGAAGGAGAGGATGTCCTGGATTAAATCTTGGTTATACCTTAGTAGAAACCACAATTGGAGTGATGGTGCAATGCTTTGACTGGGAGATCGAAGGAGATAAAGTCAACATGGAAGAAGGTTCAGGATTGGATTTGGCTCATCCCCTTAAGTGCACTCCTATT

>AT1G28430_TAIR10

GTCAGCTTTGATCTTCTAAATTGTTTCATCTTTACCCTCATATTCCTCATCTCAACTCTCTTTTTTGTTTTATTCTATAAGAAACCAAACGAT------GGATTTGATGTACCTCCAAGCCCTCCTTCTCTTCCCATCATTGGTCATCTTCATCTTCTCCTCTTTGCTTCAATCCACAAGTGTTTTCAAAAAATCTCATCCAAATACGGACCTTTTCTCCATCTTCGCATTTTCCATGTCCCCATTGTTCTTGTATCCTCTGCCTCAACGGCTTACGATATCTTCAAGACAAACGACATTAATGTCTCTTATCGTGGTGTTGCAATCGATGAATGCATTGTGTTTGGTTCTTTTGGATATTTCCGAGCTCCATGTGAAGATTACTGGAAATTCATGAGAAAGCTCATCATGGCCAGGGCACTCGGACCCCAAGCGCTAGAGCGGACACGTGGTGTTCGTGCAGCTGAGCTAGAGAGGTTTCATAGAAAATTACTCGATAAGGCTATGAAAAAACAAAGCCTTAAGATCGGTGAAGAAGCAAGGATACTCGTTAACAACACCCTCGGAAAAATGAGCCTAGGAAGTAGTTTTTCAATAGAGAACAATGATGGGACAAAAGTTTCAGAATATTCTATCAAGTTAGCTGATTTGTCTCAAATGTTTTGTGTGGCACAAATATTTCATAAGCCGCTTGAGAAGCTAGGGATCTCTCTTTTAAAGAAGCAGATTATGGATGTTTCACACAAATTTGAGGAGTTGCTAGAAAATATTGTTGTGAAGTACGAGGAAAAACAAAGTACTGAATTCATGGATGCATTATTGGCAGCTTATCAAGACGAAAACGCAGAGTATAAGATCACTAGAAACCATATCAAGGCACTACTAGCGGAGCTTTTCTTTGGAGCCGGTGAATCATCTTCTTCAACAACAAGGTGGGCAATGGGAGAAATTTTCAACAATCCTAGGATCTTTGAGAAACTAAGAACAGAAATCGATTCAGTGGTGGGGACAACAAGGTTGATTCAAGAAAGTGATCTACCAAAACTTCCTTACTTGCAAGCAGTCGTGAAAGAATCTCTAAGATTACACCCGGTGGGAGCGGTCTTGCCAAGAGAATTTACGCAAGATTGTAACATTGGAGGGTTCTATATACATGAGGGAACATCACTTGTCGTTAATGCTTATGCTGTGATGAGAGATCCTGATATTTGGGAAGATCCTAATGAGTTTAAGCCGGAAAGGTTTCTAGATGCCTCAAGATTAGGGCAAGAGGAGGAGAAAAAGGAGAAAACACTAAAGTTCCTCCCATTTGGCGCTGGAAGGAGAGGATGTCCTGGATTATATCTTGGTTATACCTTAGTAGAAACCACAATTGGAGTGATGGTGCAATGCTTTGACTGGGAAATCGAAGGAGATAAAGTCAACATGCAAGAAGGTTCCGGATTGGATTTGGCTCATCCCCTTAAGTGCACTCCTATT

>AT1G50520_TAIR10

ATCACCGTTGACTTTCAGCTCTGCTTCATCTTCATCCTCCTAGGAATCTTCTCACTCTTCTGTTCTGCTTTCTTCTTCAAGAAACCAAAAGACCCACAAGGCTGTGGTCTACCTCCGAGCCCACCCTCTCTACCGGTCATAGGTCATCTTCACCTTCTCCTCTCTGTTCCATGTCTCAAATCCTTTCAGAAACTCTCCTCCAAGTATGGTCCCCTCCTTCACCTTCGTGCATTCAATATCCCAATAGTTATAGTCTCTTCCGGCTCCATGGCCAACGAAGTTTTGAGGACCCAAGACCTGAACTTTGCTACCCGTCAATCTATAATGGAAAAATCATTACTTTTTGGATCTTTTGGCTTTGTCTCAGCTCCTTATGGAGATTACTGGAGATTCATGAAGAAGCTCTTAGTCACAAACCTTTTCGGGTCTCATTCCCTCGAGCAGACACGGCTCATTCGTGAGAAAGAACTCAAGACTTTCCGTACTATGTTGTTCGATAAGGCGGCAAAGAAGGGGACAGTTGATGTTGGTAAAGAGATGATGAAGCTAACGAATAACAGCATTTGCAGGATGATAATGGGGAGGAGGTGTTCAGAGGAGAACAGTGAAGCAGAGAAAGTCGAAGACTTGGTGATCAAATCATTTAGTTTGGTGAAGAAGGTCCTTATAGCTAACACGGTTGGTCGACTTCTCAAGAAGTTTGGGATCTCTCTGTTTGAGAAGGAAATCATGGAGGTCTCGCAGAGGTACGATGAATTGCTGGAAAAAATTATTAAAGAACACGAAGAGGATGAGGATAGAGACATGATGGATGTTCTGTTGGAAGTTTGTGCAGACGACAAGGCTGAGGTTAAGATTACCAGGAACCAAATCAAAGCGCTTATTGTGGAGCTTTTCCTTGGAGGCACTGATACTTCAGCACAAACAATACAGTGGATAATGGCCGAACTCATTAACCATCCCGAGATTCTTAAAATATTAAGAGAAGAGATAGAATCTGTTGTCGGAACAACGAGGTTTATTCAAGAAACAGATCTCTCAAACCTGCCGTATTTGCAGGCTGTGATGAAAGAAGGACAAAGACTACACCCACATTCGCCAATGTTGGTGAGGAATGCAACCAAAGGATGCAAGATTGGAGGGTACTACATACCGCAGAACACAACGATGCTAATAAACACCTATGCGATGATGATAGATCCAGATTCATGGGAAAATCCAGACAAGTTTCAGCCCGAGAGGTTCATGGTGTCTCCTTCAAAAGGGAAAGACGATGAGAGAGAACAGCTAGCTTTGAACTTCATTCCTTTCGGGAGCGGAAGAAGAGCATGTCCAGGAGAAAAATTGGGCTATCTCTTCACTGGAGTAGCCATTGGAACAATGGTACAGTGTTTTGACTGGATAATCGATGGAGATAAGGTTAACGTGGAAGAG---GCTGGACTGACCATGGCTCATCCACTTAAATGCACTCCTGTG

>AT1G50560_TAIR10

ATCAGCGTTGACTTTCAGCTCTGCTTCATCTTCATCCTCCTAGCAATCTTCTCACTCTTCTGTTCTGCTTTCTTCTTCAAGAAACCAAAAGACCCACAAGGCTGCGGTCTACCTCCGAGCCCACCGTCTCTACCGATCATTGGTCATCTTCACTTTCTCCTCTCTGTTCCATGTTACAAATCCTTTCAGAAACTCTCCTCCAAGTATGGTCCCTTCCTTCACCTCCGCGCTTTCAATATCCCTATAGTTCTAGTCTCGTCGGGCTCCATGGCCAACGAAGTTTTGAGGATCCAAGACCTGAACTTTGCTAGCCGTGATCCTATAATGGAAAAATCATTACTTTTTGGATCTTTTGGCTTTGTCTCAGTTCCTTATGGAGATTACTGGAGATTCATGAAGAAGCTCTTAGTCAAAAAACTTCTCGGGTCTCATTCCCTCGAGCAGACACGGCTCCTCCGTGGGAAAGAACTCCAAACTTTCCGTGCTATGTTGTTTGATAAGGCGGCAAAGAATGAGACTGTTGACGTTGGTAAAGAGATGATGAAGCTAACGAACAACAGCATTTGCAGGATGACAATGGGGAGGAGCTGTTCAGAGGAGAACGGTGAAGCAGAGCAAGTCAGGGGTTTGGTGACCAAATCACTTAGTTTGACGAAGAAGTTCCTCATAGCTAGCATTGTTGGTCAATTTTCCAAGCTGGTCGGGATCTCTTTGTTTGGGAAGGAAATCATGGAGGTCTCGCAGAGGTACGATGAATTGCTGGAGAAAATTATTAAGGAACACGAAGAAAATGAGGATAGAGACATGATGGATGTTCTATTGGAAGTTTGTGCAGATGACAACGCCGAGTTTAAGATTTCCAGGAACCAAATCAAAGCACTTTTTGTGGAGATTTTCCTTGCAGGCACTGATACTTCAGCACAAACAATACAGTGGATATTGGCAGAACTCATTAACCATCCCGAGATTCTTGAAAAGTTGAGAAAAGAGATAGAATCTGTTGTTGGGGTTAGGAGACTGATTCAAGAAACAGATCTCCCCAACCTGCCGTATTTACAGGCTGTGATGAAAGAAGGGCTAAGACTACACCCGCATACGCCAATCTTGGTGAGGAATGCAACAGAAGGATGCAAGATCGGAGGGTATTACATAGGGCAGAACACAACAATGATGGTAAACGCTTATGCGGTGCTGAGAGATCCGGATTCATGGGAATATCCAGAAGAATTTCAGCCCGAGAGGTTCATGACTTCTCCTTTAAAAGGGAAAGAAGATGAGAAAGCACAGCTAGCCTTGAACTTCATTCCTTTCGGGAGTGGAAGAAGAGGATGTCTCGGAAAAAACTTGGGTTATATCTTCATGGGAGTAGCCATTGGAACAATGGTGCAGGGTTTTGATTGGAGAATCAATGGAGATAAGGTTAACATGGAAGAG---ACTGGACTGACCATGGCTCATCCACTTAAATGCATTCCTGTT

>AL1G58200_v2_1

ATCACCGTTGACTTTCAGATCTGCTTCATTTTCATCCTCCTATGGCCCTTCTCACTCTTCTGTTATGGTTTCTTCTTCAAGAAACCAAAAGAGCCACGAGGCTGTGGTCTACCTCCGAGCCCACCGTCTCTACCAGTCATAGGTCATCTTCACCTTCTCCTTTCTGTTCCATGTCACAAATCCTTTCAGAAACTCTCCTCCAAGTATGGTCCCCTCCTTCACCTTCGCGCTTTCAATATCCCAATAGTTCTAGTCTCTTCCGGCTCCATGGCCTACGAAATCTTCAGGACTCATGACCTGAACTTTGCTACCCGTGACCCTATAATGGAGAAGTCGATACTTTTTGGTTCATTTGGCTTTATCTCAGCTCCTTATGGAGATTACTGGAGATTCGTGAAGAAGCTCTTAGTCACAAAGCTTCTTGGGACTCGTTCACTCGAACGGACACGGGTCATCCGTGGGAAAGAACTCATGAGTTTCCGTGCTATGTTGTTCGATAAGGCGGCAAAGAATGAGACTGTTGATGTTGGCAAGGAGATGATGAAGCTAACGAATAACAGCATCTGCAGGATGATCATGGGGAGGAGGTGTTCAGAGGAGACTGGTGAAGCAGAGCAAGTCAGGGGTTTGGTGACCAAATCACTTAGTTTGGTGAAGAAGTTCCTTATAGCTAGCACAGTTGGTCGAGCTTTCAAGAAGCTCGGGATCTCTCTGTTTGAGAAGGAAATCATGGAGGTCTCGCAGAGGTACGATGAATTGCTGGAGAAGATTATTAAAGAACACGAAGAGAATGAGGATAGAGACATGATGGATGTTCTGTTG------------GAAGACAATGCCGAGTTTAAGATTTCCAGGAACCAAATCAAAGCGCTTTTTGTGGAGCTTTTCCTTGGAGGCACTGATACTTCAGCACAAACAACACAGTGGATAATGGCAGAACTCATTAATCATCCCGATATTCTTAAAAGATTGAGAGAAGAGATAGAATATGTTGTCGGGGAAACGAGATTGATTCAAGAAACAGATCTCCCCAACCTGCCGTATTTACAAGCTGTGGTGAAAGAAGGGCTAAGACTACACCCACATTCGCCAATCTTGGTGAGGAATGCAACAGAAGGATGCAAGATCGGAGGGTATTACATATCGCAGAACACAACAATGATAATAAACGCCTATGCGGTGCTGAGAGATCCAGATGCATGGGAATATCCATACGAATTTCAGCCCGAGAGGTTCATGACTTCTCCTTCAGAAGGGAAAGAAGATGAAAGAGCACAGTTAGCTTTGAACTTCATTCCTTTCGGAAGTGGAAGAAGAGGATGTCCTGGAGAAAACTTGGGCTATATCTTCATAGGAGTAGCCATTGGAACAATGGTTCAGTGTTTTGATTGGAGAATCGATGGAGATAAGGTTAATATGGAAGAG---ACGGGACTGAGCATGGCACATCCACTTAAATGCACTCCTGTT

>AT4G15330_TAIR10

ATCGTCGTTGACTCTCAAAACTGCTTCATCATCATTCTTCTATGTTCCTTCTCACTCATCTCCTACTTTGTCTTCTTCAAAAAACCAAAGGTT------AACTTTGATTTGCTTCCGAGCCCTCCTTCACTTCCAATCATTGGTCATCTTCACCTCTTGCTCTCTACTCTTATCCACAAGTCTCTTCAGAAACTCTCCTCCAAGTATGGACCTCTTCTCCATCTCCGCATCTTTAACATCCCCTTCATCCTCGTCTCCTCGGACTCACTGGCCTATGAGATCTTCAGGGATCACGACGTGAACGTCTCCTCTCGCGGTGGAGCGATCGACGAGTCCCTGGCGTTTGGATCTTCTGGTTTCATACAGGCTCCCTATGGAGATTACTGGAAATTCATGAAGAAGCTCATCGCAACTAAGTTGCTGGGACCCCAACCACTGGTGCGGTCACAGGATTTTCGTTCTGAAGAGCTAGAGAGGTTCTACAAAAGGCTGTTCGATAAGGCCATGAAGAAGGAAAGCGTTATGATTCATAAGGAAGCGTCCAGATTCGTTAACAATAGCCTTTACAAGATGTGCACGGGAAGGAGCTTTTCAGTGGAGAATAATGAAGTGGAGAGAATAATGGAACTTACTGCCGACTTAGGTGCCTTGTCACAGAAGTTTTTCGTGTCAAAGATGTTTCGTAAGCTGCTTGAGAAGCTCGGTATCTCACTTTTCAAAACGGAGATAATGGTTGTTTCACGTAGATTTAGTGAGCTGGTTGAAAGGATTCTTATTGAATATGAAGAGAAACAAGGCACACAGTTTATGGATGCCTTGTTGGCAGCTTATCGAGATGAAAATACAGAATATAAAATCACTAGGAGCCATATCAAGTCGCTTCTCACGGAGTTTTTCATTGGAGCCGCTGACGCCTCATCAATAGCAATACAGTGGGCAATGGCAGATATCATAAACAACCGTGAGATTCTTGAGAAATTGAGAGAAGAAATTGATTCGGTTGTAGGAAAAACAAGATTGGTTCAAGAAACAGATTTACCAAACCTTCCTTATTTGCAAGCTGTAGTTAAGGAAGGGCTAAGATTGCACCCGCCTACACCTCTGGTTGTAAGGGAGTTCCAAGAAGGGTGTGAGATTGGAGGGTTCTTTGTACCAAAGAACACAACACTTATTGTTAATTCTTATGCTATGATGAGAGATCCTGATTCATGGCAAGATCCTGATGAGTTTAAACCAGAGAGGTTTCTAGCTTCC------TTAAGTAGAGAGGAGGATAAAAAAGAGAAAATCCTTAACTTCCTTCCTTTTGGGAGCGGAAGGAGAATGTGTCCTGGATCAAATCTTGGTTATATATTTGTAGGAACCGCGATTGGGATGATGGTCCAGTGCTTTGACTGGGAAATCAATGGAGACAAGATTAATATGGAAGAGGCTACTGGAATCACCATGGCTCATCCCCTTACATGCACTCCTATT

>AT5G42580_TAIR10

ATCATCGTTGACTTTCAAAACATATCTATCTTCATCCTCTTATGTCTCTTCTCATTCCTTTGTTACGCTCTCTTCTTCAAGAAACCAAAA---------GGCTTTGACCTGCCTCCGAGCCCTCCTTCTCTACCAATCATCGGTCATCTTCACCATCTCTCATCATCTCTACCACACAAGTCCTTTCAAAAACTCTCCTTCAAGTATGGACCTCTTCTCCATCTCCGCATCTTTAATTTCCCCATGGTCCTCGTCTCATCAGCCTCAATGGCCTACGAGGTCTTCAGGACAAACGACGTAAACGTCTCGTATCGCTTTCCAGTCAACAAAGACTCTCTAGTGTTCGGATCTTCAGGATTCGTAACCGCTCCTTATGGAGATTACTGGAAGTTCATGAAGAAGCTCATATCCACGAAACTACTCCGACCACATGCACTCGAGCTGTCGAAAGGTAACCGTGCAGAGGAGCTACGTCGGTTTTGCCTTGATCTGCAAGGTAAGGCGAGGAAGAAGGAGAGCGTTGAGATCGGTAAAGTAGCACTAAAGCTCACTAACAACATCATATGTAGGATGAGCATGGGAAGGAGTTGTTCAGAGAAGAACGGTGTAGCGGAGAGAGCAAGGGAATTGGTTAACAAATCCTTTGCCTTATCAGTGAAGCTCTTCTTTTCAAACATG------------------------------------TTCAGAAAAGATATAATGGGAGTTTCTCGTGAATTCGACGAGTTTCTTGAGAGGATTCTAGTGGAACACGAGGAGAACCAAGATAGGGACATGATTGATCATTTGTTGGAAGCTTATAGAAACGAAGAAGCAGAATATAAGATCACTAGAAAACAGATCAAGTCTTTAATTGTGGAAATTTTTCTTGGAGGCACTGACAGCTCGGCGCAGACGATACAATGGACTATGGCGGAGATACTTAACAACCCCGGCGTTCTCGAGAAATTAAGAGCAGAAATCGATTCCGTTGTGGGGGGAAAAAGGTTGATTCAAGAATCAGATCTTCCAAACCTCCCTTATTTGCAAGCTGTTGTTAAAGAAGGGCTAAGATTGCATCCCTCGGCTCCTGTCTTGTTAAGGGTATTTGGAGAAAGTTGCGAGGTCAAAGAGTTCTACGTACCGGAGAAAACAACACTTGTTGTTAATCTCTATGCTGTGAATAGAGATCCTGATTCTTGGGAAGATCCTGATATGTTTAAGCCAGAGAGGTTTTTAGTTTCTTCGATATCAGGAGATGAAGAAAAGATAAGAGAGCAAGCCGTGAAGTATGTTACTTTTGGAGGTGGAAGGAGGACATGTCCCGCGGTAAAACTAGCTCATATCTTTATGGAAACTGCGATTGGAGCGATGGTGCAGTGTTTTGACTGGAGAATTAAAGGAGAGAAAGTATACATGGAAGAGGCTGTTTCATTGAAAATGGCTCATCCGCTTAAGTGCACTCCTGTT

>AT2G14100_TAIR10

ATCGTCGTTGACTTTCAAAAAATTCATTTTCATATCCTCTTATCCATATTCACAGTCATTTGCTTTGTATTCTTCTTCAAGAAGCCAAAGGGCTCACGAGGCTGTGATCTGCCTCCGAGCCCTCCTTCTCTTCCGATAATCGGACATCTTCACCTTTTACTCTTTGATCTACCTCACAAAGCCTTTCAGAAACTCTCCTCTAAGTATGGACCTCTTCTCTGTCTCCGCATCTTCAATGTCCCCATAGTCCTGGTCTCCTCTGCCTCTGTGGCCTACGAGATCTTCAAGACGCATGACGTGAACATCTCATCCCACGGCCCTCCGATTGACGAGTGTCTCTTTTTTGGGTCTTCAAGCTTTGTAATGGCTCCTTATGGAGATTACTGGAAGTTCATGAAGAAGCTCATGGTCACAAAGCTGTTCGGACCTCAGGCACTCGAGCAGTCACGAGGCGCCCGTGCAGATGAACTAGAACGGTTTCACGCAAACCTGCTTAGTAAGGAAATGAAAAGCGAGACTGTCGAGATTGCTAAGGAAGCAATAAAGCTGACTAACAATAGCATCTGCAAGATGATTATGGGTAGGGGTTGTTTAGAGGAGAACGGTGAGGCAGAGAGAGTTAGGGGATTGGTCACCGAGACATTTGCCTTGTTTAAGAAACTTTTCTTGACACAAGTGTTACGCAGGCTGTTTGAGATACTCAGAATCTCACCGTTCAAAAAAGAGACACTGGATGTTTCCCGCAAATTCGACGAGCTTCTTGAAAGGATTATTGTGGAACACGAAGAGAAACATGGTATGGACCTGATGGATGTGCTGTTGGCTGTTTATCGAGATGGAAAGGCAGAGTATAAGATCACTAGGGACCACCTCAAGTCCTTGTTCGTGGAGCTTATCCTTGGAGGCACTGACACCTCAGCGCAAACAATCGAGTGGACAATGGCAAAGATCATTAAGAAGCCTAACATTCTTGAGAGATTGAGAAAAGAAATCGATTCTGTTGTAGGCAAAACAAGGTTGATTCAAGAGAAGGATCTACCGAACCTCCCTTATTTGCAAGCGGTCATCAAGGAAGGGCTAAGATTACACCCACCAGCACCTCTCTTGGGAAGGAAAGTCACAGATGGATGTACGATTGGAGGCTGTTACGTACCAAAGAACACAACACTTGTTGTTAATGCTTATGCCGTGATGAGAGATCCCGATTCTTGGGAAGATCCTGATGAGTTTAAGCCAGAGAGGTTTCTAGCTTCTTCAAGA------GGAAAAGAAGAGGAGAGAGAGCAAGAACTTAAGTACATTCCTTTTGGCAGCGGAAGAAGAGGATGTCCTGGAGTAAATCTAGGTTATATATTTGTAGGAACCGCAATAGGAATGATGGTGCATTGCTTTGACTGGAGAACCAATGGAGATAAGGTCAACATGGAAGAGACTGTTGCTTTAAACATGGCTCATCCTCTTAGGTGTACTCCTGTT

>AT3G32047_TAIR10

ATCGTCGTTGACTTTCAAAATTGCCTCATTTTCATTCTTATATCCTTATTTTCTCTCCTTTGCTTTGTATTCGTCTTCAAGAAGCCAAAGGACTCACGAGGCTGTGATCTGCCTCCGAGCCCTCCTTCTCTTCCGATAATCGGTCATCTTCACCTTATACTCTCTACTCTACCTCACAAGTCCTTTCAAAATATATCCTCCAAGTATGGACCTCTTCTCCTTCTCCGATTTTTCAATGTCCCCGTTGTCCTCAAATCCTCTGCCAATGTGGCCTACGAGATCTTCAAGACGCACGACGTGAACATCTCGTCCCATGGCCCTCCGATTGACGAGTGTCTCTTTTTTGGGTCTTCTAGCTTTGTAGTGGCTCCTTATGGATATTACTGGAGGTTAATGAAGAAGCTCATGGTCACAAAGCTGTTCGGACCTCAGGCACTCGAGCGGTTACGACACGTCCGTGAAGATGAACTAGAAAGGTTTCACACAAACCTGCTTAGTAAGGAAATGAAAGGCGAGACTGTCCAGATTGCTAAGGAAGCAATAAAGCTGACTAACAATAGCGTCTGCAAAATGATCATGGGTAGGAGTTGTTTAGAGGAGAATGGTGATGCAGCAAGAGTTAGGGGATTGGTCACCGAGACATTTGCCTTGGTTAAGAAAATCTTCTTGACACAAGTGTTGCGTAGGCTGTTTGAAATACTCGGAATCTCACTGTTCAAAAAAGAGATATTGGGTGTTTCCCGCAAATTCGACGAGTTTCTGGAAAAGATTCTTGTGGAACACGATGAGAAACAAGGTGGAGACATGATGGACGTGTTGTTGGCAGCTTATCGAGATGAAAATGCAGAGTATAAGATCACTAGGAACCATATCAAGTCGTTGTTCGCGGAGCTTATCCTTGGAGGCACTGACACCTCAGCGCAGACAATTGAGTGGACAATGGCAGAAATCATTAATAAGCCTAACATTCTTGAGAAATTGAGAAAAGAACTCGATTCTGTTGTAGGAAAAACAAGGCTGATTGAAGAGAAGGATCTACCAAACCTCCCTTATCTGCAATCGGTCGTCAAGGAAGGGCTAAGATTGCACCCACCAGCGCCTGTGTTTGGAAGGAAAGTCCTAGAAGGATGTACGATTAAAGGCTATTATGTACCAAAGAACACAGCTCTTGTTGTTAATGCTTATGCTGTGATGAGAGATCCCCATTATTGGGAAGATCCTGATGAGTTTAAGCCAGAGAGGTTTTTAACTACTTCAAGC------AAAAAAGAAGAGGAGAGAGAGCAAGAACTTAAGTACATTCCTTTTGGCAGCGGAAGGAGAGGATGTCCTGGAGTAAATCTAGGTTATATATTTGTAGGAACCGCAATAGGAATGATGGTGCATTGCTTTGACTGGAGAGTCAAAGGAGATAAGGTCAACATGGACGAGACTGCTGCTTTAAACATGGCC---------------------GTG

>AT4G15380_TAIR10

AACATGATTGACTTTCAAAACTGCTTCATCTTTATCCTCCTATGTCTCTTCTCACGACTCTGTTATTCTCACTTCTTCAAGAAACCAAAGGACCCTCGCCACTTTGATTTACCTCCGAGCCCTCCGTCTCTTCCCATCATCGGTCATCTGCACCTTCTTCTCTCTGTTCTACTCCACAGATCTTTACAAAAACTCTCCACCAAGTACGGATCTATTCTCTATCTCCGAGTCTTTAGGTTCCCTGTAGTCCTCATCTCGTCAGCCTCAATTGCATATGAGATCTTCAGGGCACACGACCTGAACATCTCGTACCGCGGTACTCCGACCGATGATTCACTTTTTGCCGGATCTTTCAGCTTTATCTCTGCTCCCTATGGAGATTATTGGAAGTTCATGAAGAAGGTCCTAGTTACAAACGTTTTTGGGCCCCAAGCACACGAGCAGTCACGAGGTGTCCGTGCAGATGTGCTAGAGCGGTTTTATGGGAATCTGTTCGATAAAGCGATAAAGAAGCAGAGCGTGGAGATATGTGCTGAAGCGTTGAAGTTATCTAACTCCAGCATCTGCAAGATGATCATGGGGAGGAGTTGTTCAGAG---------------GAGAGATTCAGGGCTTTGGCTACCGAGTTAGATGTTTTGACAAAGAAGCTCTTCTTTGCAAACATGTTGCGAGCATGGTTTAAGAAGCTTGTTGTCTCTCTGTTTAAAAAAGAAACTACAGTTATTTCCTACAGATTCGATGAGCTGCTCGAAAGCATTCTTGTGGAACACGAAAAGAAACAACGTACGGACTTGATGGACGCATTGTTGGCAGCTTATCGGGATGAAAATGCAGAGTATAAGATCACAAGGAACCATATCAAGTCAATTATTGCGGATCTTTTATTTGCAGGCACTGAAAACCAAGTGCAGACAATACAATGGGCAATGGCAGAGATCATTAACAACCCTAACGTTCTTGAGAGACTGAGAGGAGAAATCGATAGCGTGGTAGGAAAGTCAAGGTTGATTCAAGAAACGGATTTACCAAAGCTCCCTTATTTGCAAGCTGTGGTTAAGGAAACGATAAGACTGCACCCGCCGGGGCCTTTCTTCTTAAGGTTCACAAAAGAAGGGTGTAGGATCAGAGGGTTCTATGTACCAGAGAACACGTCAGTCGTTGTTAACGTTTATGCTGTGATGAGGGATCCTGATGCTTGGGAGGATCCTCTTGTGTTTAAGCCAGAGAGGTTCTTAGCTTCTTCAAGAGCAGAACAAGAGGAGGAGAGAAGAGAGAAAGAAATTAAGTACCTTCCTTTTGGAAGCGGAAGAAGAAGTTGTCCCGGAGAAAATCTAGCTTATGTCATTATGGGAACTGCAATTGGAGTGATGGTGCAGGGTTTTGAGTGGAGAACCACAGAAGAGAAAATTAACATGGACGAGGCTGTTGTATTGACCATGGCTCATCCTCTTAAGATCATTCCTGTT

>AL3G33780_v2_1

ATCATCGTTGACTTTCAAAACTGCTTCATTTTTATCCTCTTAAGCCTCTTTTCGCTACTCTGTTACTCTCTCTTCTTTAGGAAACCAAAAGAACCTCGACACTTTGATTTACCTCCGAGCCCTCCGTCTCTTCCGATCATCGGTCATCTTCACCTTCTTATCTCTGTTCTACTCCACAGATCTTTACTGAAACTCTCCATCAAGTACGGATCTATTCTCTATCTTCGCGTCTTCAGTTTCCCTGTAGCCCTCATCTCCTCAGCATCAATTGCTTATGAGATCTTCAGGGAACACGACGTTAACATCTCGTCTCGTGGTCCTTCGACCGATGATTCCCTTTTTGCCGGATCTTTCAGCTTCACATCTGCTCCCTATGGAGATTACTGGAAGTTCATGAAGAAGCTCCTGGTTACAAACTTGCTTGGAGCCCAGGCACTCGAGCGGTCACGAAGAGTCCGTGCAGATGAGCTAGATCGGTTTTACAAGAACCTGTTGGATAAAGCGATGAATAAAGAGAGCGTGGAGATATGTGCGGAAGCGTTGAAACTCAGTAACAACATCATCTGCAAGTTGATCATAGGGAGGAGTTGTTCAGAGGAGAATGGTGAGGCGGAGAAAGTCAGGGCTTTGGCTACCGAGTTAGATGGCTTGACGAAGAAGATCTTATTGGCAAACATGTTGCGTCCAGGGTTTAAAAAGCTTGTTGTCTCACTGTTTAGAAAGGAAATGATGGATGTTTCCAGCAGATTTGATGAGCTGCTCGAAAGGATCCTTGTGGAACATGAAGATAAACAAGGTACAGACTTAATGGACGCGCTGTTGGCAGCTTATCGGGGCAAAATTGCAGAGTATAAGATCTCTAGGAACCATATCAAGTCATTTTTCGCGGATCTCTTATTTGCAGGCACCGACACCTTGGTGCAAACAACACAGTGGGCAATGGCGGAGATCATGAACAACCCTAATGTTCTTGAGAGATTGAGAGGAGAAATTGATTCCGTGGTAGGAAAGAAAAGGTTAATTCAAGAAACTGATTTACCAAACCTTCCTTATTTGCAAGCGGTGGTTAAGGAAGGACTAAGACTGCACCCTCCTGGTCCTCTCTTTGGAAGGTTCTCCCAAGAAGAGTGTAGGATGGGAGGGTTTTATGTACCGGGAAAAACAATAGTTATGGTTAATGCTTATGCTGTGATGAGAGATTCTGATTCTTGGGAATTTCCTGATGATTTTAAGCCAGAGAGGTTTCTAGATTTGTCAAGATCAGAGCAGGAAGAGGATAGAAGAGAGCAAGCAATTAAGTACATTGCTTTCGGAAGTGGAAGAAGAAGCTGTCCGGGAGAAAATCTAGCATATATCTTTATAGGAACTGCAATTGGAGTGATGGTACAGGGATTTGAGTGGAGAATCAAAGAAGAGAAAATTAACATGGAAGAGGCAGTTGTATTGACCATGGCTCATCCTCTTAAGATCATTCCTATT

>AT3G20080_TAIR10

AACATCGTTGACTTTCAAAACTGCTTCTTTTTTGTCCTCTTAAGCCTCTTCTCACTTCTCTGTTACTCTCTCTTCTTCAGGAAACCAAAGGAACCTCGACACTATGATTTACCTCCGAGCCCTCCGACTCTTCCCATCATTGGTCATCTTCACCTTCTTCTCTCTGTTCTAGTCCACAGATCTTTACAGACACTCTCCACCAAGTATGGGTCTATTCTCTATCTTCGCGTCTTCAGTTTCCCTGTAGCCCTCGTCTCCTCAGCATCTATTGCTTATGAGATCTTCAGGGAACACGACGTGAACATCTCGTCTCGTGGTCCTCCTACCGATGATTCCCTTTTTGCTGGATCTTTCAGCTTCACCTCTGCTCCCTATGGAGATTACTGGAAGTTCATGAAGAAGCTCCTGGTCACGAACTTGCTTGGACCCCAGGCACTCGAGCGGTCACGAGGATTCCGTGCAGATGAGCTAGATCTGTTTTACGAGAACCTGTTGGATAAAGCGATGAAGAAGGAGAGCGTGGATATCTGTGTGGAGGCGTTGAAGCTCAGTAACAACAGCATCTGCAAGATGATCATGGGGAGGAGTTGTTCAGAGGAGAATGGTGAGGCAGAGAGAGTTAGGGCTTTGGCTACCCAGTTAGATGGCTTGACGAAGAAGATCTTATTGGCGAACATGTTGCGTGCGGGGTTTAAAAAGCTTGTTGTCTCACTGTTTAGAAAGGAAATGATGGATGTTTCCAGCAGATTTGATGAGCTGCTCGAAAGGATCCTTGTGGAACATGAAGATAAACAAGGTACAGACTTAGTGGACGCGTTGTTGGCAGCTTGTCGGGACAAAAATGCAGAGTATAAGATCTCTAGGAACCATATCAAGTCGTTTTTCGCGGATCTCTTATTTGCAAGCACTGACACCTTTGTGCAAACAACACAGTGGACAGTGGCGGAGATCATTAATAACCCTAATGTTCTTGAGAGATTGAGAGGAGAAATCGATTCCGTGGTCGGGAAAGCAAGGTTGATTCAAGAAACTGATTTGCCAAACCTCCCTTATTTGCAAGCGGTGGTCAAGGAAGGGCTAAGACTGCATCCGCCGGGGCCTCTCTTTGCAAGGTTCTCCCAAGAAGGGTGTAGGATCGGAGGGTTCTATGTACCGGAGAAAACAACATTAATGATTAATGCTTATGCTGTGATGAGAGATTCTGATTCTTGGGAAGATCCTGATGAGTTTAAACCAGAGAGGTTTCTAGCTTCGTCAAGATCAGAGCAGGAAAAGGAGAGAAGAGAGCAAGCAATTAAGTACATTGCTTTCGGAAGCGGACGAAGAAGCTGTCCTGGAGAAAATCTAGCATATATCTTTTTAGGAACTGCAATTGGAGTGATGGTACAGGGATTTGAGTGGAGAATCAAAGAAGAGAAAGTTAACATGGAAGAGGCTAATGTATTGACCATGGCTTATCCTCTTAAGGTCACTCCTGTT

>AT3G20110_TAIR10

TTGACCGTTGACTCTCAACACTGTTTCAGTTTCATACTCCTCTGCTTCTTCTCACTCCTCTGTTACTCTCTCTTGTTCAAGAAACTAAAGGACTCACATGGCCGTGATTTGCTTCAGAGCCCTCCATCTCTTCCGATCATAGGCCATCTTCATCATCTCCTCTCTTCTCTAGCACACAAATCTTTGCAGCAACTCTCATCCAAGTATGGACCTCTCCTACATCTCAGCATCTTCAACTTCCCTGTAGTCCTCGTCTCCTCGGCCTCTGTGGCTTACGAGATCTTCAAGGCTCATGACTTGAACATCTCGTCTCGCGACCCTCCAATCAATGAGTCCCTCTTGGTCGGTTCTTCTGTGTTCGTCGGCGCCCCCTATGGAGATTATTGGAAGTTCATGAAGAAGCTCTTGGTTACAAAGCTGCTGGGACCACAGGCACTCGAGCGGTCAAGAAGCATCCGTGCAGATGAGCTAGAGCGGTTTTACAGAAGCTTGCTCGATAAGGCGATGAAGAAGGAGAGTGTTGAGATTGGTAAGGAAGCAACGAAGCTAAGTATCAACAGCATATGCAGGATGAGCATGGGGAGGAGTTTTTCAGAAGAGAGTGGTGAGGCTGAGAGAGTTAGAGGTTTGGTTACCGAGTTAGATGGCTTGACGAAGAAGGTTTTGTTGGTAAACATATTGCGCTGGCCGCTTGAGAAGCTCAGAATCTCTCTGTTCAAAAAAGAGATAATGTATGTTTCGAACAGCTTTGATGAGCTGCTGGAGAGGATTATTGTGGAACGTGAAAAGAAACAGGGTACATACTTGATGGACGTGTTGTTGGAAGCTTATGAAGACGAAAAAGCAGAGCATAAGATCACTAGGAACCATATCAAGTCGTTGTTCGTGGAGCTTTTACTTGGAGGCACTGATACCTCGGCGCAAACAATACAGTGGACAATGGCCGAGCTCATCAACAACCGTAACGTTCTTAAGAGATTGAGAGAAGAAATTGATTCGGTTGTAGGAGAAACAAGGTTGATTCAAGAAAAGGATCTACCAAAGCTACCTTATTTACAATCTGTAGTCAAAGAAGGGCTAAGATTGCACCCGCCATTGCCTCTCATGGTAAGGACGTTCCAAAGAAGTTGTGAGATGAAAGGGTTCTACATAGCGGAAAAGACAACACTTGTAGTTAATGCTTATGCTGTGATGAGAGATCCTACTACTTGGGAAGATCCTGATGAGTTTAAGCCAGAGAGGTTTCTAAGG---------------CAAGAGGAGGAGAGAAGA------GCACTGAAGCACATTGCTTTTGGAAGTGGAAGGAGAGGCTGTCCAGGATCAAACCTAGCTACTATTTTCATAGGAACTGCAATTGGAACAATGGTGCAGTGTTTTGACTTGAGCATCAAAGGAGACAAGGTCAAAATGGATGAG---GTTGGATTGACCATGGCTCATCCCCTTGAGTGCATTCTTGTT

>AL3G33800_v2_1

ATCATCGTTGACTTCCCAACCTGCTTCATATTTATCCTCTTATGCCTCTTCTCACTCCTCTTCTACTATCTCATCTTCAAGAAACCTAAGAAATCAAGGGGATGTGAACTGCCTCCGAGCCCTCCATCTCTTCCGATTATAGGCCATGTTCACCTGCTCCTCTCTTCCCTAGCTCACAAATCTTTACAGAAACTCTCATCCAAGTATGGACCTCTTCTTCATCTCCGCGTCTTCAACTTCCCTGTAGTCATCGTCTCCTCGGCTTCTATGGCCTACGAGATCTTTAAGGTTCATGACTTGAACATCTCGTCTCGTGATCCTTCAATTGACGATTCCCGCTTGATTGGATCTTCTGTCTTTGGTAGTGCTCCTTATGGAGATTACTTTAAGTTCATGAAGAAGCTCCTGGCTACTAAGCTGCTTGGACCGCAGGCACTTGAGCGGTCACGAGGCATCCGTGCAGATGAGCTTGAGCGGTTTCACTCAAGCCTGCTTGATAAGGCGATAAAGAAGAAGAGTGTTGAGATTGGTAAGGAAGCAACGAAGCTCAGTATTAACAGCCTCTGGAGGATGAGCATTGGGAGAAGTTTTTCAGAGGAGAATGGTGAGGCTGAGAGAGTCAGGGGACTGGTTACCGAGTTAGATGGCTTGACAAAGAAGGTTTTGTTTGCAACTTTGCTGCAGAAACCCCTTGAGAAGCTTGGAATCTCTCTTTTCAAAAAGGAGATCATGTCTGTTTCCAACAGCTTCGATGAGGTCCTGGAGAGGGTTCTTGTGGAACATGAACAGAAACAAGATAGGGACATGGTGGACGTGTTGTTGGCAGCTTATGGAGATGAAAACGCAGAGCATAAAATCACCAGGAACCACATCAAGGCATTTTTCGTGGAGCTTTTCTTTGCGGGTACTGACACCTCGGCGCAATCAATACAGTGGACAATGGCAGAGATCATTAACAACCCCAAGATTCTTGAGAGATTGAGAGAAGAAATTGATTTTGTGGTAGGAAAAACAAGGATGATTCGAGAAACTGATCTACCAAAGCTCCCCTATTTGCAAGCGGTGATTAAGGAAGGACTGAGATTGCACCCGCCATTGCCTCTCTTTGTTAGGACGTTCCAAGAAGGGTGTAAGATTGGAGGTTTCTACGTACCAGAGAAGACAACACTTATTGGTAATGCTTATGTTATGATGAGAGATGCGAATGTATGGGAAGACCCTGAGGAGTTTAAACCAGAGAGGTTTCTAGCTTCTTCAAGATTAGGGCAAGACGAGGAAAGAAGAGAGCAAGCGCTTAAGTACATTCCTTTCGGGAGTGGAAGGAGAGGTTGTCCTGGCTCAAATCTGGGTTATATCTTTATTGGAACCGCAGTTGGATTGATGGTGCAGTGCTTTGACTGGAGAATCAAAGGAGACAAGGTTAACATGGATGAG---GCTGGGTTAACCATGGCTCATCCCCTGAAGTGCACTCCTGTT

>AT3G20100_TAIR10

ACCACCACTGAGTTTCAAAGCTGCTTCATCTTCCTCCTCTTATGTCTCTTCTCACTCGTCTGTTACTCTCTCTTTTTCAGGAAACCAAGCTCAAGACGGGGCTGTGATCTGCCTCCGAGCCCTCCTTCTTTGCCAGTCATCGGCCATCTTCACCTTATCCTCTCTTCCCTTGTCCACAAGTCTTTTCAAAAGATCTCCTCCAACTACGGACCTCTCCTCCATCTCCGCATCTTCAACGTTCCCATAGTCCTCGTCTCCTCTGCGTCAGTGGCCTACGATATCTTCAGGGTGCATGACTTGAACGTCTCTTCTCGCGGCCCTCCGTTTGAGGAGTCACTGTTGTTCGGATCTACCGGCTTCATTAGTGCTCCCTATGGAGATTATTTCAAGTTCATGAAGAAACACCTGGTCACAAAGCTCCTTGGACCACAGGCACTCGAGCGCTCACGCCTCATCCGGACAAATGAACTAGAACGGTTTTACATAAACCTGCTTGATAAGGCGACGAAGAAGGAGAGCGTTGAAATTGGTAAGGAAGCGATGAAGCTCAGTAACAACAGCATCTGCAAGATGATCATGGGGAGGAGTTGTTTAGAGGAAAAGGGTGAGGCAGAGAGAGTCAGGGGTTTGATTATCGAGTCGTTTTACTTGACGAAGAAATTTTTCTTGGCATTCACTTTGCGCGGGCTGCTCGAGAAGCTTGGAATCTCGCTGTTCAAAAAAGAAATAATGGGAGTTTCCCGCAGATTTGATGATTTGCTCGAAAGGTATCTTAGGGAACATGAAGAGAAACAAGATACGGACATGATTGATGCACTATTGGCAGCTTATCGAGACGAAAAGGCCGAGTATAAGATCACTAGGAACCAGATCAAGGCGTTTTTAGTGGATATTTTCATTGCGGGCACTGACATCTCGGCGCTAACAACGCAGGGAACAATGGCTGAGATCATTAACAACCCTAACATATTTGTGAGAATTAGAGAAGAAATCGATTCTGTTGTAGGGAAATCAAGGCTGATTCAAGAAACGGATCTACCAAAGCTCCCTTACTTGCAAGCGGTAGTCAAAGAAGGGCTTAGATTGCACCCGCCTACGCCTCTCATGGTAAGGGAGTTCCAAGAAGGGTGTAAGGTCAAAGGTTTCTACATACCAGCGAGCACAACCCTTGTTGTTAATGGTTATGCTGTGATGAGAGATCCAAATGTCTGGGAAGACCCTGAGGAGTTCAAGCCGGAGAGGTTTTTAGCTTCTTCAAGATTAATGCAAGAGGACGAGATAAGAGAGCAAGCCCTCAAGTACATCGCTTTCGGTAGTGGAAGGAGAGGCTGCCCAGGAGCAAATGTAGCTTATATCTTTGTAGGAACCGCGATTGGAATGATGGTGCAATGCTTTGACTGGAGAATCAATGGAGAAAAGGTTGACATGAAAGAGGCCATTGGACTGACCTTGGCTCATCCCCTTAAGTGCACTCCTGTT

>AL3G33760_v2_1

ATCATCGTTGACTCTCAAAACTGCTTCATCTTCATTCTCTTATGTCTCTTCTCACTTCTCTGTTACTATCTCTTCTTCAAGAAACCTAAGTAT------GGCTTTAACTTGCCTCCGAGCCCTCCTTCTCTGCCCATCATTGGTCATTTTCACCTTATTCTCTCTGTTCTAATCCACAAGTCCTTTCAGAAACTCTCCTTCAAGTACGGACCATTACTCTATCTCCGCATCTTCAATGTCCCCATAGTCCTTGTCTCCTTTTCCTCGATAGCCTATGAGATCTTCAAGACACATGACGTGAACATCTCGTTCCGTGGCCCTCCCATAGATGAGTCCCTCTTGTTCGGATCTTCCACCTTCCTCATGGCTCCTTATGGAGATTACTGGAAGTTCATGAGGAAGCTCATCGTCACGAACCTGCTCGGCTCCCAAGCACTCGAACGGTCACGAGTCATCCGTAAAAATGAGCTTGAGTGGTTTTATGCAAGCTTGCTTGATAAGGCGATGAAGAATGAGAGCGTTGAGATCGGTAAAGAATCGATGAAGTTTAGTAACAACTGCATTTGGAAGATGAGCATGGGAAGAAGTTGTTGTGAAGAGAACAGTGAGGCAGAGAGAGTCAGGGGTTTGGTTACAGAGTCATTTGCCTTGTTTAAGAAAATCTTCTTGGCAACATTGCTTCGTAGGCCGCTCGAGAAGCTTGGAATCTCACTATTTAAAAAGGAAATATTGAGTTCTTCCCGTAGATTCGACGAGCTGCTAGAGAGGATTCTTGGAGAACACGGAGAAGAGCAGGGTGGAGACATGATGGACGTATTGTTGGCAGCTTTTCGCGATGAAAATGCAGAGTATAAGATAACTAGGAATCATATAAAGTCTTTTTTCATGGAACTTTTCATTGCAGGCACTGACAGTTCAGGGCAGAGTACACAGTGGACAATGGCCGAGATCATTAATAACCCTATTTGTCTAAAGAGACTAAGAGAAGAAATCGAATCGGTTGTAGGGAAAACAAGGTTGATTCAAGAAACGGATTTACCAAACCTTCCTTACTTGCAAGCGGTGGTCAAGGAAGGGCTTAGATTACATCCACCAGCTCCTCTATTTGCAAGGACCTCCAGAGAATGGTGTGAGATTAGAGGGTTCTATATACCAGAAAACACAACACTTGTTGTTAATGCTTATGCTATTATGAGAGATCCTGATTCTTGGGAAGATCCTAATGAGTTTAAGCCAGAGAGGTTTTTAGCTTCTTTTGAATCAGGGCAAGAAGAT---------------GCACTTAAGTACATTCCTTTCGGAAGCGGAAGGAGAGGCTGTCCGGGAGTAAATCTGAGTTACATCTTTGGAGGAACCGCGGTTGGAATGATGGTGCAATGCTTTGACTGGAAAATCAAAGGAGAAAAGGTTGACATGGAAGAGGCCATTGGACTGACCATGGCTCATCCCCTTAAGTGCATTCCTGTT

>AL3G33810_v2_1

GTAATCATTGAATATCAAAACTACTTCATATTTGTCCTCCTCTGCTTCTTTTCACTCCTCTTTTACCCTCTCTTCTTCAAGAAACCAAAGGACTCACAAGGGTTTGATTTGCCTCCAAGCCCTCCTTCTCTGCCAATCATTGGTCATCTTCACCTGCTCCTCTCTACTCTAACCCACAAGTCCTTACAGAAACTCTCCTCCAAGTATGGACCTTTCCTCCATCTCCGTATCTTCAATGTCCCTGTAATCTTTGTTTCCTCGGCTTCTGTAGCATACGAGATCTTCAGGGCACATGATGTGAACATCTCTTTTCGCGGCCCTCCTGTCAAAGAGTCCCTCTTGGTTGGATCTTCTGGCTTTTTCACCGCTCCTTATGGCAATTATTGGAAGTTCATGAAAAAGCTAATGGTCACGAAGCTGCTCGGACCGCAGGCACTCCAGCGGTCACGAGGCATCCGTGCAGATGAGCTAGAACGGTTTTACAAGCACCTTCTTGATAAGGCAAGGAAGAACGAGAGCATTGAGATTGGTAAGGAAGCAATGAAACTCATTAATAACAGCATCTGCAAGATGTTTATGGGGAGGAGTTGTTCTGAGGAAAATGGTGAGGCAGAGAGAGTCAGAGGCTTGGTGACCGAATCGACTGCCTTGACAAAGAAGATCTTTATGGCAAACATGTTGCACAGGCACCTTAAGAAGCTCGGAATATCACTGTTCAAAAAGGAGATAATGGGTGTTTCTTGCAGATTCAACGAAGTGCTGGAGAGGATTCTTGAGGAATACGAAGAGAAA------------------------------GCAGCTTACCGAGACAAAAATGCAGAGTGTAAGATCACTAGGAACCACATTAAGTCGTTGTTCGTGGATCTTGTCGTTGCAGGCACCGACACTTCAAGACATGCAACACAGTGGACAATGGCAGAGATAATTAACAAGCCTGCGATTCTTGATAAATTAAGAGAAGAAATCGATTCTGTTGTAGGGAGAACAAGGTTGGTTCAAGAAACAGATTTACCAAGCCTACCATATTTGCAAGCTATTGTTAAGGAAGGACTGAGATTGCACCCTCCCGGGCCTCTCTTTGCAAGGACAGCTCGAGAAGGGTGTAGGGTCGGAGGATTTTATGTACCCCAGAACACACCACTTGTTGTTAATGCTTATGCTATGATGAGAGATCCTGATACTTGGGAAGAGCCCAATGAGTTTAAGCCAGAGAGGTTTCTAGGTTCAGGAAAA------------GAAGAGGAGAGAGAGCATGGACTTAAGTACATTCCTTTTGGTAGCGGAAGAAGAGGCTGTCCTGGAGTAAATCTAGCTTACATTCTTGTTGGAACGGCGATTGGAGTGATGGTACAGTGCTTTGATTGGAAAATCAAAGGAGATAAAGTTAACATGGAAGAGGCTCGTGGATTGACCATGGCTCATCCCCTTAAGTGCATTCCTGTT

>AT3G20140_TAIR10

TTGACTGTTGAGTTTCAAAACTGCTTGATCTTTATCCTCCTCTGCATCCTTTTACTCCTATGTTACCCTCTCTTTTTCAAGAAACCAAAGGTCTCACAAGGATTTGGTTTGCCTCCAAGCCCTCTATCTCTGCCAATCATTGGTCATCTTCACCTGCTTTTCTCTAATCTAACTCACAAGTCCTTACAGAAACTCTCCTCCAAGTATGGACCTCTCCTCTATCTCCGCATCTTCAATGTCCCGATAATCTTTGTTTCCTCGGCCTCAGTGGCATATGAGATCTTCAGGGGACATGATGTGAACATCTCTTTTCGAGGGCCTCCTATCGAGGAGTCCCTTTTGGTCGGATCTTTTGGATTTTTCACCGCTCCTTATGGAGACTATTGGAAATTCATGAAGAAGGTCATGGTCACGAAACTGCTCGGACCGCAGGCACTACAAAGGTCTCGAGGCATCCGTGCAGATGCGCTAGAACGGTTTTACATGAACCTGCTTGATAAGGCGATGAAGAAGGAAAGTGTTGAGATTGGTAAGGAAACAATGAAGCTCATTTACGACAGCATCTGCAAGATGATTATGGGGAGGAATTTTTCTGAGGAGAATGGTGAGGCGGAGAGAGTCAGAGGCTTGGTGACCGAATCGACTGCCTTGACGAAGAAGATTTTTATGGCAAACGTGTTGCACAAGCCCCTTAAGAAGCTCGGAATCTCATTGTTCAAAAAGGAGATAATGGATGTTTCCAACAGTTTCGATGAACTGCTAGAAAGATTTCTTGTGGAACACGAAGAGAAACAAGATATGGACATGATGGGCGTGTTGTTGGCAGCTTGCCGAGACAAAAATGCAGAGTGTAAAATCACTAGGAACCACATTAAGTCGTTGTTCGTGGATCTTGTTGTTGCAGGCACCGACACTTCGAGACATGCAACACAGTGGACAATGGCAGAGATAATTAACAAGCCTAAGGTTCTTGAAAAGGTAAGAGAAGAAATTTATTCAGTTGTAGGAAGAACAAGGTTGGTTCAAGAAACAGATTTACCGAGCCTACCTTATTTGCAAGCTACTGTCAAGGAAGGACTGAGATTGCACCCTCCCGGGCCTCTCTTTGCAAGGACAGCCCGAGAAGGGTTTAGTGTCGGAGGATTTTATGTACCAGAGAACACACCACTTGTTGTTAATGCTTATGCTATGATGAGAGATCCTGGTTCTTGGGAAGATCCCAATGAGTTTAAGCCAGAGAGGTTTCTAGGTTCAGGAAAA------------GAAGACGAGAGAGAGCACGGACTTAAGTACATTCCTTTTGGTAGCGGAAGGAGAGGCTGTCCTGGAATAAATCTAGCTTACATTCTTGTTGGAACCGCGATTGGAGTGATGGTACAGTGCTTTGACTGGAAAATCAAAGGAAATAAGGTTAACATGGAAGAAGCTCGTGGATTAACCATGGCTCATCCACTTAAGTGCATTCCGGTT

>AT2G27000_TAIR10

ATGAACGTTGACTTTGTAAACTGTTTGATCTTAATCCTCCTCTGCCTCCTTTCAATCCTCTGTTACTCTTTCTTCTTCAAGAAACCAAAGGAT------GGTTTCAATTTGCCACCGAGCCCTCCCTCTCTACCGATCATTGGCCATCTTCATCATCTCCTCTCTCTTTTTATGCACAGATCTTTGCAAAAACTCTCCTCCAAGTATGGACCTCTCCTCTATCTTCACGTCTTCAATGTTCCCATACTCCTTGTCTCCTCTCCCTCAATAGCCTATGAGATCTTTAGGGCACAAGACGTAAACGTTTCCACTCGCGACCCTACGAATGAGGGGTCTCTCTTCCTCGGATCATTTAGCTTCATTACCGCACCTTACGGAGAATACTGGAAGTTCATGAAGAAGCTCATCGTCACAAAGCTCCTCGGGCCTCAAGCACTCGAGAGGTCACAACGCATCCGTGCAAATGAAGTAGAGAGGTTTTACTCAAACCTATTAGATAAGGCGATGAAGAAAGAAAGTGTTGAGATTGCTGATGAAGCTATGAAGCTAGTCAACAACATAATCTGCAAGATGATTATGGGGAGGACCTGTTCAGAAGAAAATGGTGAAGCAGAGAGAATTAGAGGCTTGGTGACCAAGTCAGATGCCTTGTTGAAGAAGTTTTTGCTGGCGGCCATCTTGCGGAAACCACTTAAGAAGATTGGGATCACACTGTTTAAAAAGGTGTTCATGGATATTTCTCTCAAGTTTGACGAGGTGTTAGAGAAGATTCTTGTGGAAAACGAAGAGAGACAAGGTACTGACATAATGGATAAGCTCTTAGAAGTTTATGGAGACAAAACTTCCGAGTATAAGATCACTAGAGACCATATCAAGTCCTTGTTTGTGGATCTTTTCTTTGCAGGTACTGACACCGCAACACACACTATAGAGTGGACCATGGCCGAGATCATGAACAACTCTTTGATTCTTGAGAGATTGAGAGAAGAAATTGATTCTGTTGTAGGGAAAACAAGGTTGATTCAAGAAACTGATCTACCGAATCTCCTTTACTTGCAAGCAACTGTCAAAGAAGGGCTAAGATTGCACCCTACGATCCCTCTTGTGTTGAGGACGTTTCAAGACGGGTGTACGATCGGAGGCTTTTCTATACCTAAGAAGACAAAACTTGTTGTTAATGGTTATGCTATAATGAGAGATCCTGATAACTGGGAAGATCCACTGGAGTTTAAACCAGAGAGGTTTCTAGCTTCTTCAAGATCAAGCCAAAAAGACGCAATAAAAGAAGAAGTTCTAAAATATCTTTCATTCGGAAGCGGAAGGAGAGGATGTCCTGGAGTGAATCTAGCTTATGTGTCTGTAGAAACCGCTATTGGAGTGATGGTGCAATGCTTTGATTGGAAGATCGACGGACATAAGATCAACATGAATGAGGTTGCTGGTTTGAGCATGGCTCATCCTCTTAAGTGCACTCTTGTT

>AT2G27010_TAIR10

ATGAACGTTGACTTTCAAAACTGTTTGATCTTAATCCTCCTCTGCCTCCTTTCCTTCCTCTGTTACTCTTTCTTCTTCAAGAAACCAAAGGAT------GGTTTCAATTTGCCACCGAGCCCTCCCTCTCTACCGATCATTGGCCATCTTCATCATCTCCTCTCTCTTTTTATGCACAGATCTTTGCAAAAACTCTCCTCCAAGTATGGACCTCTCCTCTATCTTCACGTCTTCAATGTTCCCATACTCCTTGTCTCGTCTCCCTCAATAGCCTATGAGATCTTTAGGACACAAGACGTAAACGTTTCCTCGCGCGACCCTACGAACGAAGGGTCTCTCCTCTTCGGATCCTTTGGCTTCGGCACCGCACCT------------------------------------------------------TCCTCGGGCCTCAAGCACTCGAGAGGTCACAAAAAGTCCGTGCAGAGGAGTTATTACTTAAACCTATTAGATAAGGCGGTGAAGAAGGAGAGCGTTGAGATTGCTGAGGAAGCTATGAAGCTGGTCAACAATACCGTTTGCCAGATGATTATGGGGAGGAGTTGTTCAGAGGAAAACGGTGAAGCAGAGAGAGTCAGAGGCTTGGTGACCAAGACAGATGCCTTGACGAAGAAGTTTATCTTGGCAGGCATCTTGCGGAAACCGCTTCAGAAGATTGGGATCTCACTGTTCAAAAAGGAGTTAATGGATGCTTCCTGCAAGTTCAACGAGGTGTTGGAGAAGATTCTTGTAGAATATAAAGAGAAACAAGGTACTGACATGATGGATAAGCTCTTAGAAGTTTATGGAGACGAAAAAGCAGAGTATAAGATCACTAGAGACCATATCAAGTCCTTGTTTGTGGATCTTTTCTTTGCAGGCACTGACACCTGGACGCACGCTATACAATGGATTATGGCCGAGATCATTAACAACTCGTACATTCTTGAGAGATTGAGAGAAGAAATTGATTCTGTTGTAGGAAAAACAAGGTTGATTCAAGAAACTGATCTACCGAACCTCCCGTGCTTGCAAGCAACCGTCAAAGAAGGGCTAAGGTTGCACCCTCCGGTTCCTCTTGTGTTAAGAACGTTTAAAGAAGGGTGTACGATTGGAGGCTTTTATGTTCCGGAGAAGACAACACTTGTTGTTAATGGGTATGCTATGATGAGAGATCCTGAATACTGGGAAGATCCTCAAGAATTTAAGCCAGAGAGATTTCTAGCTTCTTCAAGATCAAGCCAAAATGATGAGATAAGAGACGAACTCCTAAAATACCTTCCTTTCGGGAATGGAAGAAGAGCCTGTCCAGGAGCTAACCTAGCTTATATCTCTGTAGGAACCGCGATTGGAGTGATGGTTCAGTGCTTTGACTGGGAGATCAAAGGAGATAAGATCAACATGGATGAGGCTCCTGGATTGACCATGGCTCATCCTCTTAACTGCACTCTTGTT

>AL8G20150_v2_1

ATCACCGTTGACTTTGAAAACTGCTTCATCTTCGTCCTCTTATGTCTTTTGTCACGCCTCTTTTACGATCTTTTCTTCAGGAAACCAAAAGACTCACGAGGCTGTGATTTTCCTCCGAGCCCTCCATCATTACCGATCATTGGGCATCTTCACCTTATCCTCTTTGTTCCAATCCACCAGTCTTTCCAGAAAATCGCATCCAAATATGGACCTCTCCTCCACCTCCGCTTCTTTAACTTCCCCATAGTCCTTGTCTCCTCGCCCTCAATGGCCTACGAGATCTTCAAGGCCCAAGACGTGAATGTCTCCTCTCGCCCTCCTCCAATCGAGGAGTCTCTCATTTTAGGATCGTCCAGCTTCATCAACACTCCCTACGGAGATTACTCGAAATTCATGAAGAAGTTCATGGTCCAAAAGTTGCTCGGACCGCAGGCACTCCAACAGTCGCGAAAGATCCGTGCAGATGAGCTGGATCGGTTCTACAAAAACCTGCTTGATAAGGCGATGAAGAAGGAGTGTGTTGAGATTCGTAATGAAGCAATGAAGCTCACTAACAACACCATCTGCAAGATGATTATGGGGAGGAGTTGTTCCGAGGAGAACGGTGAGGCTGAGACAGTCAGAGGCTTGGTAACCGAGTCGATTTTCTTGACAAAGAAACATTTCTTGGGGGCCATGTTTCACAAACCGCTTAAGAAGCTCGGGATCTCATTGTTCGCAAAGGAATTAATGAGTGTTTCCAACAAGTTTGACGAGCTGCTGGAGAAGATTCTTGTTAAACATGAAGAGAAACAATGTACTGACATGTTGGATATGTTGTTGGAAGCGTATGGAGACGAAAAGGCAGAGTACAAGATCACTAGAGACCAAATCAAGTCCTTGTTCGTGGATCTGTTCAGTGCCGGCACTGAATCCTCGGCGAACACTATACAGTGGACCATGGCGGAGATCATTAACAATCCTAAGATTTCTGAGAGACTGAGAGAAGAAATTGATTCTGTTGTAGGTAATATAAGGTTGGTTCAAGAAACTGATCTACCGAACCTCCCTTACTTGCAAGCCATAGTCAAAGAAGGGCTACGATTGCATCCTCCAGGACCTGTG---GTAAGGACGTTCCAAGAAACATGTGAAATCAAAGGATTCTACATACCGGAGAAAACACGACTTTTTGTTAATATTTATGCTATAATGAGAGATCCTGATTTCTGGGAAGATCCTGAGGAGTTTAAACCAGAGAGGTTTTTAACTTCTTCAAGATTAGGGCAAGAAGATGAGAAAAGAGAGGACATGCTAAAATACATTCCTTTCGGTAGCGGAAGGAGAGCTTGTCCTGGATCACATCTAGCTTATGCCGTCGTTGGAAGTGTAATTGGAGTGATGGTGCAAAACTTTGATTGGAGAATCAAAGGAGAAAAAATCAACATGAAGGAG---GGTGGATTGACCATGGCTCAGCCTCTTCAGTGCACTCCTGTT

>AT5G47990_TAIR10

ATCACTGTTGACTTTGAAAACTGCTTCATCTTCCTCCTCTTATGTCTTTTCTCACGCCTCTCTTACGATCTTTTCTTCAGGAAAACAAAAGACTCACGAGGCTGTGCTCTTCCTCCGAGCCCTCCTTCATTACCAATCATTGGTCATCTTCACCTTATACTCTTTGTCCCAATCCACCAGTCTTTCAAGAACATCTCATCTAAATATGGACCTCTCCTCCACCTCCGCTTCTTTAACTTCCCCATAGTCCTCGTCTCCTCGGCCTCAACGGCCTACGAGATCTTCAAGGCCCAAGACGTGAATGTCTCCTCTCGCCCTCCTCCTATCGAGGAGTCTCTCATTTTAGGATCTTCCAGCTTCATCAACACTCCCTATGGAGATTACTCGAAGTTCATGAAGAAGTTCATGGTCCAAAAGCTGCTTGGACCACAAGCACTCCAACGGTCCCGAAATATCCGTGCAGATGAGCTGGAACGGTTCTACAAAACCCTGCTTGATAAGGCGATGAAGAAGCAGACTGTTGAGATTCGTAATGAAGCAATGAAGCTCACTAACAACACCATTTGCAAGATGATTATGGGGAGGAGTTGTTCAGAGGAGAACGGTGAGGCTGAGACAGTCAGAGGCTTGGTGACCGAGTCGATTTTCTTGACAAAGAAACATTTCTTGGGAGCCATGTTTCACAAACCACTTAAGAAGCTCGGGATCTCATTATTCGCAAAGGAATTGATGAATGTTTCCAACAGGTTTGATGAGCTACTGGAGAAGATTCTTGTTGAACATGAAGAGAAACAAACTAGTGACATGTTGGATATGTTGTTGGAAGCTTATGGAGACGAAAACGCAGAGTATAAGATCACTAGAGACCAAATCAAGTCTTTGTTCGTGGATCTGTTCAGTGCAGGCACTGAAGCCTCGGCGAACACTATACAGTGGACCATGGCGGAGATCATTAAGAATCCTAAGATTTGTGAGAGACTGAGAGAAGAAATTGATTCTGTTGTAGGGAAAACAAGGTTGGTTCAAGAGACTGATCTACCAAATCTCCCTTACTTGCAAGCAATAGTTAAAGAAGGGCTAAGATTGCATCCTCCGGGACCAGTG---GTAAGGACGTTCAAAGAGACATGTGAAATCAAAGGATTCTACATACCGGAGAAGACAAGACTTTTTGTTAATGTTTATGCGATAATGAGAGATCCTGATTTCTGGGAAGACCCTGAGGAGTTTAAGCCAGAGAGGTTTTTAGCTTCTTCAAGATTAGGGGAAGAGGATGAGAAAAGAGAGGACATGCTAAAATACATTCCTTTCGGTAGCGGAAGGAGAGCTTGTCCTGGATCACATCTAGCTTATACCGTTGTAGGAAGTGTAATTGGAATGATGGTGCAACACTTTGACTGGATAATCAAAGGAGAAAAAATCAACATGAAGGAG---GGTGGATTGACCATGGCTCACCCTCTTAAGTGCACTCCTGTT

>AL7G40290_v2_1

ATC------------------------ATCTTCATCCTCTTATGTCTCTTCACATTCCTCTGTTACTCTCTCTTCTTCATGAAACCAAAGGACTCACGAGACCGTGACCTTCCTCCGAGTCCTCCTTCTCTTCCTATCATCGGTCATCTTCACCTACTTCTCTCTACTTTAACCCACAAGTCTTTTCAGAGACTCTCATCCAAGTATGGACCTCTTCTCCATCTCCGCATCTTCCACGTTCCCATAGTCCTTGCCTCCTCGGCCTCAGTGGCCTACGATATCTTCAGGGACCAAGACGTGAATGTATCCTTTCGGCATCCTCCGATCGAGGAGTCTCTCTTTTTGGGATCGTATAGCTTCATCAGCGCTCCTTATGGAGATTACTGGAAATTCATGAGGAAGCTCATGGTTACAAAGATTCTTGGACCGCAAGCACTCCAGCGGTCACGAAGATTCCGTGAAGATGAGCTTGATCGGTTCTACAAAAATCTACTCGATAAGGCGAGTAAGAAGGAGATTGTTGAGATCGGTGAGGAAGCAGCGAAGCTCAATAACAACACCATCTGCAAGATGATCATGGGGAGGAGTTGTTCTGAGGAGAGCGGTGAGGCAGAGAGGGTCAGAGGCTTGGTGACTGAGTCAATGGCCTTGACAAAGAAAATTTTCTTGGCGACCATCTTTGACAAACCACTTAAGAAGCTCGGGATCTCACTGTTCAAAAAGGAGATAATGAGTGTTTCCCACAAGTTCGACGAGCTACTGGAGAAGATTCTTGTGGAACACGAAGAGAAACAAGGTACTGACATGATGGATGTGTTGTTGGAAGCTTATAAAGACGAAAACGCAGAGTATAAAATCACAAGAAACCATATCAAGTCCTTGTTTGTGGATCTTTTCATTGCAGGCACTGACACCTCATCGACCACTATACAGTGGATCATGGCGGAGATCATTAACCATCCCAAGATTCTTGAGAGACTAAGAGAAGAAATCAATTCCGTTGTAGGGAAAAGCAGATTGATTCAAGAAACTGACTTACCCAACCTCCCTTACTTGCAAGCAATAATCAAAGAAGGGCTAAGATTGCATCCGCCGGGGCCTCTCTTGCCAAGAACGGTCCAAGAAAGGTGTGAAATTAGAGGGTTCCACATACCAGAGAAGACAATTCTTATTGTTAATTCTTATGCTATAATGAGAGATCCTGATTATTGGGAAGATCCGGAGGAGTTTAAACCTGAGAGGTTTTTAGGTTTTCCAAGATCAGGACAAGAGGACGAGATAAGAGATAAATTCCTAAAATATATTCCTTTTGCCAGCGGAAGGAGAGGTTGTCCTGGAACAAATCTAGCTCATGTCTCTGTAGGAACCGCGGTTGGAGTGATGGTGCAGTGCTTTGATTGGAAAATCAAAGGAGAGAAAGTCAACATGAATGAGGCTGCTGGATTGACCATGGCTCACCCTCTTAAGTGCACTCCTGTT

>AT4G15350_TAIR10

ATC------------------------ATCTTCATCCTCTTATGTCTCTTGTCATTCCTATGTTACTCTCTCTTCTTCATGAAACCTAAAGATTCACGAGGCCGTGACCTTCCTCCGAGCCCTCCTTCTCTTCCAATCATCGGTCATCTTCACCTACTTCTGTCTACTTTAACCCACAAGTCTTTTCAGAGACTCTCATCAAAGTATGGGCCTCTTCTCCATCTCCGCATCTTCCACGTACCTATAGTCCTCGCCTCCTCAGCCTCAGTGGCCTACGAAATCTTCAGGGACCAAGACGTGAATGTATCATTTCGGCATCCTCCGATTGAAGAGTCTCTCTTTTTGGGGTCGTATAGCTTCATTAGCGCCCCCTATGGAGATTACTGGAAATTCATGAGGAAGCTCATGGTTACCAAGATTCTTGGACCGCAGGCACTCGAGCGGTCACGAAGATTCCGTGAAGATGAGCTTGATAGGTTTTATAAAACTCTGCTCGACAAGGCGATGAAGAAGGAGAGTGTTGAGATTGTTGAGGAAGCAGCGAAGCTCAATAACAACACCATCTGCAAGATGATCATGGGGAGGAGTTGTTCTGAGGAGACCGGTGAGGCGGAGAGAATCAGAGGCTTGGTGACTGAATCAATGGCTTTGACAAAGAAAATTTTCTTGGCGACCATCTTTCACAAACCGCTTAAGAAGCTCGGGATCTCACTTTTCAAAAAGGAGATAATGAGTGTTTCCCGCAAGTTCGATGAGCTACTAGAGAAGATTCTTGTGGAACACGAAGAGAAACAAGGTACTGATATGATGGATGTGTTGTTGGAAGCTTATCGAGACGAAAACGCTGAGTATAAGATCACAAGAAACCATATCAAGTCAATGTTTGTGGATCTTTTCATTGCTGGTACTGATACATCATCAACCACTATACAATGGATCATGGCGGAGATCATTAACCATCCCAAGATTCTTGAGAGGCTAAGAGAAGAAATCGATTTTGTTGTAGGGAAAACAAGGTTGATTCAAGAAACTGACCTACCGAACCTCCTTTACTTGCAAGCGATAATCAAAGAAGGGCTAAGATTGCATCCGCCGGGGCCACTCTTACCAAGAACGGTCCAAGAAAGGTGTGAAATTAAAGGGTTCCACATACCAGAGAAGACTATACTTGTTGTCAATTCTTATGCTATAATGAGAGATCCTGATTTTTGGGAAGATCCTGACGAGTTTAAGCCAGAGAGATTTTTATCTATTTCAAGATCAGGGCAAGAGGACGAGATAAGAGATAAATTCCTAAAATACATTCCTTTTGCCAGCGGCAGGAGAGGTTGTCCAGGAACAAATCTAGCTTATGCTTCTGTAGGAACCGCGGTTGGAGTGATGGTGCAATGCTTTGATTGGAAAATTGAAGGAGAGAATGTCAACATGAATGAGGCTGCTGGATTGACCATGGCTCACCCTCTTAAGTGCACTCCTGTT

>AT3G20950_TAIR10

ATCACTGTTGACTTTCAAAATAGCTTCATCTTCATCCTCTTTTGTCTCTTCTCTCTCATTTGTTACTCTCTCTTCTTCAGGAAACCAAAAGATTCACGAGGTCGTGATCTTCCTCCGAGCCCTCCTTCTTTTCCGGTTTCTAATAATCTTCACCTTCTTCTCTCTGCTCTTGTTCACAAGTCTTTTCAAAAAATCTCCTACAAGTATGGACCTCTCCTCCATCTCCGTGTCTTTCATGTTCCCATAGTCCTAGCCTCCTCGGCCTCAGTGGCCTACGAAATTTTCAAGGCCCAAGATGTGAATGTCTCCTCTCGCGGTGCTCCAGCCGGGGAGTCTCTCTTGTTTGGATCTTCTAGCTTCTTCTTCGCTCCCTATGGAGATTACTTTAAATTTATGAGGAAGCTCATAGCCACAAAGCTTCTTGGACCGCAGGCACTCGAGCGTTCAAGAAAAATCCGTGCAGATGAGCTAGATCGGTTTTACAGAAATCTGCTTGACAAGGCGATGAAGAAGGAGAGTGTCGATATCGTTGAGGAAGCAGCGAAGCTTAATAACAATATCATCTGCAAGATGATCATGGGGAGGAGTTGTTCTGAGGACAACGGTGAGGCGGAGAGAGTCAGAGGCTTGGTGATTGAGTCTACGGCCTTGACAAAGCAAATCTTCTTGGGTATGATCTTTGATAAACCGCTTAAGAAGCTTGGGATCTCATTGTTCCAAAAGGATATAAAAAGTGTTTCCCGC---TTCGACGAGCTGCTGGAGAAGATTCTTGTTGAACACGAAGAGAGAAAAGCTAATGACATGATGGATTTGTTGTTGGAAGCTTATGGAGATGAAAATGCAGAGTATAAAATCACAAGAAACCATATCAAGTCCTTGTTCGTGGATCTTGTCATTGCCGGCACTGACACCTCGGCGCAAACAATAGAGTGGACAATGGCGGAGCTTATTAACAACCCTAACATTCTTGAGAGATTGAGAGAAGAAATAGAATCGGTTGTAGGGAACACAAGGTTAGTTCAAGAAACCGATCTACCAAACCTCCCTTACTTGCAAGCGGTAGTCAAAGAAGGGCTAAGGTTGCATCCGCCAGGGGCTGTGTTCTTAAGAACGTTCCAAGAAAGGTGTGAACTCAAAGGGTTCTACATACCGGAAAAGACATTGCTTGTTGTTAATGTTTATGCTATAATGAGAGATCCTAAGCTTTGGGAAGATCCTGAGGAGTTTAAACCTGAGAGGTTTATAGCATCTTCAAGATCAGGGCAAGAGGACGAGATAAGAGAGGAAGTCCTGAAATACATGCCTTTCTCAACTGGAAGGAGAGGCTGTCCTGGATCCAATCTAGCTTATGTCTCTGTAGGAACCGCGATTGGAGTAATGGCGCAATGCTTTGATTGGAGAATCAAAGGAGAGAAAGTAAACATGAACGAGGCTGCTGGATTGACTATGGCTCAGCCTCTTATGTGCACTCCTGGT

>AT3G20940_TAIR10

ATCACCTTTGACTTTCAAAACAGCTTCATCTTCATCCTCTTTTTTCTCTTCTCTCTCCTTTGTTACTCTCTCTTCTTCAGGAAACCAAAGGGCTCACGAGGCCGTGATCTTCCTCCGAGCCCTCCTTCTTTTCCTGTAATTGGTCATCTTCACCTTCTTCTCTCTGCTCTTGTTCACAAGTCTTTTCAAAACATCTCCTCTAAGTATGGACCTCTTCTCCATCTCCGCGTCTTCCACATTCCCATAGTCCTCGCCTCCTCGGCCTCAGTGGCCTACGAGATCTTCAAGGCCCAAGATGTGAATGTCTCCTCTCGCGGTGCTCCAGTCGGGGAGTCTCTCTGGTTTGGATCGTCTAGCTTCTTCTTCGCTCCCTATGGAGATTACTTTAAATTTATGAGGAAGCTCATAGCCACAAAGCTTCTTGGACCACAGGCACTCGAGCGTTCACGAAAAATCCGTGCAGATGAGCTAGATCGGTTTTACAAAACCTTGCTTGACAAGGCGATGAAGAAGGAGAGTGTTGAGATCGGTGAGGAAGCAGCGAAGCTCAATAACAATATCATCTGCAAGATGATCATGGGGAGGAGTTGTTCAGAGGAGAATGGTGAGGCGGAGAAATTCAGACACTTGGTGATCGAGTCGATGGCTTTGACAAAGCAAATCTTCTTTGGTATGATCTTTCACAAACCGCTTAAAAAGCTCGGGATCTCACTGTTCCAAAAGGATATACTAAGTCTTTCCCGCAAGTTCGACGAGTTGCTGGAGAAGATTCTTTTTGAACACGAAGAGAAACAAGCTAATGACATGATGGATTTTTTGTTGGAAGCTTATGGAGACGAAAATGCAGAGTATAAAATCACAAGAAACCATATCAAGTCCTTGTTCGTGGATCTTGTCATTGCAGGGACTGACACCTCGGTGCAAGCAACACAGTGGACAATGGGGGAGCTCATTAACAACCCTAAGATTCTTCAGAGATTGAGAGAAGAAATCGAATCGGTTGTAGGGAACACAAGATTAATTCAAGAAAATGATTTACCAAACCTCCCTTACTTGCAAGCGGTAGTCAAAGAAGGGCTAAGATTGCATCCGCCGGGGTCTATCTCGGTAAGGATGTTTCAAGAAAGGTGTGAACTCAAAGGGTTCTACATACCGGAGAAGACATTACTTGTTGTTAATACTTATGCTATAATGAGAGATCCTAATTTCTGGGAAGATCCTGAGGAGTTTAAACCTGAGAGGTTTATAGCATCTTCAAGATCAGAGCAAGAGGACGAGGTAAGAGAGGAAGTCTTGAAATACATTCCTTTCTCCGCGGGAAGGAGAGGCTGTCCTGGATCAAATCTAGCTTATATCTCTCTAGGAATCGTGATTGGGGTAATGGTGCAGTGCTTTGATTGGAGAATTGAAGGAGAGAAAGTAAACATGAATGAGGCTGCTGAATTGTCCATGGCTCAGCCTCTTAAGTGCACTCCTGTT

>AT3G20130_TAIR10

ATCAGCTTTGACTTTCAGAACTGCTTCATCTTCATCCTCATATTTCTCTTAACGTTCCTATGCTTCTTTTTCTTCTTCAAGAAACCAAAGGATTCACGAAACTTCGATTTGCCTCCGAGCCCTCCTTCTCTACCAATCATTGGTCATGTTCACCTTCTCCTCTCTACTCTAACCCACAAGTCATTACAGAAACTCTCCTCCAGGTATGGACCTCTCCTCTATCTCCGGATTTTTAATGTCCCCATCATCCTCGTTTCCTCGGCCTCAGTGGCCTACGAGATCTTCAGGACACAAGACGTGAACATCTCCTCTCGCGGTACCGCGGTTGATGAGTCCCTCGTGTTTGGATCTTCCAGTTTCGTCACTGCTCCTTACGGAGATTACTGGAAGTTCATGAAGAAGCTTACTGTCATGAAGCTTCTCGGACCGCAGGCACAAGAGCAGTCACGAGACATCCGTGCAGATGACATAAAGCGGTTTTGCAGGAATCTGCTCGATAAAGCAAGGAAGAAGGAGAGCGTTGAGATTGGTAAAGAAGCAATGAATCTCATGAACAACATTTTGTGCAAGATGAGCATGGGAAGGAGTTTTTCAGAGGAGAATGGTGAGACAGAGAAACTAAGGGGATTAGTTACCGAGTCGATTGGCTTGATGAAAAAGATGTTCTTGGCAGTTTTGTTGCGCAGACAGCTTCAGAAACTTGGAATCTCACTATTCAAGAAGGATATCATGGGCGTTTCCAACAAATTTGATGTGCTGCTAGAGAAAGTTCTTGTGGAACATAGAGAGAAACAAGGTACGGTTATGTTGGACGTGTTGTTGGCAGCTTATGGAGACGAAAATGCAGAGTACAAGATCACTAAGAATCACATCAAGGCTTTTTTCGTGGACCTTTTCATTGGAGCCACTGATACCTCTGTGCAAACAATACAGTGGACAATGGCCGAGATCATGAACAATACTCACATTCTTGAGAGAATGAGAGAAGAAATTGATTCTGTCGTAGGGAAATCAAGGTTGATTCAAGAAACGGATTTACCGAACCTCCCTTACTTGCACGCTGTCATTAAGGAAGCACTAAGACTGCACCCACCGGGGCCTCTCTTGCCAAGGGAATTTCAACAAGGGTGTAAGATCGGAGGGTTCTACATACCGGAGAAGACAACACTTTTGATTAATGCCTATGTTGTGATGAGAGATCCGAATGTCTGGGAAGACCCTGAGGAGTTTAAACCAGAGAGATTTCTAGCTTCTTCAAGGTCAGGGCAAGAGGACGAGAGAAGAGAGCAAGCTCTTAAGTTTCTTCCTTTCGGCAGTGGAAGGAGAGGATGTCCTGGATCAAATCTAGCTTATATGATTGTAGGAAGTGCAATAGGAATGATGGTGCAGTGCTTTGACTGGAGAATCGAAGGAGAGAAGGTCAACATGAAAGAGGCTGTTAAACTTACCATGGCTCATCCTCTTAAGTTAACTCCTGTC

>AL3G34900_v2_1

ATCAGCGTTGACTTTCAGAACTGTTTCATCTTCATCTTCCTGTGCCTCTTCTCAATCGTCTGTTACTCTATCTTTTTCAAGAAACCAAAGGACTCACGAGGCTGTAATCTACCTCCCAGCCCTCCGTCTCTTCCAATCATCGGTCATCTTCACCTTCTCCTCTCTTCTCTATCCCACAAGTCTTTACAGAAAATCTCCTCCAAGTATGGACCCCTCCTCCATCTCCGGATTTTTAACGTCCCCATCATCCTTGTTTCGTCTGCCTCAGTGGCTGACGAGGTCTTCAGGGCCCACGATGTGAACGTCTCCTCTCGTGGTGCTGCGATAGATGAGTCCCTCGTGTTTGGATCTTCTGGTGTCGTCTACGCTCCCTACGGAGATTACTTGAAGTTCGTGAAGAAGATCATCGCCACTAAGCTTCTCCGACCTCAGGTGTTGGAACGGTCACGAGGTCTTCGTGCTGAAGAGCTACAACAGTTTTACAACAGAATTCTTGACAAGGCGAGGAAGAATGAGAACGTTGAGATTGGTAAGGAAGCGACGATGCTCATGAACAACATCTTGTGCAGGATGAGCATGGGAAGGAGTTTCTCAGAGGAGAATGGTGAGGCAGAGAGAGTCAGGGGATTGGTGGGTGAATCATATGCCTTGGCGAAGAAGATTTTCTTCGCATCTGTACTGCGAAGACCGCTTAAGAAGCTCGGTATCCCACTATTCAAGAAGGATATAATGGATGTTTCCAACAGATTCGATGAGCTGCTAGAGAAGATTCTTGTGGAACACAAAGAGAAAAAAGATACGGATATGATGGACGTGTTGTTGGCAGCTTATGCAGACGAAAATGCAGAGTATAAGATCACTAGGAATCATATCAAATCGTTTTTTGTGGAGCTTTTCGTGGGAGGCACTGATACCTCGGTGCAAACAACACAATGGACAATGGCCGAGATCATTAATAAGCCTGACGTTCTTGTGAGGCTGAGAGAAGAAATCGATTCCGTTGTAGGGACATCAAGGTTGATTCAAGAAACAGATATACCAAACCTTCCTTATTTGCAAGCGGTGGTTAAGGAAGGACTAAGATTGCACCCACCATTTCCTCTGCTGACGAGGAAGTTTGAAGAAAGGTGTGAGATCAAAGGGTTCTACATTCCGGAGAAGACATTTCTTGTTATTAATGCTTATGCTTGGATGAGAGATCCTGATTCCTGGGAAGATCCTAATGAGTTTAAGCCGGAGAGGTTTCTAGGATCTTCAAGATTAGGACAAGAAGACGAG---AGGGACGAAGCTCAGAAGTACATTCCTTTTGGGGGCGGAAGGAGAGGCTGTCCTGGATCTAATCTAGCTTCTATTTTCATAGGAACCGCGATTGGAGTGATGGTTCAGTGCTTTGACTGGGGAATCAAAGGAGATAAGGTCAATATGGAAGAGACTTTTGAACTTACCATGGTTCATCCACTTAAGTGCACTCCAGTT

>AT4G15360_TAIR10

ATCATCGTTGAGTTCCAAAACTTCTTTATCTTCATCCTTCTATGCCTCTTCTCACTCCTCTGTCACTCTCTCTTCTTCAAGAAACCAAAAGACTCACGAAGCTTTGTTCTGCCTTCAAGCCCTCCTTCTCTTCCAATCATCGGCCATCTTCACCTTCTCCTCTCTGTCCTAACTCACAAGTCTCTTCAGAAACTCTCCTCCAAGTACGGACCTCTTCTCCTTATCCGCATCTTCTATGTACCCATCATTCTTGTCTCCTCTTCCTCTATGGCCTACGAGATCTTCAAGGCCCATGACGTGAACGTCTCTTCTCGCGGTATTGCTCTTGATGAGTCTCTCATGTTTGGGGCTTCTGGCATCTTGAACGCTCCCTATGGAGATTACTGGAAGTTCATGAAGAAGCTCATGGCCACTAAGCTACTCCGACCGCAAGTGCTGGAGCGGTCACGAGGTGTTCGTGTTGAAGAGCTACATAGATTTTACAGGAGCATTCTTGATAAGGCGACGAAGAATGAGAGCGTTGAGATCGGTAAGGAAGCGATGAAGCTCATGAACAACACCTTGTGTAAACTGATCATGGGAAGGAGTTTTTCAGAGGACAACGGTGAATCAAATAGAGTCAGGGGCTTGGTTGATGAAACCTATGCCTTGTCCGAGAAGATATTCTTGGCAGCTATATTGCGCAGACCTCTTGCAAAACTTCGGATCTCACTATTCAAGAAAGAGATAATGGGTGTCTCCAACAAATTCGATGAGCTACTAGAGAGGATCCTTCAGGAACGCAAAGAGAATGAAGGTATGGATATGATGGACGTGTTGTTGGAAGCTTATGGAGATGAAAACGCTGAGTATAAGATCACATGGAAGCATATCAAGGCATTTTTCGTGGAGTTTTTCATTGGAGGGACTGATACCTCAGTGCAAACAACACAATGGGCAATGGCCGAGATGATCAATAACGCTAACGTTCTTGAGAGACTGAGAGAAGAAATCGTCTCCGTTGTAGGGGAAACAAGGTTGATCCAAGAAACAGATTTACCAAACCTACCTTATTTGCAAGCAGTGGTTAAGGAAGTTCTAAGATTGCACCCACCATCACCGGTCTTAATACGGAAGTTCCAAGAAAAATGTGAGGTCAAAGGATTTTACATACCGGAGAAGACAACACTCATTGTTAATGTTTACGCTATAATGAGAGATTCTGATTCTTGGGAAGATCCTGAGAAATTTAAGCCAGAGAGGTTTCTAACTTCTTCAAGATCAGGGGAAGAGGATGAAAAAGAG---------CTTAAGTTTCTTCCTTTTGGCAGCGGAAGGAGAGGATGTCCTGGAGCAAACCTTGGTTCTATATTTGTAGGAACCGCAATAGGAGTGATGGTGCAGTGCTTTGACTGGAAAATCAAAGAAGATAAGGTCAACATGGAAGAGACTTTTGAACTGAAAATGGTTCATCCGCTTACTTGCACTCCGTTC;

((AT3G20110_TAIR10:0.14723,AL3G33800_v2_1:0.14865):0.06987,((AT4G15330_TAIR10:0.22097,(AL1G42060_v2_1:0.03104,AT1G28430_TAIR10:0.04193):0.34772):0.09464,(AT3G20130_TAIR10:0.19107,(AL3G34900_v2_1:0.14324,AT4G15360_TAIR10:0.18215):0.05597):0.02):0.02189,((((AT4G15380_TAIR10:0.16019,(AL3G33780_v2_1:0.06929,AT3G20080_TAIR10:0.05621):0.03526):0.15781,(AL3G33810_v2_1:0.0654,AT3G20140_TAIR10:0.08963):0.11497):0.01624,((AT2G27000_TAIR10:0.10896,AT2G27010_TAIR10:0.12372):0.15248,((AL8G20150_v2_1{Foreground}:0.03822,AT5G47990_TAIR10{Foreground}:0.04613):0.16535,((AL7G40290_v2_1:0.03808,AT4G15350_TAIR10:0.02748):0.07984,(AT3G20950_TAIR10:0.06033,AT3G20940_TAIR10:0.07522):0.09304):0.0214):0.03262):0.05139):0.01492,(((AT3G20100_TAIR10:0.19004,(AL1G58200_v2_1:0.0492,(AT1G50520_TAIR10:0.11408,AT1G50560_TAIR10:0.09147):0.03427):0.29983):0.03078,(AT2G14100_TAIR10:0.09875,AT3G32047_TAIR10:0.1037):0.13706):0.00985,(AT5G42580_TAIR10:0.32,AL3G33760_v2_1:0.17763):0.008):0.01688):0.00627)

**D) THAA codon-based alignment and input for Newick tree**

>AT1G24430_TAIR10

CTTGAGATCACGGTAACTTCTCAGGAACTCGTCAAGCCCTCTCCTCGAAACCTTAATCATCATCATCTCTCTTTCCTTGATCAGCTTGCTCCTCCCATTTTCATGCCTTTCCTTTTCTTTTACTCAGACAAAGAACGAAGTGATCACATCAAGAGTTCTTTGTCTGAAATATTGAATCTCTATTACCCCTTGGCAGGACGCATCGATGTTGTTGTGTGCAACGATGTGGGTGTGTCTTTCGTCGAAGCCAAAGCCGATATGTCACAGATTCTAGAAAACCCAAATCCTAACGAACTTAACAAGCTTCATCCAGACGTGCCT---CTCACGGTACAGCTCACTTTCTTTGAATGTGGCGGCTTAGCACTCGGAATAGGCCTTTCTCATAAACTCTGTGATGCACTGTCTGGTCTCATCTTCGTCAACAGTTGGGCACCTTCTTTTGATCTCGCCAAGATGTTTCCTAAGGAGAACATAGTAACCAGACGCTTCGTGTTCTTGAGATCTTCTGTTGAGTCTTTAAGAGAAAGATTCAGCGGAAAGATCCGCGCGACACGTGTTGAGGTCTTATCGGTATTCATATGGAGCCGTTTCATGGCTTCAACCGGAAAGATCTATACACTGATTCATCCAGTGAACTTGCGTAGACAAGCAGATCCAATACCAGACAACATGTTCGGGAACATCATGAGGTTCTCTGTTACTGTCGAAAATGATGAAGAAAAGGCTTCTCTTGTGGATCAGATGAGAGAGGAGATTAGAAAGATAGACGCAGTTTACGAAGATAACAGAGGACACCTAGAGGTATCGTTTAGCTTCACGAGCCTCTGCAAATTTCCGGTGTATGAAGCAGATTTCGGATGGGGGAAACCTTTGTGGGTTTTCATTGATACTAAGGAAGGTGATGGAATTGAAGCTTGGATCAACCTTGACCAGAACGATATGTCTAGATTCGAAGCCGATGAGGAGTTGCTTCGCTATGTCTCTTCAAACCCAAGTGTGATG

>AL1G40510_v2_1

CTTGAGATCACAGTGACTTCTCAGGTTCTCGTCAAGCCCTCTTCTCGAAATCTTAATCATCATCATCTCTCTTTCCTTGATCAGCTTGCTCCACCTATCTTCATGCCTTTCCTTTTCTTTTACTCAGACAAAGAACGAAGTGATCACTTCAAGAGTTCCTTGTCAGAAATATTGAATCTCTATTACCCCTTGGCAGGACGCATCGATGTTGTTGTATGTAACGATATGGGTGTGTCTTTTGTCGAAGCCAAAGCCGATATGTCACAGATTCTAGTAAACCCTAATCCCAATGAACTCAACAAGTTTCTTCCAGACGTGCCT---CTAAGGGTACAGGTCACTTTCTTTGAATGTGGCGGCTTAGCACTCGGAGTAGGCCTTTCTCATAAACTCTGTGATGCCTTGTCCGGCCTCATCTTCATCAAAAGTTGGGCACCTTCTTTCGATCTCGCCAAGATGTTTCCGAAGGAGAACGTAGTAACCAAACGCTTTGTGTTCTTGAAATCCTCTGTTGAGTCTTTAAGAGAAAGATTCAGCGGAAAGATTCGCGCCACACGTGTTGAGGCCTTATCGGTATTCATATGGAGCCGTTTCATGGCTTCACCCGGAAAGATCTATACATTGATTCATCCAGTGAACTTGCGTAGACAAGCAGATCCGATACCAGACAACATGTTCGGGAACATCATGAGGTTCTCTGTTACTGTCGAACATGATGAAGAAAAAGCTTCTCTTGTGGAGCAGATGAGAGAGGAGATTAGAAAGATTGATGCAGTGTTCGAAGATAACAGAGGACACCTAGAGGTGTCGTTCAGCTTCACGAGCCTCTGCAAATTTCCGGTTTATGAAGCAGATTTCGGATGGGGGAAACCTTTGTGGGTTTTCATTGATACTAAGGAAGGTGATGGCATTGAGGCTTGGATCAACCTTGACCAGAACGATATGTCTAGATTCGAAGCCGATGAGGAGTTGCTTCGCTATGTCTCCTCAAACCCAAGTGTGATT

>AT5G23970_TAIR10

ATGAAGCTAGAACTCTTAAGCAAAGAAGTAATCAAACCCGCTTCACCTAATCATCTCCAACTTTCTCTTTCTCTCTTCGACCAGTTTCTTCCTTCAACTTACGTTTCCGCCATTTTCTTTTACTCAAACCAAGAAGACATCCAGAGGCTCAAAAGCTCACTCTCTCAGACTCTGTCTCTTTTTTATCCACTCGCTGGACAAATCGTTACTGTCCATTGCAACGATGAGGGAGCTTTGTTTACTGAGGCACGGGCTGAACTCTCGGATTTTCTGAGAAACCCAGATGCTGATTTGATTCAAAAGTTCATTGTCACATGGCCGTTGTTGCACGTCAAGGTCATCTTCTTTAAAGACAAAGGATTTGCGGTTGCAGTTAGCGTGTCGCACAAGATATGCGATGCGGCCTCGTTGTCAACGTTTGTTTGCAGCTGGACACCTGAATTTGTGGGGGCCGATTTCTACCCTGAAACAAAATCTAAGACAAAGAGGTTTGTCTTTGGTTCTTTGATGATTGAAAAGCTTAAAAACAGAGCTTCAAGTGTACCACAAGCTACCCGTATTGAGTCCATCACGGCGCTGCTGTTAAGATGCATGACAAAAGCAAAGGTGAAAGAATTTGCAATAACACAGACAATGGACTTGCGACCTAGAGTTTCTTCCTTGCCGCACAAGGCAATTGGAAAC---TTCTTCTTTTTACCATTACTTGAGAGTAAGATGGAAATCGAAGAAACGGTGTCTAAGCTGCAGAAAACTAAACAGGAGCTGAATGAGCTTATCAGCGTTGAAGCCAAAGAGAGAATCGAGACATATGCTGTGTCTAGCTGGTGCAGAATGTCGTTTTACGAGGCGAATTTTGGATGGGGGAAGCCGGTTTGGGTTTTAATGGACTCAAAGGACAGTGAAGGGGTTGAGGCAAGGGTTACACTACCTGAAACCGACATGGCTAAGTATGAGCATGATAGTGAACTACTCGTTTACGCTACTCCAAGTCCTAGTATCCTC

>AL6G35290_v2_1

ATGAAGCTAGAATTCGTAAGCAAAGAAATAATCAAACCCGCTTCACCTAATCATCCCCAACTTTCTCTTTCTTTCTTCGACCAGTTTCTTCCTTCAACTTACGTTTCCGCCATTTTCTTCTACCAAGAAGACATAATCGTCCAGAGGCTCAAGAGCTCACTCTCTCAGACTCTGTCTCTTTTTTATCCACTCGCTGGACAAATCGTCACTGTTCATTGCAACGACCAAGGAGCTTTGTTTACAGAGGCACGGGCTGATCTCTCTGATTTCCTGAGAAATCCAGATGCTGATTTGGTTCACGAGTTCATTGTCACCTGGCCGTTGTTGCATGTCAAGGTCATTTTCTTTAAAGACAGAGGATTTGCGGTTGCAGTTAGCGTGTCTCACAAGATATGCGATGCGGCCTCGTTGTCAACGTTTGTTTGCAGCTGGACACCTGAATTTCCGGGGCCCGATTTCTACCCTAAAACAAAATCTAAGACAAAAAGGTTTGTCTTTGGTTCTTTGATGATTGAAAAGCTAAAAAACAGAGCTTCTAGTGAGCCACAAGTCACCCGTATTGAGTCCATCACGGCGCTGCTGTTAAGATGCATGACAAAGGCAAAGGTAACAAAATTTGCAATAACACAGACCATGAACTTGCGAACTAGAGTTTCTTCCTTGCCGCACAAGGCAATTGGAAAC---TTCTTCTTTTTACCATTACTTGAGAGTAAGATGGAAATCGAAGAAACTGTTTCTAAGCTGCAGAAAACTAAAGAGGAGCTCAATCAGCTTATCAGCGTTGAAGCCAAAGAGAGAATCGAGACATATGTTGTGTCTAGTTGGTGCAGGATGTCGTTTTACGAGGCGGATTTTGGATGGGGAAAGCCGGTTTGGGTTTTAATGGATGCAAAGGACAGTGAAGGGATTGAGGCATGGGTTACACTACCTGAAACCGACATGGTTGAGTTTGAGCATGATGATGAGCTGCTCGCTTACGCTACTATAAGTCCTAGTGTCCTC

>AT5G47980_TAIR10

CTCAATCTAGAGGTGATCCAAAGAGAAGTGATCAAACCTTCATCACCTGCACCTCATGATCTTCAACTATCAGTCATCGATTTTGGCATTGCAGAAGCTTGCGTGCCCATGATCTTCTTCTACTCCCCGGATATTGTCTCAACAAGGCTGAGAAGCTCTCTATCCCAGGCGCTGTCACGTTTCTATCCTCTCGCTGGAAAAAAAGTCTCCATCAGCTGTAACGACGAAGGAGCTGTGTTCACAGAGGCACGCACAAATTTGTCTGAGTTCCTAAGAAACATCGATATCAACTCTCTAAAGATTTTGATTCCGTCTCGGCCGTTGCTGAGTGTCCAGGCTACTTTCTTCGGGTCGGGAGGTCTTGCTGTTGGGATCTGCGTCTCTCACTGTATCTGTGACGCAGCCTCAGTGTCTACGTTTGTCCGAGGCTGGGCTCCTCAATTTGCAGAGGTGGCCATCCATCCTAGAGAAAAATGTGTCACAAATCGGTTTGTGTTTGAATCAGATAAGATCACAAAGCTCAAAATCGTGGCCGCCAGCGTGCCGTCCCCTACACGTGTGGAAGCCGTCATGTCACTTATATGGCGATGTGCCAGAAATGCCGTCCCAAGGGCCACAATGATGACTCAATCCATGGACTTGCGGCTTAGAATTCCTACTTTGTCACCTGACGCGATTGGAAAC---TTACAAGGGGTATTCTTTCTCGGGAGCGAGATAGAAATCAGTGAAGTCGTGGCTGAGTTTAGGAAGGAGAAAGAAGAGTTCAATGAGATGATAAACACTACACTTGGTCAGAAAATCGATACGTACACCATGTCTAGTTGGTGCAGAAAGGCTTTTTATGAGGTCGATTTCGGATGGGGTCGTCCAGCTTGGGTTTTGGTGGATGCTAAGGATGGTGAAGGTGTTGAAGTATGGGTAGGCATACCTGAACAAGACATGGCAGCTTTTGTCTGTGACCAAGAATTGCTTTCTTATGCATCTTTAAATCCCCCAGTCCTG

>AL8G20140_intronless

GCAAAGCTAGAGGTTGTAGGGAGAGAAATTATCAAACCTTCTTCATCCGCACATCATGATCTTCAACAGTCTTTAATAGACGTCTTTTTTCCTCAAATTTACGTTTCAGCAATCTTCTTCTACTCGCCGGCGATCATCTCCGGGAAGCTTAAAAGCTCTTTGTCGGAGACTCTGTCACGTTTCTATCCTCTAGCGGGAAGAATTTTCTCCATCAACTGCAACGACGAAGGAGCCGTGTTCACCGAAGCACGTACTGATCTCTCCGATTTCCTTAAAAACAACGATACCAACAATCTAGGAGAATTTTTCCCCACATGGCCATTGTTGAGTGTTAAGGTATGTTTCTTTGGATCCGGAGGGTTTGCGGTCACTGTAGCTACATCTCACCAAATCTGCGACGCAGCATCGTTGTTGACCTTTATCCAAAGCTGGGCTCCTCATTTTACAGGAGCTACCATTTACCCTAAAGGCAAGTGTGTTACCAATAGATTCGTCTTCAAATCCTCTAAGATTGCTGATCTCAAACGGAAGGCCGCTAGCGTTCCGGTACCTACACGTGTGGAAGCCATCACATCACTTATCTGGATATGTGCCACAAATGCCGCTGCAAAGTCAACGCTGATGAGTCAAGCCATGGACTTGCGGCTTAGGATTCCCTCGTTGTCACGAGACGCAATTGGTAAC---TTACAAACGGGATTCTTTCTCGAAAGCGAGATGGAAATTGGGAACATGGTTGCCAAGTTTAGAAAGGCCAAGGAAGGGGTCAACGAGATGATCAATACCACATTTGGTCAGAACTTGGACTTATACGCAATGTCTAGCTGGTGCAAAAAACATTTCTACAAGGTTGACTTTGGATGGGGTACTCCGGTATGGGTATTAATGGATTCAAAGGATGGTGAAGATGTGGAAGCTTGGGTAGGCTTACCCGAACAAGACATGCTCATGTTCGTTCGTGACCAAGACTTGCTTACTTATGCGGTCCTTAATCCTCCAGTTTTG

>AT4G15400_TAIR10

GCGAAGCTAGAGGTGACCGGGAAAGAAGTGATCAAACCTGCTTCACCATCACCTCGTGATCTTCAGCTCTCTATCCTTGATCTCTATTGTCCTGGAATCTACGTGTCGACGATCTTCTTCTACTCCTCGGAGGTTTTCTCGGAAAATCTGAAACTCTCTCTGTCCGAGACTCTCTCACGCTTCTATCCACTAGCTGGACGAATACTTTCCATCAGCTGTAACGACGAAGGAGCCGTCTTCACCGAGGCACGCACCGATCTTCCTGATTTCCTTAGAAACCTCAACACTGACTCCCTGTCGGGATTCCTCCCGGCCTGGCCGTTGTTGAGTGTCAAGGTCACTTTCTTCGGGTCTGGAGGAGTGGCGGTTTCTGTCTCTGTCTCTCACAAAATCTGTGATATAGCCTCATTGGTTACCTTTGTAAAAGACTGGGCT---GAGTTCGCTGAAACGACTATTTAT---ACAAGTAAATACGTACTCAAAAGATTCGTCTTCGAACCTTCTAAGATTGCTGAGCTGAAACACAAGGCCGCTAGCGTCCCTGTGCCTACACGTGTTGAAGCTATCATGTCACTTATCTGGAGATGTGCTAGAAACTCCATTCCAAGGCAAGCCGTCATGTGGCAGGCCATGGACATTCGACTTAGGATTCCATCTGCGCCAAAAGACGTGATTGGTAAC---CTACAAAGCGGATTTTCTCTCGAGAGCGAGTTTGAGATCCCTGAAATCGTGGCCACATTCAGGAAGAACAAAGAGAGAGTCAACGAGATGATCGGCAATACAATTGGTCAGAGTTTGGACCGATACATAATGTCTAGCTGGTGCAGAAAGCCTTTCTACGAGGTTGACTTCGGATCGGGTAGTCCGGTCTGGGTGTTGATTGATTCAAAAGAAGGTGATGGTGTAGAAGCATGGATAAGCTTACCTGAGGAAGACATGTCCGTGTTTGTCGATGACCAAGAGTTGCTTGCTTATGCCGTCCTAAATCCCCCGGTCGTG

>AL7G40200_v2_1

GCGAAGCTAGAGGTGGCTGCGAGAGAAGTGATCAAACCTGCTTCACCATCACCTCGTGATCTTCAACTCTCTATCCTCGATCTCTATTGTCCCGCAATCTACGTGTCGACGATCTTCTTCTACTCCCCGGAAATTTTGTCGGAAAATCTGAAAAGCTCTCTGTCCGAGACTTTGTCACGCTTCTATCCACTGGCTGGACGAATAGTTTCCATCAGCTGTAACGATGAAGGAGCCGTGTACACCGAGGCTCGCACCAATTTACCTGATTTCCTGAGAAACCTCAACACTGACTGCCTGTCGGGATTCCTCCCGGCTTGGCCCTTGTTGAGTGTCAAGGTCACTTTCTTCGGGTCGGGAGGGGTGGCGGTTTCTGTCTCTGTCTCTCACAAAATCTGTGATGCAGCCTCATTGGTGACCTTTGTTAAAGACTGGGCT---GAGTTCGCTGAAACGACTATTTATCCTGCAAATAAATGTGTAATCAAAAGATTCGTCTTCGAACCCTCTAAGATAGCTGAGCTCAGGCACAAGGCCGCTAGCGTTTCTGTACCTACACGTGTTGAAGCTATCATGTCACTTATTTGGAGATGTGCCAGAAAATCCATTCCAAGGCAAACAGTGATGTGGCAGGCCATGGACTTGCGGCTTAGGATTCCATCTTTGCCACAAGACGTGATTGGCAAC---CTACAATCAGGATTCTCTCTCGAGAGCGAGTTTGAGATCCCCGAAATCGTGGCGGCATTCAGGAAGACCAAAGAAGGAGTCAACGAGATGATCAGCAATACAACTGGTCAGAGTTTGGACAGATACATAATGTCTAGCTGGTGCAGAAAGCCATTCTACGAGGTTGACTTCGGATCTGGTAGTCCGGTCTGGGTGTTGATTGATTCAAAAGAAGGTGATGGTGTAGAAGCATGGATAAGCTTACCTGAGGAAGACATGTCTGTGTTTGTCGATGACCAAGAGTTGCTTGCTTATGCTGTCCTAAATCCCCCGGTCTTG

>AT3G26040_TAIR10

ATGAGAGTTGATGTTGTCTCCAGAGATATCATCAAACCATCATCTCCAACTCCAAACCACTTTAAACTCTCTCTTTTAGAACAGCTCGGTCCAACGATCTTTGGTCCTATGGTTTTCTTCTATAAACCAACTGAGCAATTGCAGATGCTGAAGAAGTCGTTATCCGAAACTTTAACTCATTTCTACCCTCTCGCTGGACGGCTCATAAGTATCGATTGTAATGACTCAGGCGCTGATTTCCTCGAAGCACGAGTCAATCTTTCGAATCTCCTACTAGAGCCTTCTTCTGACAGCTTGCAACAACTGATTCCTCGAACCAGACTGCTTCTTGCTCAAGCGAGTTTCTTTGAATGTGGAAGCATGTCTATAGGAGTTTGTATCTCTCATAAACTCGCTGACGCAACTTCTATCGGTTTATTCATGAAGAGCTGGGCTCCGGTTTTCGACACGGTTAAGATCTTCCCAATGAATCAGACTCTTTCGAAGAGATTCATATTCGATTCTTCGAGTATTCAAGCTCTGCAAGCGAAAGCTTCAAGCGTGAATCAACCAACAAGAGTCGAAGCTGTTTCAGCTCTTATATGGAAATCTGCAATGAAAGCTACATCGAAACCGTCAATTCTGGCAAACTCCGTGAGTCTACGCTCACGAGTCTCTCCATTTACAAAGAACTCAATAGGGAATCTAGTGAGCTACTTTGCAGCAAAAATAAACCAAACAAAGCTTCAAACTTTGGTTTCGAAAATACGAAAAGCGAAACAGAGGTTTCGAGATATCCAT------------GGAAACCCAAATGATTTCTACATATTCTCAAGTGCTTGTCGATTCGGTTTATACGAAACCGATTTCGGTTGGGGAAAACCGGTTTGGGTCCTTCTCGACACGAAAGAAGCTGGTGGAATTGAAGCTTGGGTGAATCTGAATGAACAAGAGATGAATCTTTTTGAACAAGATAGAGAATTGCTCCAATTCGCTTCTCTGAATCCTAGTGTGATC

>AL5G14720_v2_1

ATGAGAGTTGATGTTATCTCCAGAGATATCATCAAACCGTCATCTCCAACTCCAAACCACTTTCAACTCTCTCTTTTAGAACAGCTCGGTCCAACGATCTTTGGTCCTATGGTTTTCTTCTATAAACCAGCTGAGCAATTGCAGAAGCTGAAGAAGTCGTTATCTGAAACTTTAACCCATTTCTACCCTCTCGCTGGACGGCTCATAAGTATCGATTGTAATGACTCCGGCGCTGATTTCCTCGAAGCAGAAGTCAATCTTTCGAGTCTCCTACAAGAGCCTTCTTCGGACAGCTTGCAACAACTGATTCCTAGAACCAGACTGTTTCTTGCTCAAGCTAGTTTCTTTGAATGTGGAAGCATGGCTATAGGAGTTTGTATCTCTCATAAACTCGCTGACGCAACTTCTATCGGTTTATTCATGAAGAGCTGGGCTCCAGTGTTTGACACGGCCAAGATCTTCCCAATGAATCAGACTCTTTCGAAGAGATTTGTATTCGATTCTTCGAGCATTCAAGCCCTGCAAGCAAAAGCTTCAAGCGTGAATCAACCTACAAGAGTCGAAGCTGTTTCAGCTCTTATATGGAAAACTGCAATGAAAGCTACATCGAAACCGTCGATTCTGGCTAACTCCGCGAGTCTACGCTCACGCGTCTCTCCATTCACAAAGAACTCAATCGGGAATCTAGTGAGCTACTTTGCAGCAAAAACAAACCAAACAAAGCTTCAAACTTTGGTTTCGAAAATACGAAAAGCGAAACAGTGGTTTCGCGATAACCAT------------GGAAACCCAAATGATTTCTACATCTTCTCGAGTGCTTGTCGGTTCGGTTTATACGATACCGATTTCGGTTGGGGAAAACCGGTTTGGGTCCTTCTCGACACGAAAGAAGCTGGTGGAATCGAAGCTTGGGTGAATCTATATGAACAAGAGATGAATCTTTTTGAACAAGATAGAGAATTGCTCCAATTTGCTTCTCTGAATCCTAGTGTGATC

>AT1G24420_TAIR10

AAGAACGTTGAGATCTTATCAAGAGAAATAGTCAAACCGTCTTCTCCAACTCCAGATGATCTCAATCTCTCTCTTCTTGATATCCTCAGCTCACCAATGTACACAGGAGCTCTTCTCTTTTACTCAACAGAGGAGACTTCCTTGAAGCTCAAGAAATCTCTGTCTAAAACCTTACCAATCTTCTACCCTCTTGCCGGAAGAATCAGTTTCGTCGAATGTAATGATGAAGGAGCTGTGTTTATAGAAGCTCGAGTTGACCTCTCGGAGTTTCTCAAGTGCCCTGTTCCTGAATCATTGGAATTACTCATTCCTACATGGCCTGTGTTGTTAATCCAAGCTAATTTCTTCAGCTGTGGAGGATTGGTTATCACAATCTGCGTTTCTCATAAGATCACTGATGCTACCTCTTTAGCAATGTTCATCAGAGGATGGGCTCCTAGTTTCACCGCTTCTGAAGTCTTTCCTGAGATGAGTTGTGTGACAAAGAGGTTTGTGTTTGATGCTTCGAAGATCAAGAAACTTAGAGCCAAAGCTTCAAGAGTGAAGAATCCAACACGTGTTGAAGCCGTCACAGCTCTGTTTTGGAGATGCGTTACTAAGGTTACACCAAGAACTTCAGTGCTGCAGATACTAGTGAACCTAAGAGGAAAGGTAGATTCTTTGTGTGAAAACACAATTGGGAAT---ATGCTTTCCCTCATGATTCTCGCTGCGATTGAAAGAATTCAAGATGTGGTTGATGAGATAAGGAGGGCAAAGGAAATATTCAGCTTGAACTGCTCTTCGTCTAGAATTTTTGAGCTTGATTTGTGGATGAGTAATAGCTGGTGTAAGCTCGGCTTGTACGATGCTGATTTCGGATGGGGAAAGCCGGTTTGGGTGTTGATTGATACCAAAGATGGAGAAGGAATTGAAGCTTGGATCACTCTTACGGAAGAACAAATGTCACTGTTTGAATGTGATCAAGAGCTTCTTGAATCAGCTTCCCTAAATCCCCCTGTTTTA

>AL1G40520_v2_1

AAGAAAGTTGAGATCCTATCAAGAGAAATAATCAAACCGTCTTCTCCAACTCCAAATGATCTCAATCTCTCTCTTCTTGATGTACTCAGCTCACCGATGTACACAGGAGCACTTCTCTTTTACGAATCAGAGGAGACATCCTTGAAGCTCAAGAAATCTCTGTCTGAAACTTTACCAATCTTCTACCCTCTTGCCGGAAGAATCAGTTTCGTCGAATGTAATGATGAAGGAGCTGTGTTTATAGAAGCTCGAGTTGACCTCTCGGAGTTTCTCAAGTGCCCTGTTCCTGAATCATTGGAACTACTCATTCCTACATGGCCTGTCTTGCTAATCCAAGCCAGTTTCTTCAGCTGCGGAGGATTAGTTATCACAATTTGCATTTCTCATAAGATCACTGATGCAACCTCTTTAGCCATGTTCATCAGAGGATGGTCTCCTAGTTTCACTGCTGCGGAGTTCTTTCCTGAGATGAGTTGTGTGACAAAGAGGTTTGTGTTTGATGCTTCAAAAATCAAGAAACTCAGAGCCGAAGTTTCAAGCGTCAAGAATCCTACCCGTGTTGAAGCCGTCACAGCTCTGTATTGGAGATGCGTTACTAAGGCTACACCAAGAACTTCGGTGCTGCAGATACTAGTGAGCCTACGAGGAAAGGTAGATTCTTTGTGTGAAAACACAATTGGGAAT---ATGCTTTCCCTCATAATTCTCGCTAAGATAGATAGAATTCAAGATGTGGTTGATGAGCTAAGACGGGCAAAGGAAATCTTCAGCTTGAATTGCTCCTCGTCGAGAATTTTTGAGCTTGACTTGTGGATGAGTAATAGCTGGTGTAAGCTCGGCATGTACGAGGCCGATTTCGGATGGGGAAAGCCGGTTTGGGTGTTGATTGATACCAAAGATGGAGAAGGAATTGAAGCTTGGATCACTCTTACAGAAGAACACATGTCGCTGTTCGAATGTGATCAAGAACTTCTTGAATCAGCTTCCCTGAATCCCCCTGTTTTA

>AT5G47950_TAIR10

ATGAAGGTAGAAACTATTGGTAAAGAGATCATTAAGCCATCTGCAACAACTCCAACTGATCTTCAACTCTCTATTATGGATATACTAATGCCTCCGGTTTACGCCGTTGCCTTTCTTTTCTACTCGCAAGAACAAACGTCTCATACTCTCAAAACTTCCCTGTCCGAGATCTTGACAAAATTCCACCCTTTGGCCGGTAGAGTCGTCACCATCAAGAGTACCGACGAAGGAGCCGTCTTTGTGGAGGCACGCGTCGATCTCTCTGGTTTTCTGAGATCCCCGGATACCGAATCACTCAAACAGTTGCTACCTACATGGCCATTGCTTCTTGTGAAAGCAACCTACTTCCGATGTGGTGGCATGGCCATAGGACTCTGCATCTCCCATAGGCTCGCCGATGCAGCCTCTCTCTCGATTTTCTTACAGGCTTGGGCACCTGACTTTTGTTCCACAAAACTTTACCCCAACAGAACAAGTGTCACGAAGAGATTTGTGTTTGTAGCATCTAAAATCGAAGAGCTCAGGAACAAAGTCGCTAGTGTGCCTCGACCCACGCGGGTCCAGAGCGTGACTTCACTTATATGGAAATGTGTTGTGACTGCAACAATTCGTGAGAAAGCTCTGTTCCAACCGGCTAACTTGCGCACCAAGATACCTTCCTTGTCTGAAAACCAAATCGGTAACCTCTTGTTTAACTCCTTAATCTTGAAAGCCGGGATGGATATTGTAGAAACTGTTAAGGAACTACAAAAAAGAGCCGAGGATTTATCCGGTTTGGTTTCAATGACTATAGGTTGGAGATTGGACATGCATTCTGTAACTAGCTGGTGCAAGATTCCTCTTTATGATGCTTGCTTCGGGTGGGGATCTCCGGTTTGGGTATTGATCGATTCTAAAGACGGACAAGGAATCGAAGCATGGGTGACATTGCCTCAAGAGAACATGTTACTGTTGGAGCAGAGCACAGAACTGCTTGCCTTTGCCTCCCCAAATCCAAGTGTATTG

>AL8G20050_v2_1

ATGAAGGTTGAAATCATTGGTAAAGAGATCATAAAGCCATCTGCAATAACTCCAACTGATCTTCAATTCTCTATTATGGATATAATAATGCCTCCTGTTTACACCGTTGCCTTTCTTTTCTACTCCCAAGAACAAACGTCTCACACTCTCAAAACTTCCCTGTCCGAGATCTTGACAAAATTCCACCCTTTAGCCGGAAGAGTCGTCACCATCGAGAGTAATGACGAAGGAGCCGTCTTTGTGGAGGCACGCGTCGATCTCTCTAGTTTTCTTAGATCCCCAGATACCGAGTTCCTCAAACAGTTGCTTCCTACATGGCCCTTGCTTCTTGTGCAAGCGACCTACTTCCAATGTGGTGGCATGGCCATCGGACTCTGCATCTCCCATAAGCTTGCCGATGCAACCTCTCTCTCGATTTTCTTACAGGCTTGGGCTCCTGACTTTGTTTCCACAAAGCTTTACCCAAAGAGAACAAGTGTCACGAAGAGATTTGTGTTTGTAGAATCTAAGATCGAAGAGCTCAGGAACAAAGTCGCTAGTGTGCCTCGACCCACGCGGGTCCAGAGCGTGACTTCACTTATATGGAAATGTGTTGTGACTGCTACAATTCGTGAGAAAGCTCTGTTCCAACCGGCTAACTTGCGCCCCAAGATACCCTCCTTGTCTGAAAACCAAATCGGTAACCTCTTCTTCGCCACCCTAACCTTGAAAGCCGGGGTGGATATTGTAGAAACTGTTAAGGAACTACAAAAAAGGGCCGAGGAGTTATCCGGTTTGGTTTCAATGACGATAGGTTCGAGATTGGACATGCATTCTGTAACTAGCTGGTGCAAGATTCCTCTTTATAAGGCTTGCTTCGGGTGGGGATCTCCGGTTTGGGTATTGATCGATTCTAAAGATGGACAAGGAATCGAAGCATGGGTGACATTGCATCAAGAGAACATGTTACTGTTCGAGCAGAGCACAGAACTGCTTGCCTTTGCCTCCCCAAATCCAAGTGTATTG

>AT3G30280_TAIR10

ATGAAGGTAGAAACAATTGCTAAGGAAATCATAAAACCATCTTCCACAACTCCAAATGATCTCCAACTCTCTATTTATGATCACATCCTCCCTCCTGTTTACACAGTCGCCTTTCTCTTCTACTCTCCTGAACAAAGTTCTCACAAACTCAAGACTTCTCTTGCCGAAACCTTGACAAAATTCTACCCTCTTGCAGGCAGAATCGTCACCATCGATTGTAACGACGAAGGAGCTGTTTTTGTTGACGCTCGTGTCAATCTCTCTGATTTCCTGAGATCTCCGGATTTTAAAACCCTCCAACAGTTACTTCCTACATGGCCTTTGCTACTCGTGAAGGCGACTTACTTTCCATGTGGTGGCATGGCCATAGGATTATGCATCACTCACAAAATCGCGGACGCAACCTCCATCTCAACTTTCATTCAGTCTTGGGCTCCCGTGTTTGCGGCAGCAAATTTCTACCCAAAGATAAGCCAAATAACGAAGAGATTCGTGTTCAGTGCGTCTAAGTTGGAAGAGCTCAGGACCAAAGCAGCTAGTGTAGGCCGGCCTAAGCGGGTGGAAAGTGTCACTGCGCTTCTTTGGAAAGCCTTTGTTGCAGCTGCTTGTGATATGAAGGTATTGATCCAGCCCTCTAACTTGCGCCCCAAGATACCTTCTTTGGCAGAAAGCCTGATCGGAAACATAATGTTCTCTTCTGTGGTCTTGCAAGAGGAAATTAAAATTGAAAAGGCAGTTCGAGACTTACGAAAACAGGGAGATGACTTGCAAAATGTAATCTCTGCGATGATTGGTTCTAAACTAGAACCCTACACCGTGAGTAGCTGGTGCAAACTACCCCTTTATGAGGCCAGTTTCGGATGGGGATCACCGGTTTGGATTTTGATTGATTCCAAGGATGGACAAGGAATTGAAGCGTTTGTCACATTGCCCGAAGAGAACATGTTGTCTTTAGAGCAGAACACAGAACTCCTAGCCTTTGCTTCCGTGAATCCTAGTGTCTTG

>AL5G19920_v2_1

ATGAAGGTTGAAACAATCGCTAAAGAAATCATAAAACCATCTTCCACAACTCCAAATGATCTCCAACTCTCTATTTATGATCACATTCTCCCTCCTGTTTACACAGTCGCATTTCTCTTCTACTCTCCAGAACACAGTTCCCAAAAACTCAAGACTTCTCTGTCCGAAACCTTGACCAAATTCTACCCTCTTGCTGGCAGAATCGTCACCATAGATTGTAACAACGAAGGAGCTGTTTTTGTTGACGCTCGTGTCAATCTCTATGATTTCCTGAGATCTCCGGATTTCAAAACCCTCCAGCAGTTACTTCCTACATGGCCTTTGCTGCTAGTGAAGGCGACTTACTTTCCATGTGGTGGCATGGCCATAGGAATCTGCATCACTCACAAAATCGCGGACGCAACCTCCATCTCGACTTTCATTCAGTCTTGGGCTCCCGAGTTTGCGGCAGCAAATTTCTACCCAAAGATAAGTAGAATAACGAAGAGATTCGTGTTCAGTGCGTCCAAGTTGGAAGAGCTCAGGACCAATGCCGCTAGTGTAGCCCGACCTACACGGGTGGAAAGCGTCACTGCACTTCTTTGGAAAGCCATCGTTGAAGCTGCTTGTGATATGAAGGTCTTGATCCAGCCCTCTAACTTGCGCCCCAAGATACCGTCCCTACCTGAAAGCCTGATTGGAAATATAATGTTCTCTTCTGTCGTCTTGGAAGAGGAAGTTAAAATTGAAAAGGCCGTTAGAGACTTACGAAAAAAGGGAGATGACTTGCAATATGTAATCTCTTCGATGATTGGTTCTAAACTAGAGCCCTACACCGTGAGTAGCTGGTGCAAATTACCTCTTTATGAGGCAAGTTTTGGATGGGGATCACCGGTTTGGGTTTTGATTGATTCCAAGGACGGACAAGGAATTGAAGCGTTTGTCACATTGCCTGAAGAGAACATGATGTCTTTCGAGCAGAACACAGAACTCCTAGCCTTTGCTTCCGTTAATCCTAGTGTCTTG

>AL7G41110_v2_1

ATGAAGGTTGAAACCATC---------------------------------------------------------------------------------------ATAGCCTCTCTCTTCTACTCTCGAGAACACATTTCCCACAAACTCAAGGCTTCTCTGTCCGAAACCTTGACCAAGTTCTACCCTCTTTCCGGTAGAATCGTAACCATAGATTGTAACGATGAAGGAGCTATCTTTGTCGATGCTCGTGTCGATCTCTCTGGATTTCTCAGGTGCCCTGATTTCGAGGCCCTCCAACAGTTGCTTCCTACATGGCCTTTGCTACTCGTGAAGGCAACTTACTTCCAATGTGGAGGCATGGTCATAGGAATCTGCATCTCTCACAAGATTGCCGACGCAACCTCTATCTCTACTTTCATTCAGACATGGGCTCCCGAGTTTGCTGCAGCAGATTTCTACCCAAAGAGGAGCAGCATCACGAAGCGATTCGTTTTCGATGCTTCTAAGTTGGAACAGCTCAGGACCAAAGCTGCTAGTGTAGCCAGACCTACGTGGGTGGAGAGCGTCACTGCGCTTCTCTGGAAAGACTTGGTCGCTGCTACAAGTGATCAAAAAGTGCTGCTCCAGCCCGCTAACTTGCGCCCCAAGATACCTTCCCTGCCAGAAAGCCTGTTCGGCAATGTCATGTTTACTTCTGTAGTCTTGGAGGGGGAAGTTAAAATCGAAGAGGCCGTTGGAGAGTTACGAAAAAAGGGTGAGGACTTACGATGTTTGATCTCTTCGATGATTGGTTCTAAACTAGAGCCCTACACCGTAAGTAGCTGGTGCAAGCTACCACTTTACAAGGCTCATTTTGGATGGGGATCTCCGGTTTGGGTTTTGATCGATTCCAAGGACGGACAAGGAATTGAAGCGTTTGTTACACTGTCTCAGGAGAACATGTCGTCTTTCGAGCAGAACCCAGAACTACTCGCCTTTGCCTCCTTGAATCCTAGTGTCTTG

>AT4G15390_TAIR10

ATGAAGGTTGAAACCATTAGTAAAGAAATCATAAAGCCATCTTCGCCAACTCCAAATAATCTCCAACTCTCAATTTACGATCACATCCTTCCTCCAGTTTACACAGTAGCCTTTCTCTTCTACTCTCAAGAACACACTTCCCACAAACTCAAGACTTCTCTGTCTGAAACCCTGACCAAGTTCTACCCTCTTGCCGGAAGAATCGTAACCGTCGATTGTACCGATGAAGGAGCTATCTTTGTCGATGCTCGTGTCAATCTCACTGAATTTCTCAAGTGCCCTGATTTCGACGCCCTCCAACAGTTGCTTCCTACGTGGCCTTTGCTGCTCGTGAAGGCAACTTACTTCGGATGCGGAGGCATGGCCATAGGAATCTGCATCACTCACAAAATCGCGGACGCAGCCTCCATCTCGACTTTCATTCGGTCCTGGGCCCCTGTATTTGCTGGTGCGAATTTCTACCCAAAGAGAAGCAGCATTACAAAGAGATTTGTGTTCGAAGCTTCTAAGGTGGAAGATCTCAGGACCAAAGCCGCTAGTGTAGACCAACCTACGCGGGTGGAGAGCGTTACTGCGCTCATCTGGAAATGCTTCGTCGCATCCACTTGTGATCACAAAGTGCTGGTCCAGCTTGCTAACTTGCGGTCCAAGATACCTTCCCTGCAAGAAAGCTCTATCGGAAATCTCATGTTCTCTTCTGTGGTCTTGGGAGGGGAAGTTAAAATTGAAGAGGCCGTTAGAGACTTACGAAAAAAAAAGGAGGAGTTAGGAACTGTGATCTCTTCCATGATCGGTTCGAAACTAGAACCCTATACCGTGAGTAGCTGGTGTAAGCTACCTCTTTACGAGGCTAGCTTTGGATGGGATTCTCCGGTTTGGGTTTTGATAGATTCCAAGGACGGACAAGGAATTGAAGCGTTCGTCACACTGCCTGAAGAGAACATGTCGTCCTTCGAGCAGAACCCAGAGTTGCTCGCCTTTGCTACCATGAACCCTAGTGTCTTG

>AL7G40230_v2_1

ATGAAGGTTGAAACCATCGGTAAAGAAATCATAAAGCCATCTTCCCCAACTCCAAATGATCTCCAACTCTCAATTTATGATCACATCCTTCCTCCGATTTACACAGTAGCCTTTCTCTTCTACTCTCAAGAACACACTTCCCACAAACTCAAGACTTCTCTGTCCGAAACCTTGACCAAGTTCTACCCTCTTGCCGGAAGAATCGTAACCGTAGATTGTAACGATGAAGGAACTATCTTTGTCGATGCTCGTGTCAATCTCTCTGATTTTTTCAAATGCCCTGGTTTCGACTCCCTCCAACAGTTGCTTCCTACCTGGCCTTTGCTGCTTGTGAAGGCAACTTACTTCCAATGTGGAGGCATGGCAATAGGAATCTGCATCTCTCATAAGATTGCCGACGCAACCTCTATCTCTAGTTTCATTAAGAGCTGGGCTACCGAGTTTGCCGCAGCAAACTTCTACCCAAAGAGAAGCAGTATCACGAAGAGATTTGTGTTCGATGCTTCTAAGTTAGAAGAGTTGAGCACTAAAGTCGCTAGTGTAGACAGACCTACACGGGTGGAGAGCGTCACTGCGCTTTTCTGGAAAGGGTTCGTCTCTGCGACTTGTGATCTGAAAGTGTTGATCCAACCAGTTAACTTGCGCTCCAAGATACCCTCGCTGTCACAAAACTTGATCGGAAATGTCATGTTCTCTTCTGTGGTTTTGGAAGGGGAAGTGAAAATTGAAGAGGCCGTTAGAGACTTACAGAAAAAGAAGAATGACTTACAAATTGTGATCTCTTCAATGATCGGTTCTAAACTTGAGCCCTACACCGTGAGTAGCTGGTGCAAGCTACCACTTTACGAGGCTAGTTTTGGATGGGGATCTCCAGTTTGGGTTTTGGTCGATTCCAAAGACAAAAAAGGAATTGAAGCGTTTGTCACACTGCCTGAAGAGAACATGTTGTCCTTTGAGCAGAACCCAGAATTGCTCGCCTTTGCTTCCCTGAATCCTAGTGTCTTG;

((AT4G15390_TAIR10:0.08468,((AT5G47950_TAIR10{Foreground}:0.04512,AL8G20050_v2_1{Foreground}:0.04131):0.24929,(((AT5G23970_TAIR10:0.07399,AL6G35290_v2_1:0.06195):0.39898,(AT5G47980_TAIR10:0.2994,(AL8G20140_intronless:0.20429,(AT4G15400_TAIR10:0.06546,AL7G40200_v2_1:0.02805):0.19099):0.04297):0.18614):0.21397,((AT1G24420_TAIR10:0.04884,AL1G40520_v2_1:0.02879):0.34793,((AT1G24430_TAIR10:0.0332,AL1G40510_v2_1:0.03059):0.78275,(AT3G26040_TAIR10:0.02519,AL5G14720_v2_1:0.02886):0.35592):0.223):0.11582):0.14683):0.09987):0.01899,AL7G40230_v2_1:0.07774,((AT3G30280_TAIR10:0.04352,AL5G19920_v2_1:0.0229):0.07331,AL7G41110_v2_1:0.10444):0.01913)
